# Supplementary material for: Structural analysis of an anthrol reductase inspires enantioselective synthesis of enantiopure hydroxycycloketones and β-halohydrins
Source: Nat Commun. 2023 Jan 21;14:353. doi: 10.1038/s41467-023-36064-4 (PMC9867772; doi:10.1038/s41467-023-36064-4)
Supplement: Supplementary file 1 — Supplementary Information [file 41467_2023_36064_MOESM1_ESM.pdf]

Supplementary Information

Structural analysis of an anthrol reductase inspires enantioselective synthesis of enantiopure hydroxycycloketones and β-halohydrins

Xiaodong Hou[1,#], Huibin Xu[1,#], Zhenbo Yuan[1], Zhiwei Deng[1], Kai Fu[1], Yue Gao[1], Changmei Liu[1], Yan Zhang[2], and Yijian Rao[1,*]

[1]Key Laboratory of Carbohydrate Chemistry and Biotechnology, Ministry of Education, School of Biotechnology, Jiangnan University, Wuxi 214122, P. R. China.

[2]School of Life Sciences and Health Engineering, Jiangnan University, Wuxi 214122, P. R. China.

[#]These authors contributed equally: Xiaodong Hou, Huibin Xu.

*Correspondence and requests for materials should be addressed to Y.R. (email: raoyijian@jiangnan.edu.cn)

Table of contents

Supplementary Notes 3
Supplementary Tables 12
Supplementary Figures 20

Supplementary Notes

The nucleotide and protein sequences of CbAR.

Nucleotide sequence of *CbAR* [807 bp]:

ATGTCGCCACCAACACAAGACTTGCACATTCCGGGCCGCCTCGATGGCAAAGTGGCAC
TTGTAACTGGGTCCGGACGAGGGATCGGTGCTGCAGTGGCAGTCCATCTAGGCCTCCT
GGGTGCTAAAGTCGTTGTCAACTACGCAAATTCTCCTACACACGCACAAAAAGTCGTA
GACGAGATTAAGCAACTGGGATCCGATGCCATCGCAATCAAGGCTGATGTTCGACAAG
TTCCCGAAATTGTCCGCCTTTTCGATGAGGCAGTCGCACATTTCGGTCAGCTGGACATC
GCAGTCAGCAACTCGGGCGTCGTCAGCTTCGGCCACCTGAAGGACGTTACGGAAGAG
GAATTCGATCGTGTATTCAGTCTCAACACTCGTGGCCAATTCTTCGTTGCTCGCGAAGC
TTACAAGCATTTGAACAACGGGGGCCGAATAATCATGACATCGTCCAACACTTCCAGA
GACTTCAGTGTCCCCAAGCACTCGCTATATTCCGGGTCAAAGGGCGCCATCGACAGCT
TTGTTCGGATCTTTTCCAAGGACTGCGGGGACAAGAAGATCACAGTGAATGCTGTGGC
TCCCGGAGGAACAGTAACCGACATGTTCCACGATGTCTCACAGCACTACATTCCCAAC
GGAGAAACATATACACCAGAGGAACGCCAGAAGATGGCGGCACATGCGTCACCTCTTC
ATCGTAACGGGTTTCCTGAGGATATCGCGCGTGTTGTCGGCTTCCTTGTCAGTGCAGAG
GGAGAGTGGATCAATGGGAAGGTGCTCACTGTAGATGGTGGTGCTGCGTGA

Protein sequence of CbAR [269 AA, 28.7 kDa]:

MSPPTQDLHIPGRLDGKVALVTGSGRGIGAAVAVHLGLLGAKVVVNYANSPTHAQKVVD
EIKQLGSDAIAIKADVRQVPEIVRLFDEAVAHFGQLDIAVSNSGVVSFGHLKDVTEEEFDRV
FSLNTRGQFFVAREAYKHLNNGGRIIMTSSNTSRDFSVPKHSLYSGSKGAIDSFVRIFSKDC
GDKKITVNAVAPGGTVTDMFHDVSQHYIPNGETYTPEERQKMAAHASPLHRNGFPEDIAR
VVGFLVSAEGEWINGKVLTVDGGAA

Characterization of substrates and products

2-[(17-Oxoestra-1,3,5(10)-trien-3-yl) oxy] acetonitrile (1c)

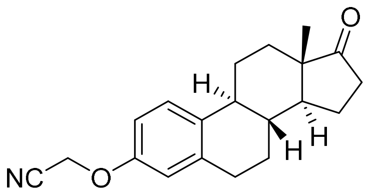

$C_{20}H_{23}NO_2$: 309.409 g/mol

^{1}H NMR (400 MHz, $CDCl_3$) δ 7.28 (d, *J* = 8.6 Hz, 1H), 6.81 (dd, *J* = 8.7, 2.8 Hz, 1H), 6.75 (d, *J* = 2.9 Hz, 1H), 4.76 (s, 2H), 2.94 – 2.92 (m, 2H), 2.53 (dd, *J* = 18.8, 8.6 Hz, 1H), 2.42 (d, *J* = 9.7 Hz, 1H), 2.31 – 2.27 (m, 1H), 2.22 – 1.97 (m, 4H), 1.71 – 1.45 (m, 6H), 0.94 (s, 3H) ppm.

^{13}C NMR (101 MHz, $CDCl_3$) δ 220.8, 154.6, 138.5, 134.7, 126.8, 115.3, 115.1, 112.5, 53.7, 50.4, 48.0, 44.0, 38.2, 35.9, 31.6, 29.6, 26.4, 25.9, 21.6, 13.9 ppm.

Exact Mass $[M+H]^+$: 310.1807 (calculated), 310.1817 (found).

Estrone acetate (1d)

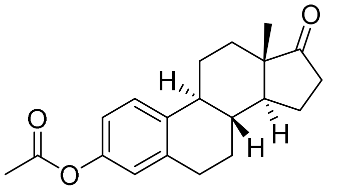

$C_{20}H_{24}O_3$: 312.409 g/mol

^{1}H NMR (400 MHz, $CDCl_3$) δ 7.30 (d, *J* = 8.6 Hz, 1H), 6.87 (d, *J* = 8.4 Hz, 1H), 6.83 (s, 1H), 2.93 – 2.91 (m, 2H), 2.52 (dd, *J* = 18.8, 8.7 Hz, 1H), 2.41 (d, *J* = 7.0 Hz, 1H), 2.30 (s, 4H), 2.20 – 1.96 (m, 4H), 1.66 – 1.43 (m, 6H), 0.92 (s, 3H) ppm.

^{13}C NMR (101 MHz, $CDCl_3$) δ 220.7, 169.8, 148.6, 138.0, 137.4, 126.4, 121.6, 118.8, 50.4, 47.9, 44.1, 38.0, 35.9, 31.6, 29.4, 26.3, 25.8, 21.6, 21.1, 13.8 ppm.

Exact Mass $[M+H]^+$: 313.1804 (calculated), 313.1794 (found).

2-methyl-2-(4-methylbenzyl) cyclopentane-1,3-dione (1e)

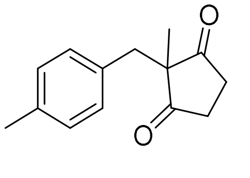

$C_{14}H_{16}O_2$: 216.280 g/mol

^{1}H NMR (400 MHz, $CDCl_3$) δ 7.04 (d, *J* = 7.9 Hz, 2H), 6.93 (d, *J* = 8.1 Hz, 2H), 2.92 (s, 2H), 2.58 – 2.52 (m, 2H), 2.29 (s, 3H), 2.12 – 2.05 (m, 2H), 1.19 (s, 3H) ppm.

2-methyl-2-(3-methylbenzyl) cyclopentane-1,3-dione (1f)

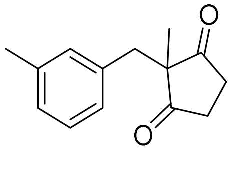

$C_{14}H_{16}O_2$: 216.280 g/mol

^{1}H NMR (400 MHz, $CDCl_3$) δ 7.04 (t, *J* = 7.5 Hz, 1H), 6.94 (d, *J* = 7.7 Hz, 1H), 6.78 – 6.74 (m, 2H), 2.83 (s, 2H), 2.51 – 2.44 (m, 2H), 2.21 (s, 3H), 2.03 – 1.96 (m, 2H), 1.12 (s, 3H) ppm.

2-methyl-2-(2-methylbenzyl) cyclopentane-1,3-dione (1g)

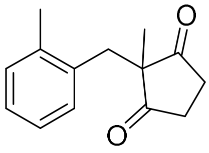

$C_{14}H_{16}O_2$: 216.280 g/mol

^{1}H NMR (400 MHz, $CDCl_3$) δ 7.11 – 7.09 (m, 2H), 7.08-7.04 (m. 1H), 6.98-6.95 (m, 1H), 3.03 (s, 2H), 2.57 – 2.50 (m, 2H), 2.26 (s, 3H), 2.17 – 2.11 (m, 2H), 1.21 (s, 3H) ppm.

2-methyl-2-(4-chlorobenzyl) cyclopentane-1,3-dione (1h)

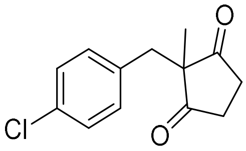

$C_{13}H_{13}ClO_2$: 236.695 g/mol

^{1}H NMR (400 MHz, $CDCl_3$) δ 7.20-7.16 (m, 2H), 6.98-6.95 (m, 2H), 2.91 (s, 2H), 2.64 – 2.58 (m, 2H), 2.15 – 2.09 (m, 2H), 1.18 (s, 3H) ppm.

2-methyl-2-(4-bromobenzyl) cyclopentane-1,3-dione (1i)

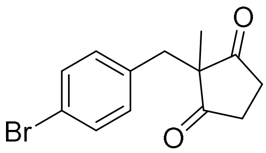

$C_{13}H_{13}BrO_2$: 236.695 g/mol

^{1}H NMR (400 MHz, $CDCl_3$) δ 7.36 (d, *J* = 8.1 Hz, 2H), 6.93 (d, *J* = 8.0 Hz, 2H), 2.93 (s, 2H), 2.67 – 2.60 (m, 2H), 2.18 – 2.12 (m, 2H), 1.21 (s, 3H) ppm.

2-benzyl-2-methylcyclohexane-1,3-dione (1j)

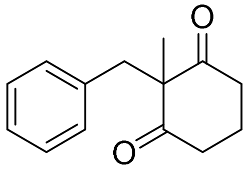

$C_{14}H_{16}O_2$: 216.280 g/mol

^{1}H NMR (600 MHz, $CDCl_3$) δ 7.27 - 7.17 (m, 3H), 7.10 – 7.04 (m, 2H), 3.12 (d, *J* = 2.0 Hz, 2H), 2.54 (dddd, *J* = 17.0, 7.6, 5.0, 2.1 Hz, 2H), 2.30 (dddd, *J* = 16.6, 9.0, 5.4, 2.1 Hz, 2H), 1.77 – 1.70 (m, 1H), 1.53 – 1.46 (m, 1H), 1.29 (d, *J* = 2.0 Hz, 3H) ppm.

2-bromo-1-(4-bromo-2-hydroxyphenyl)ethan-1-one (1o)

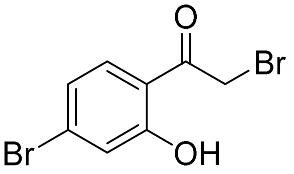

$C_8H_6Br_2O_2$: 293.942 g/mol

^{1}H NMR (400 MHz, $CDCl_3$) δ 11.82 (s, 1H), 7.63 (d, *J* = 8.6 Hz, 1H), 7.26 (d, *J* = 1.9 Hz, 1H), 7.11 (dd, *J* = 8.6, 1.9 Hz, 1H), 4.41 (s, 2H) ppm.

^{13}C NMR (101 MHz, $CDCl_3$) δ 196.6, 163.6, 132.0, 131.2, 122.9, 122.2, 115.9, 29.5 ppm.

(*R*)-3,8,9,10-tetrahydroxy-6-methyl-3,4-dihydroanthracene-1(2H)-one (2a)

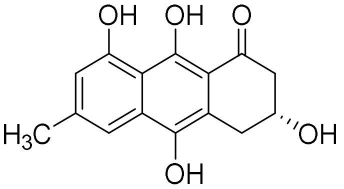

$C_{15}H_{14}O_5$: 274.272 g/mol

^{1}H NMR (400 MHz, acetone-*d_6*) δ 15.96 (s, 1H), 9.81 (s, 1H), 7.67 (s, 1H), 7.49 (s, 1H), 6.70 (s, 1H), 4.43 (d, *J* = 22.3 Hz, 2H), 3.28 (dd, *J* = 16.3, 3.7 Hz, 1H), 3.05 (dd, *J* = 10.6, 5.1 Hz, 1H), 3.00 (d, *J* = 3.6 Hz, 1H), 2.83 – 2.78 (m, 1H), 2.45 (s, 3H) ppm.

^{13}C NMR (101 MHz, acetone-*d_6*) δ 203.9, 159.3, 158.0, 142.8, 140.7, 133.1, 116.8, 112.5, 112.3, 110.7, 109.1, 65.1, 45.8, 31.7, 21.4 ppm.

Exact Mass $[M+H]^+$: 275.0919 (calculated), 275.0931 (found).

17β-estradiol (2b)

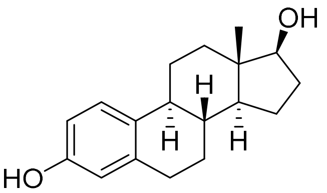

$C_{18}H_{24}O_2$: 272.388 g/mol

^{1}H NMR (400 MHz, DMSO-*d*$_6$) δ 8.99 (s, 1H), 7.03 (d, *J* = 8.3 Hz, 1H), 6.51 (dd, *J* = 8.4, 2.6 Hz, 1H), 6.43 (d, *J* = 2.6 Hz, 1H), 4.51 (d, *J* = 4.8 Hz, 1H), 3.52 (td, *J* = 8.5, 4.8 Hz, 1H), 2.72 – 2.68 (m, 2H), 2.22 (dd, *J* = 13.4, 3.5 Hz, 1H), 2.08 – 2.01 (m, 1H), 1.91 – 1.82 (m, 2H), 1.78 – 1.74 (m, 1H), 1.56 (dd, *J* = 12.1, 7.4 Hz, 1H), 1.42 – 1.04 (m, 7H), 0.66 (s, 3H) ppm.

Exact Mass [M+H]$^+$: 273.1855 (calculated), 273.1857 (found).

2-(((8R,9S,13S,14S,17S)-17-hydroxy-13-methyl-7,8,9,11,12,13,14,15,16,17-decahydro-6H-cyclopenta[a]phenanthren-3-yl)oxy)acetonitrile (2c)

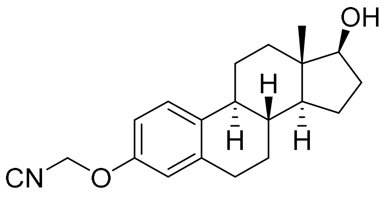

$C_{20}H_{25}NO_2$: 311.425 g/mol

^{1}H NMR (400 MHz, CDCl$_3$) δ 7.25 (d, *J* = 8.6 Hz, 1H), 6.77 (d, *J* = 8.3 Hz, 1H), 6.70 (s, 1H), 4.72 (s, 1H), 3.72 (t, *J* = 8.5 Hz, 1H), 2.87 – 2.84 (m, 2H), 2.31 (dd, *J* = 13.5, 3.8 Hz, 1H), 2.22 – 2.07 (m, 2H), 1.92 (dd, *J* = 24.9, 11.3 Hz, 2H), 1.73 – 1.69 (m, 2H), 1.54 – 1.14 (m, 8H), 0.78 (s, 3H) ppm.

^{13}C NMR (101 MHz, CDCl$_3$) δ 154.5, 138.7, 135.3, 126.8, 115.4, 115.1, 112.4, 81.8, 53.8, 50.0, 44.0, 43.2, 38.7, 36.7, 30.6, 29.8, 27.1, 26.3, 23.1, 11.1 ppm.

Exact Mass [M+H]$^+$: 312.1964 (calculated), 312.1967 (found).

17β-Estradiol 3-acetate (2d)

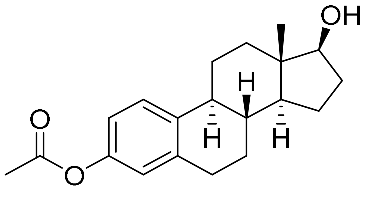

$C_{20}H_{26}O_3$: 314.425 g/mol

^{1}H NMR (400 MHz, CDCl$_3$) δ 7.31 (d, *J* = 8.4 Hz, 1H), 6.86 (dd, *J* = 8.5, 2.6 Hz, 1H), 6.81 (d, *J* = 2.6 Hz, 1H), 3.75 (t, *J* = 8.5 Hz, 1H), 2.91 – 2.87 (m, 2H), 2.35 (ddd, *J* = 13.4, 7.2, 4.2 Hz, 1H), 2.30 (s, 3H), 2.24 (td, *J* = 11.2, 4.4 Hz, 1H), 2.17 – 2.10 (m, 1H), 1.98 (dt, *J* = 12.7, 3.5 Hz, 1H), 1.91 (ddt, *J* = 11.5, 5.7, 3.0 Hz, 1H), 1.73 (dddd, *J* = 12.5, 9.9, 7.0, 3.1 Hz, 1H), 1.57 – 1.31 (m, 7H), 1.26 – 1.18 (m, 1H), 0.80 (s, 3H) ppm.

^{13}C NMR (101 MHz, $CDCl_3$) δ 169.9, 148.4, 138.3, 138.0, 126.4, 121.5, 118.6, 81.9, 50.1, 44.1, 43.2, 38.5, 36.7, 30.6, 29.5, 27.0, 26.2, 23.1, 21.2, 11.1 ppm.

Exact Mass [M+H]$^+$: 315.1960 (calculated), 315.1965 (found).

(2*S*, 3*S*)-3-hydroxy-2-methyl-2-(4-methylbenzyl) cyclopentan-1-one (2e)

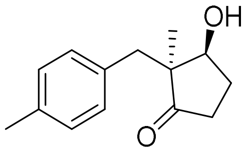

$C_{14}H_{18}O_2$: 218.296 g/mol

^{1}H NMR (400 MHz, $CDCl_3$) δ 7.18 (d, *J* = 8.0 Hz, 2H), 7.10 (d, *J* = 7.9 Hz, 2H), 4.07 (t, *J* = 3.7 Hz, 1H),3.02 (d, *J* = 13.8 Hz, 1H), 2.72 (d, *J* = 13.8 Hz, 1H), 2.57-2.48 (m, 1H), 2.42-2.36 (m, 1H), 2.34 (s, 3H), 2.24-2.15 (m, 1H), 1.94-1.86 (m, 1H), 1.83 (s, 1H), 0.91 (s, 3H) ppm.

^{13}C NMR (101 MHz, $CDCl_3$) δ 220.5, 135.8, 134.7, 130.2, 128.9, 76.6, 54.8, 35.4, 33.8, 28.2, 21.0, 19.7 ppm.

Exact Mass [M+Na]$^+$: 241.1204 (calculated), 241.1235 (found).

(2*S*, 3*S*)-3-hydroxy-2-methyl-2-(3-methylbenzyl) cyclopentan-1-one (2f)

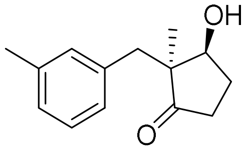

$C_{14}H_{18}O_2$: 218.296 g/mol

^{1}H NMR (400 MHz, $CDCl_3$) δ 7.21-7.17 (m, 1H), 7.10-7.04 (m, 3H), 4.09 (t, *J* = 3.5 Hz, 1H), 3.03 (d, *J* = 13.8 Hz, 1H), 2.72 (d, *J* = 13.7 Hz, 1H), 2.58-2.46 (m, 1H), 2.43-2.37 (m, 1H), 2.35 (s, 3H), 2.25-2.16 (m, 1H), 1.94-1.87 (m, 1H), 1.82 (s, 1H), 0.91 (s, 3H) ppm.

^{13}C NMR (101 MHz, $CDCl_3$) δ 220.4, 137.8, 137.7, 131.1, 128.1, 127.4, 127.0, 76.5, 54.8, 35.7, 33.7, 28.2, 21.5, 19.7 ppm.

Exact Mass [M+Na]$^+$: 241.1204(calculated), 241.1178(found).

(2*S*, 3*S*)-3-hydroxy-2-methyl-2-(2-methylbenzyl) cyclopentane-1-one (2g)

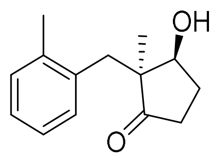

$C_{14}H_{18}O_2$: 218.296 g/mol

^{1}H NMR (400 MHz, $CDCl_3$) δ 7.36 – 7.32 (m, 1H), 7.18 – 7.11 (m, 3H), 4.19 (t, *J* = 3.8 Hz, 1H), 2.98 (s, 2H), 2.61-2.52 (m, 1H), 2.43-2.40 (m, 1H), 2.38 (s, 3H), 2.29-2.20 (m, 1H), 2.02-1.95 (m, 2H), 0.88 (s, 3H) ppm.

^{13}C NMR (101 MHz, $CDCl_3$) δ 220.3, 137.3, 136.4, 130.5, 130.5, 126.3, 125.7, 76.5, 55.3, 33.6, 30.7, 28.1, 20.3, 18.9 ppm.

Exact Mass [M+Na]$^+$: 241.1204(calculated), 241.1235(found).

(2*S*, 3*S*)-3-hydroxy-2-methyl-2-(4-chlorobenzyl) cyclopentan-1-one (2h)

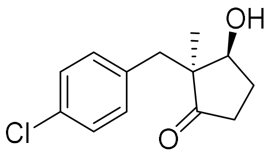

$C_{13}H_{15}ClO_2$: 238.711 g/mol

^{1}H NMR (400 MHz, $CDCl_3$) δ 7.27-7.22 (m, 4H), 4.05 (t, *J* = 3.4 Hz, 1H), 3.05 (d, *J* = 13.7 Hz, 1H), 2.70 (d, *J* = 13.7 Hz, 1H), 2.58-2.48 (m, 1H), 2.44-2.36 (m, 1H), 2.26-2.17 (m, 1H), 1.94-1.86 (m, 1H), 1.70 (s, 1H), 0.86 (s, 3H) ppm.

^{13}C NMR (101 MHz, $CDCl_3$) δ 220.1, 136.5, 132.1, 131.8, 128.2, 76.2, 54.6, 35.1, 33.6, 28.5, 19.5 ppm.

Exact Mass [M+Na]$^+$: 261.0658 (calculated), 261.0640 (found).

(2R,3S)-3-hydroxy-2-methyl-2-(4-bromobenzyl) cyclopentane-1-one (2i)

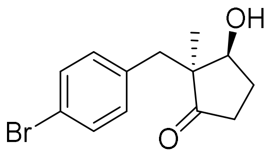

$C_{13}H_{15}BrO_2$: 283.1650 g/mol

^{1}H NMR (400 MHz, $CDCl_3$) δ 7.41 (d, *J* = 8.4 Hz, 2H), 7.18 (d, *J* = 8.4 Hz, 2H), 4.06 – 4.04 (m, 1H), 3.03 (d, *J* = 13.7 Hz, 1H), 2.68 (d, *J* = 13.7 Hz, 1H), 2.59-2.47 (m, 1H), 2.45-2.36 (m, 1H), 2.27-2.16 (m, 1H), 1.94-1.86 (m, 1H), 1.73 (s, 1H), 0.86 (s, 3H) ppm.

^{13}C NMR (101 MHz, $CDCl_3$) δ 220.1, 137.0, 132.2, 131.2, 120.2, 76.2, 54.5, 35.1, 33.6, 29.7, 28.5, 19.5 ppm.

Exact Mass [M+Na]$^+$: 305.0153(calculated), 305.0182(found).

(2*S*, 3*S*)-2-benzyl-3-hydroxy-2-methylcyclohexan-1-one (2j)

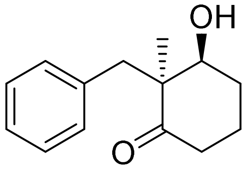

$C_{14}H_{18}O_2$: 218.296 g/mol

^{1}H NMR (600 MHz, $CDCl_3$) δ 7.25 (t, *J* = 6.7 Hz, 2H), 7.23 – 7.14 (m, 3H), 3.77 – 3.76 (m, 1H), 3.11 (d, *J* = 13.8 Hz, 1H), 2.97 (d, *J* = 13.8 Hz, 1H), 2.62 – 2.48 (m, 2H), 2.18 – 2.09 (m, 1H), 2.06 - 2.01 (m, 1H), 1.87 (dtd, *J* = 13.7, 6.5, 4.2 Hz, 1H), 1.79 (dtd, *J* = 13.4, 6.6, 4.5 Hz, 1H), 1.08 (s, 3H) ppm.

^{13}C NMR (151 MHz, $CDCl_3$) δ 213.7, 137.5, 130.5, 128.1, 126.3, 75.7, 54.5, 37.7, 37.3, 28.5, 20.7, 20.4 ppm.

Exact Mass [M-H_2O+H]$^+$: 201.1279 (calculated), 201.1278 (found).

(*R*)-2-chloro-1-phenylethan-1-ol (2l)

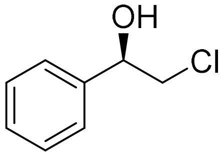

C_8H_9ClO: 156.609 g/mol

^{1}H NMR (600 MHz, $CDCl_3$) δ 7.42 – 7.31 (m, 5H), 4.90 (m, 1H), 3.76 – 3.72 (m, 1H), 3.67 – 3.62 (m, 1H), 2.67 (d, *J* = 3.0 Hz, 1H) ppm.

^{13}C NMR (151 MHz, $CDCl_3$) δ 139.9, 128.7, 128.5, 126.1, 74.1, 50.9 ppm.

Exact Mass [M-H_2O+H]$^+$: 139.0314 (calculated), 139.0315 (found).

(*R*)-2-bromo-1-phenylethan-1-ol (2m)

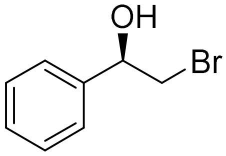

C_8H_9BrO: 201.063 g/mol

^{1}H NMR (600 MHz, $CDCl_3$) δ 7.37 – 7.31 (m, 5H), 4.91 (dt, *J* = 9.0, 3.2 Hz, 1H), 3.62 (dd, *J* = 10.5, 3.3 Hz, 1H), 3.53 (dd, *J* = 10.5, 9.0 Hz, 1H), 2.71 (d, *J* = 3.2 Hz, 1H) ppm.

^{13}C NMR (151 MHz, $CDCl_3$) δ 140.3, 128.7, 128.5, 126.0, 73.8, 40.2 ppm.

Exact Mass [M-H_2O+H]$^+$: 182.9809 (calculated), 182.9804 (found).

(*R*)-2-chloro-1-(4-methoxyphenyl)ethan-1-ol (2n)

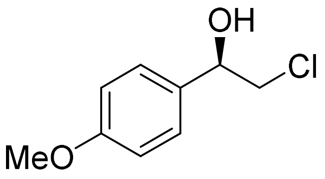

$C_9H_{11}ClO_2$: 186.635 g/mol

^{1}H NMR (400 MHz, $CDCl_3$) δ 7.36 – 7.27 (m, 2H), 6.95 – 6.89 (m, 2H), 4.86 (dt, *J* = 8.7, 3.3 Hz, 1H), 3.83 (s, 3H), 3.74 – 3.62 (m, 2H), 2.77 (d, *J* = 3.0 Hz, 1H) ppm.

^{13}C NMR (101 MHz, $CDCl_3$) δ 159.7, 132.1, 127.4, 114.1, 73.7, 55.4, 55.3, 50.9 ppm.

Exact Mass [M-H_2O+H]$^+$: 169.0420 (calculated), 169.0421 (found).

(*R*)-4-bromo-α-(bromomethyl)-2-hydroxybenzenemethanol (2o)

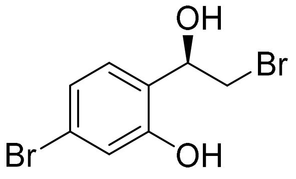

$C_8H_8Br_2O_2$: 295.958 g/mol

^{1}H NMR (600 MHz, $CDCl_3$) δ 7.81 (s, 1H), 7.15 – 6.55 (m, 3H), 5.11 – 4.86 (m, 1H), 3.65 (d, *J* = 17.8 Hz, 2H), 3.31 (s, 1H) ppm.

^{13}C NMR (151 MHz, $CDCl_3$) δ 156.2, 128.9, 123.5, 123.2, 122.0, 120.9, 74.8, 38.2 ppm.

Exact Mass [M-H_2O+H]$^+$: 278.8843 (calculated), 278.8828 (found).

Supplementary Tables

Supplementary Table 1. Pairwise identity of CbAR with selected 11 related proteins.

Protein	Organism	Accession No.	Amino acid	Identity to CbAR (%)
17β-HSDcl	*Curvularia lunata*	3IS3_A	270	86.6
ClaC	*Passalora fulva*	XP_047768478.1	267	84.2
ARti2	*Talaromyces islandicus*	CRG89873.1	265	83.9
CPUR 05429	*Claviceps purpurea*	M1W270.1	264	81.3
ARti	*Talaromyces islandicus*	CRG86682.1	265	79.2
MdpC	*Aspergillus nidulans*	XP_657750.1	265	74.6
AgnL6	*Paecilomyces divaricatus*	A0A411PQN6.1	268	68.9
T4HNR	*Pyricularia oryzae*	XP_003709023.1	283	63.3
T3HNR	*Colletotrichum orbiculare*	P87025.4	282	63.5
GDH	*Bacillus megaterium*	WP_013081865.1	261	30.2
CDH	*Rhodococcus erythropolis*	Q9RA05.1	277	27.4

Supplementary Table 2. X-ray data collection and refinement statistics.

	CbAR-NADP$^+$	CbAR-NADP$^+$-Emodin	CbAR-H162F-NADP$^+$-**1e**	CbAR-H162F-NADP$^+$-**1o**
**PDB code**	7YB1	7YB2	8HFJ	8HFK
**Data collection**				
Space group	$P\ 4_1\ 2_1\ 2$	$P\ 2\ 2_1\ 2_1$	$P\ 4_1\ 2_1\ 2$	$P\ 4_1\ 2_1\ 2$
Cell dimensions				
a, *b*, *c* (Å)	124.62, 124.62, 134.21	66.80, 123.36, 126.11	124.92, 124.92, 133.78	124.87, 124.87, 133.19
α, β, γ (°)	90, 90, 90	90, 90, 90	90, 90, 90	90, 90, 90
Resolution (Å)	23.66 - 3.30 (3.42 - 3.30)	45.89 - 1.85 (1.92 - 1.85)	24.06 – 2.75 (2.848 – 2.75)	24.04 – 2.90 (3.00 – 2.90)
R_{merge}	0.29 (0.48)	0.08 (0.61)	0.20 (0.58)	0.23 (0.77)
$I/\sigma\ (I)$	7.2 (4.3)	32.6 (3.3)	7.9 (2.7)	8.3 (2.6)
$CC_{1/2}$	0.912 (0.819)	0.996 (0.868)	0.983 (0.827)	0.987 (0.827)
Completeness (%)	99.5 (99.9)	98.74 (89.96)	99.69 (99.86)	99.67 (99.96)
Redundancy	10.4 (8.5)	12.1 (7.1)	6.8 (5.3)	9.8 (8.6)
**Refinement**				
No. reflections	16410 (1595)	88466 (7927)	28077 (2777)	23869 (2325)
R_{work}/R_{free}	0.206/0.285	0.151/0.188	0.205/0.270	0.202/0.289
No. atoms				
Protein	7525	7918	7669	7595
Ligand	192	296	224	240
Water	17	569	103	55
B-factors (Å^2)				
Protein	17.4	21.2	21.9	30.8
Ligand	13.4	20.8	22.0	31.8
Water	26.7	29.3	21.9	22.9
R.m.s. deviations				
Bond lengths (Å)	0.015	0.015	0.015	0.015
Bond angles (°)	1.94	1.85	1.93	1.87
Ramachandran outliers (%)	0.51	0.00	0.00	0.40
Ramachandran favored (%)	90.67	96.72	94.55	91.72

The values in parentheses are for highest-resolution shell.

Supplementary Table 3. Asymmetric reduction of various 2,2-disubstituted-1,3-cyclopentanediones by CbAR and CbAR-H162F variant.

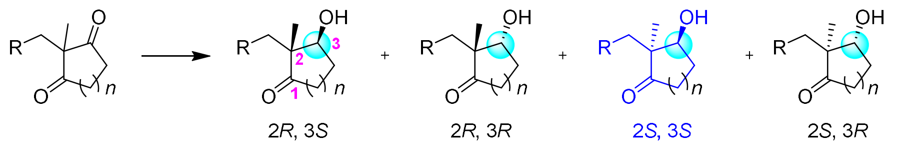

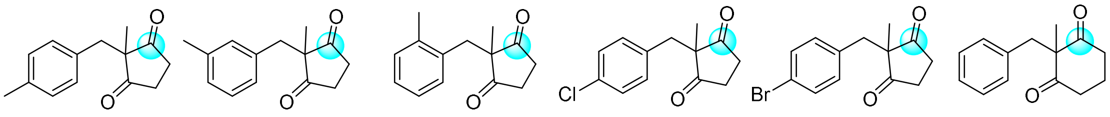

1e **1f** **1g** **1h** **1i** **1j**

Substrate	Enzymes	Conversion (%)	Stereoisomeric distribution of the ketol product (%)			
			(2*R*, 3*S*)	(2*R*, 3*R*)	(2*S*, 3*S*)	(2*S*, 3*R*)
**1e**	WT	46	-	-	>99	-
	H162F	99	-	-	>99	-
**1f**	WT	42	-	-	>99	-
	H162F	98	-	-	>99	-
**1g**	WT	29	-	-	>99	-
	H162F	93	-	-	>99	-
**1h**	WT	76	-	-	>99	-
	H162F	99	-	-	>99	-
**1i**	WT	70	-	-	>99	-
	H162F	97	-	-	>99	-
**1j**	WT	19	-	-	>99	-
	H162F	46	-	-	>99	-

Supplementary Table 4. Kinetic parameters of wild-type CbAR and its variants towards 2-methyl-2-(4-methylbenzyl) cyclopentane-1,3-dione (**1e**).

Enzyme	K_m (mM)	k_{cat} (min^{-1})	k_{cat} / K_m ($min^{-1}\cdot mM^{-1}$)	Stereoisomeric distribution of the ketol product (%)			
				(2*R*, 3*S*)	(2*R*, 3*R*)	(2*S*, 3*S*)	(2*S*, 3*R*)
WT	5.13 ± 0.33	0.58 ± 0.01	0.11 ± 0.01	-	-	>99	-
H162F	0.61 ± 0.06	3.00 ± 0.13	4.92 ± 0.29	-	-	>99	-
Y210A	2.25 ± 0.18	0.77 ± 0.06	0.35± 0.01	-	-	>99	-
Y210F	6.03 ± 0.16	3.07 ± 0.13	0.51 ± 0.02	-	-	>99	-

Supplementary Table 5. Reduction of various aryl ketones by CbAR and CbAR-H162F variant.

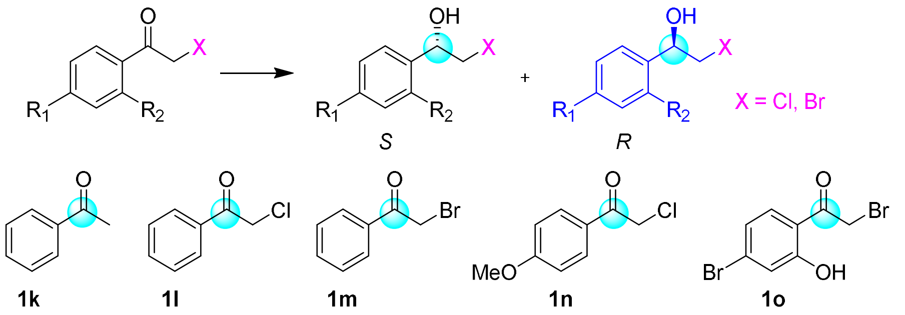

Substrate	Enzymes	Conversion (%)	Stereoisomeric distribution of the ketol product (%)	
			(*R*)	(*S*)
**1k**	WT	ND	-	-
	H162F	ND	-	-
**1l**	WT	ND	-	-
	H162F	62	>99	-
**1m**	WT	ND	-	-
	H162F	65	>99	-
**1n**	WT	ND	-	-
	H162F	13	>99	-
**1o**	WT	37	>99	-
	H162F	70	>99	-

Supplementary Table 6. Primers used in this study.

Name	Sequence (5'-3')
S151A-F	CATCG**GCG**AACACTTCCAGAGACTTCAGTGTC
S151A-R	GGAAGTGTT**CGC**CGATGTCATGATTATTCGGCCC
S151T-F	CATCG**ACA**AACACTTCCAGAGACTTCAGTGTC
S151T-R	GGAAGTGTT**TGT**CGATGTCATGATTATTCGGCCC
N152I-F	ATCGTCC**ATT**ACTTCCAGAGACTTCAGTGTCC
N152I-R	TGGAAGT**AAT**GGACGATGTCATGATTATTCGGC
H162A-F	CCAAG**GCT**TCGCTATATTCCGGGTCAAAGG
H162A-R	ATATAGCGA**AGC**CTTGGGGACACTGAAGTCTC
H162N-F	CCAAG**AAC**TCGCTATATTCCGGGTCAAAGG
H162N-R	ATATAGCGA**GTT**CTTGGGGACACTGAAGTCTC
H162F-F	CCAAG**TTC**TCGCTATATTCCGGGTCAAAGG
H162F-R	ATATAGCGA**GAA**CTTGGGGACACTGAAGTCTC
K169A-F	CGGGTCA**GCA**GGCGCCATCGACAGC
K169A-R	TGGCGCC**TGC**TGACCCGGAATATAGCGAGTG
K169L-F	CGGGTCA**CTG**GGCGCCATCGACAGC
K169L-R	TGGCGCC**CAG**TGACCCGGAATATAGCGAGTG
H209A-F	CACAG**GCA**TACATTCCCAACGGAGAAACATATACACC
H209A-R	GGGAATGTA**TGC**CTGTGAGACATCGTGGAACATGTC
Y210A-F	ACAGCAC**GCT**ATTCCCAACGGAGAAACATATACACC
Y210A-R	TGGGAAT**AGC**GTGCTGTGAGACATCGTGGAAC
Y210F-F	ACAGCAC**TTC**ATTCCCAACGGAGAAACATATACACC
Y210F-R	TGGGAAT**GAA**GTGCTGTGAGACATCGTGGAAC
I211A-F	ACTAC**GCA**CCCAACGGAGAAACATATACACC
I211A-R	TCCGTTGGG**TGC**GTAGTGCTGTGAGACATCGTGG
P212G-F	GCACTACATT**GGA**AACGGAGAAACATATACACCAGAGG
P212G-R	CGTT**TCC**AATGTAGTGCTGTGAGACATCGTG
N213A-F	TTCCC**GCA**GGAGAAACATATACACCAGAGGAACG
N213A-R	TGTTTCTCC**TGC**GGGAATGTAGTGCTGTGAGACATC
E215A-F	ACGGA**GCA**ACATATACACCAGAGGAACGCC
E215A-R	TGTATATGT**TGC**TCCGTTGGGAATGTAGTGCTGTG
T216A-F	GAGAA**GCA**TATACACCAGAGGAACGCCAGAAG
T216A-R	TGGTGTATA**TGC**TTCTCCGTTGGGAATGTAGTGC
P219G-F	ATACA**GGA**GAGGAACGCCAGAAGATGGC
P219G-R	CGTTCCTC**TCC**TGTATATGTTTCTCCGTTGGGAATGTAGTG
P212G/P219G-F	ATACA**GGA**GAGGAACGCCAGAAGATGGC
P212G/P219G-R	CGTTCCTC**TCC**TGTATATGTTTCTCCGTT**TCC**AATGTAGTG

Supplementary Table 7. HPLC chromatographic conditions for enzymatic activity assays.

Substrate	Column	Mobile phase	Test conditions
**1a**	ZORBAX SB-C18	65:35 acetonitrile(0.1% formic acid):ddH_2O(0.1% formic acid)	UV 254 nm, 25 ℃, 1 mL/min
**1b**	ZORBAX SB-C18	50:50 acetonitrile(0.1% formic acid):ddH_2O(0.1% formic acid)	UV 270 nm, 25 ℃, 1 mL/min
**1c**	ZORBAX SB-C18	65:35 acetonitrile(0.1% formic acid):ddH_2O(0.1% formic acid)	UV 270 nm, 25 ℃, 1 mL/min
**1d**	ZORBAX SB-C18	65:35 acetonitrile(0.1% formic acid):ddH_2O(0.1% formic acid)	UV 270 nm, 25 ℃, 1 mL/min
**1e**	ZORBAX SB-C18	50:50 acetonitrile(0.1% formic acid):ddH_2O(0.1% formic acid)	UV 210 nm, 25 ℃, 1 mL/min
**1f**	ZORBAX SB-C18	50:50 acetonitrile(0.1% formic acid):ddH_2O(0.1% formic acid)	UV 210 nm, 25 ℃, 1 mL/min
**1g**	ACQUITY UPLC BEH C18	50:50 acetonitrile(0.1% formic acid):ddH_2O(0.1% formic acid)	UV 210 nm, 25 ℃, 0.3 mL/min
**1h**	ZORBAX SB-C18	50:50 acetonitrile(0.1% formic acid):ddH_2O(0.1% formic acid)	UV 210 nm, 25 ℃, 1 mL/min
**1i**	ACQUITY UPLC BEH C18	50:50 acetonitrile(0.1% formic acid):ddH_2O(0.1% formic acid)	UV 210 nm, 25 ℃, 0.3 mL/min
**1j**	CORTECS UPLC C18	40:60 methano(0.1% TFA):ddH_2O(0.1% TFA)	UV 210 nm, 25 ℃, 0.3 mL/min
**1k**	CORTECS UPLC C18	5% methanol for 3 min and linear gradient 5-100% methanol over 7 min	UV 254 nm, 25 ℃, 0.3 mL/min
**1l**	CORTECS UPLC C18	5% methanol for 3 min and linear gradient 5-100% methanol over 7 min	UV 210 nm, 25 ℃, 0.3 mL/min
**1m**	CORTECS UPLC C18	5% methanol for 3 min and linear gradient 5-100% methanol over 7 min	UV 210 nm, 25 ℃, 0.3 mL/min
**1n**	CORTECS UPLC C18	5% methanol for 3 min and linear gradient 5-100% methanol over 7 min	UV 210 nm, 25 ℃, 0.3 mL/min
**1o**	CORTECS UPLC C18	5% methanol for 3 min and linear gradient 5-100% methanol over 7 min	UV 210 nm, 25 ℃, 0.3 mL/min

Supplementary Table 8. Chiral HPLC chromatographic conditions for the enantioselectivity analysis.

Substrate	Column	Mobile phase	Test conditions
**1a**	Chiralcel OD-H	95:5 n-Hex:i-PrOH	UV 254nm, 25°C, 1.0 mL/min
**1e**	Chiralcel OD-H	98:2 n-Hex:i-PrOH	UV 210nm, 25°C, 0.8 mL/min
**1f**	Chiralcel OD-H	98:2 n-Hex:i-PrOH	UV 210nm, 25°C, 0.8 mL/min
**1g**	Chiralcel OD-H	97:3 n-Hex:i-PrOH	UV 210nm, 25°C, 0.8 mL/min
**1h**	Chiralcel OD-H	99:1 n-Hex:i-PrOH	UV 210nm, 25°C, 1.0 mL/min
**1i**	Chiralcel OD-H	99:1 n-Hex:i-PrOH	UV 210nm, 25°C, 1.0 mL/min
**1j**	CHIRALPAK IB-3	96:4 n-Hex:i-PrOH	UV 210nm, 25°C, 0.8 mL/min
**1m**	CHIRALPAK IB-3	98:2 n-Hex:i-PrOH	UV 210nm, 25°C, 0.8 mL/min
**1n**	CHIRALPAK IB-3	97:3 n-Hex:i-PrOH	UV 210nm, 25°C, 0.8 mL/min
**1o**	CHIRALPAK IB-3	97:3 n-Hex:i-PrOH	UV 210nm, 25°C, 0.8 mL/min

Supplementary Table 9. Chiral GC conditions for the enantioselectivity analysis.

Substrate	Program [a]	Retention time (min)	
**1l**	80/0/10/160/2/2/170/2/1/180/4	20.42 (*S*)	20.73 (*R*)

[a] GC program: initial temp. (ºC) / time (min) / ramp (ºC/min) / temp. (ºC) / time (min) / ramp (ºC/min) / temp. (ºC) / time (min) / ramp (ºC/min) / final temp. (ºC) / time (min).

Supplementary Figures

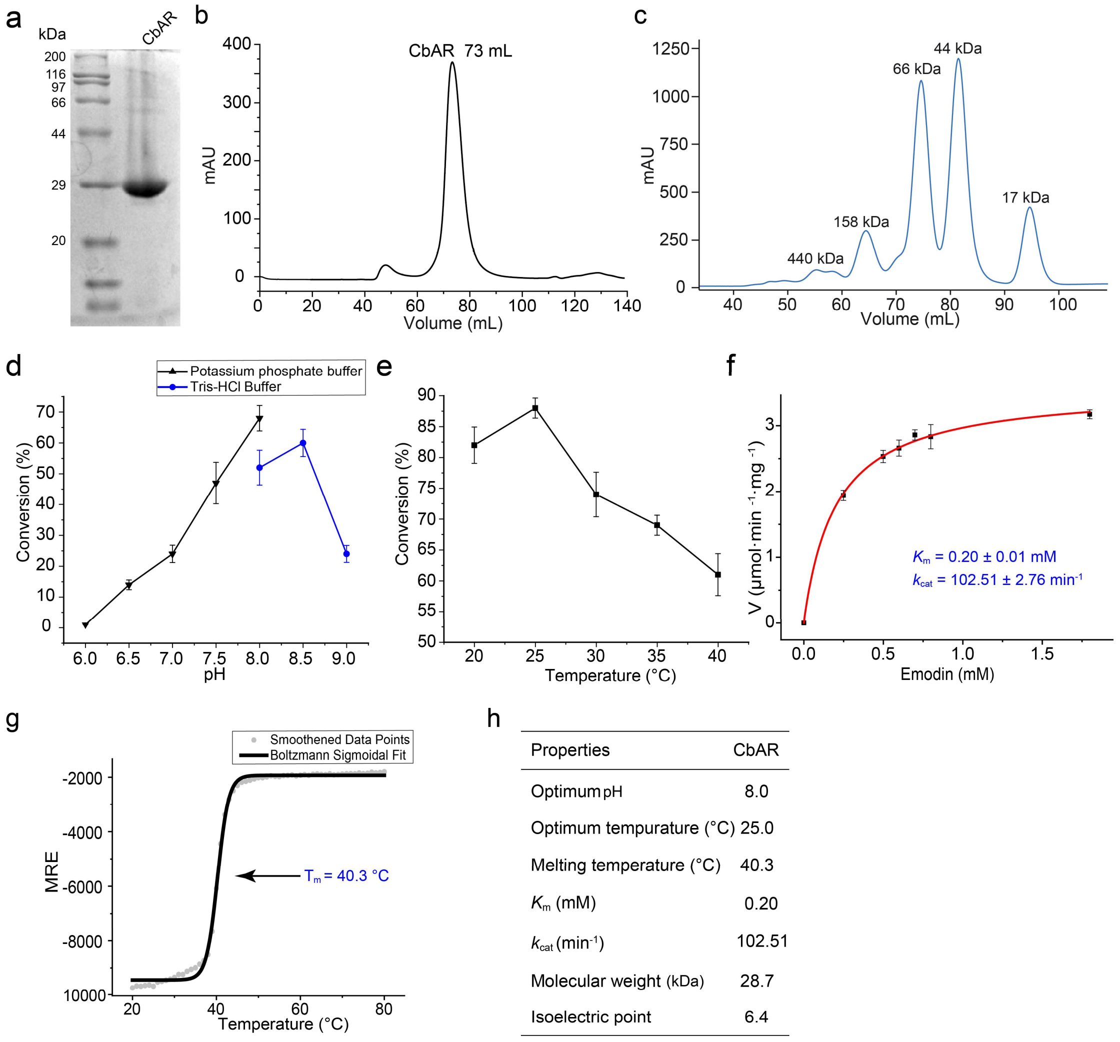

Properties	CbAR
Optimum pH	8.0
Optimum tempurature (°C)	25.0
Melting temperature (°C)	40.3
K_m (mM)	0.20
k_{cat} (min^{-1})	102.51
Molecular weight (kDa)	28.7
Isoelectric point	6.4

Supplementary Figure 1. Characterization of the anthrol reductase CbAR. (a) Purification of recombinant CbAR. The experiment was repeated independently three times with similar results. (b) Size-exclusion chromatography (SEC) analysis of CbAR (Mw. 28.7 kDa). The concentrated CbAR protein (5 mg/mL) was eluted as a single peak 73 mL when it was uploaded onto a HiLoad 16/600 Superdex 200 pg gel-filtration column. (c) Standard markers were eluted at different volume from a HiLoad 16/600 Superdex 200 pg gel-filtration column. (1. Myoglobulln, 1.5 mg/mL, Mr 17 kDa. 2. Ovalbumln, 5 mg/mL, Mr 44 kDa. 3. Albumln, 5 mg/mL, Mr 66 kDa. 4. IgG, 0.2 mg/mL, Mr 158 kDa. 5. Ferrltln, 0.24 mg/mL, Mr 440 kDa.) (d) Optimum pH (the average of n = 3 independent experiments). Error bars indicate ±sd. (e) Optimum temperature (the average of n = 3 independent experiments). Error bars indicate ±sd. (f) The kinetic plots of CbAR (the average of

n = 3 independent experiments). Error bars indicate ±sd. (g) Plots of circular dichroism of CbAR versus different temperatures at 220 nm. (h) Enzymatic properties of purified CbAR.

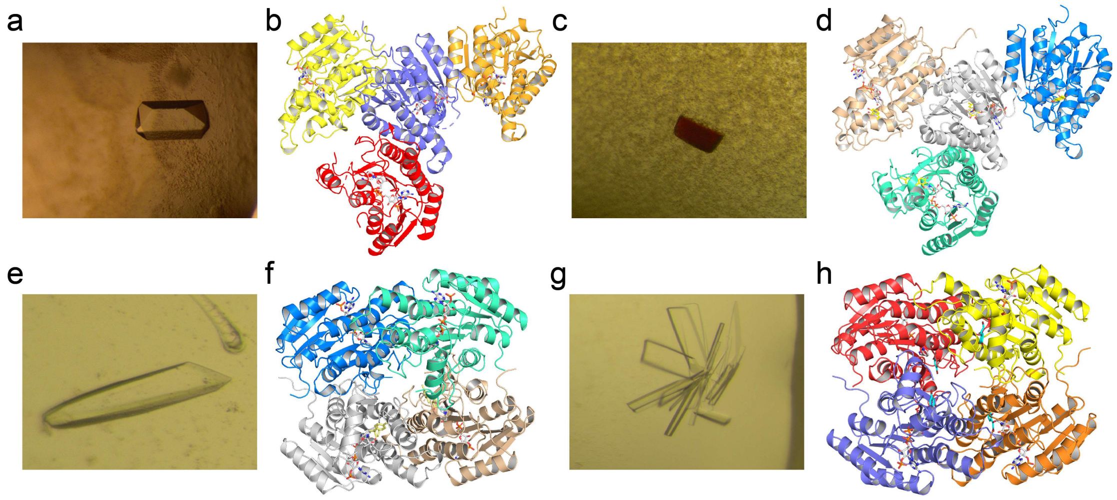

Supplementary Figure 2. Protein crystals and structures of CbAR and CbAR-H162F in complex with substrates. (a) Protein crystals of CbAR-$NADP^+$ complex. (b) Crystal structures of CbAR-$NADP^+$ complex. Each asymmetric unit contains four monomers indicated by different colors. (c) Protein crystals of CbAR-$NADP^+$-Emodin complex. (d) Crystal structures of CbAR-$NADP^+$-Emodin complex. Each asymmetric unit contains four monomers indicated by different colors. (e) Protein crystals of CbAR-H162F-**1e** complex. (f) Crystal structures of CbAR-H162F-**1e** complex. Each asymmetric unit contains four monomers indicated by different colors. (g) Protein crystals of CbAR-H162F-**1o** complex. (h) Crystal structures of CbAR-H162F-**1o** complex. Each asymmetric unit contains four monomers indicated by different colors.

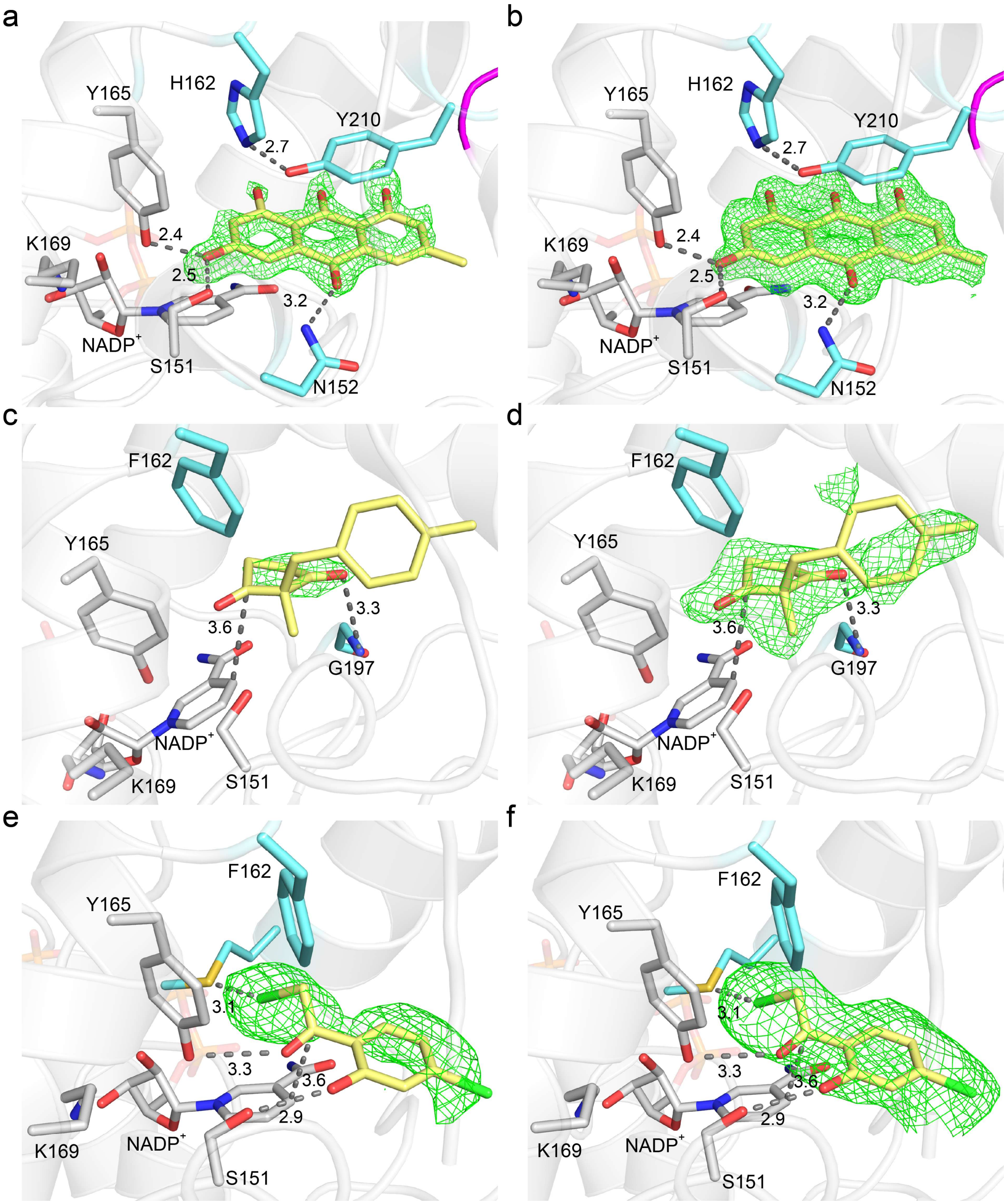

Supplementary Figure 3. The difference density maps and polder maps of substrates in different crystal structures. (a) The Fo – Fc omit map of emodin was contoured at 3.0 σ in green color. (b) The polder map of emodin was contoured at 3.0 σ in green color. (c) The Fo – Fc omit map of substrate **1e** was contoured at 3.0 σ in green color. (d) The polder map of substrate **1e** was contoured at 3.0 σ in green color. (e) The Fo – Fc omit map of substrate **1o** was contoured at 3.0 σ in green color. (f) The polder map of substrate **1o** was contoured at 3.0 σ in green color. $NADP^+$, substrates, the catalytic triad (Ser151, Tyr165 and Lys169), and residues involved in binding of substrates are highlighted in different colors.

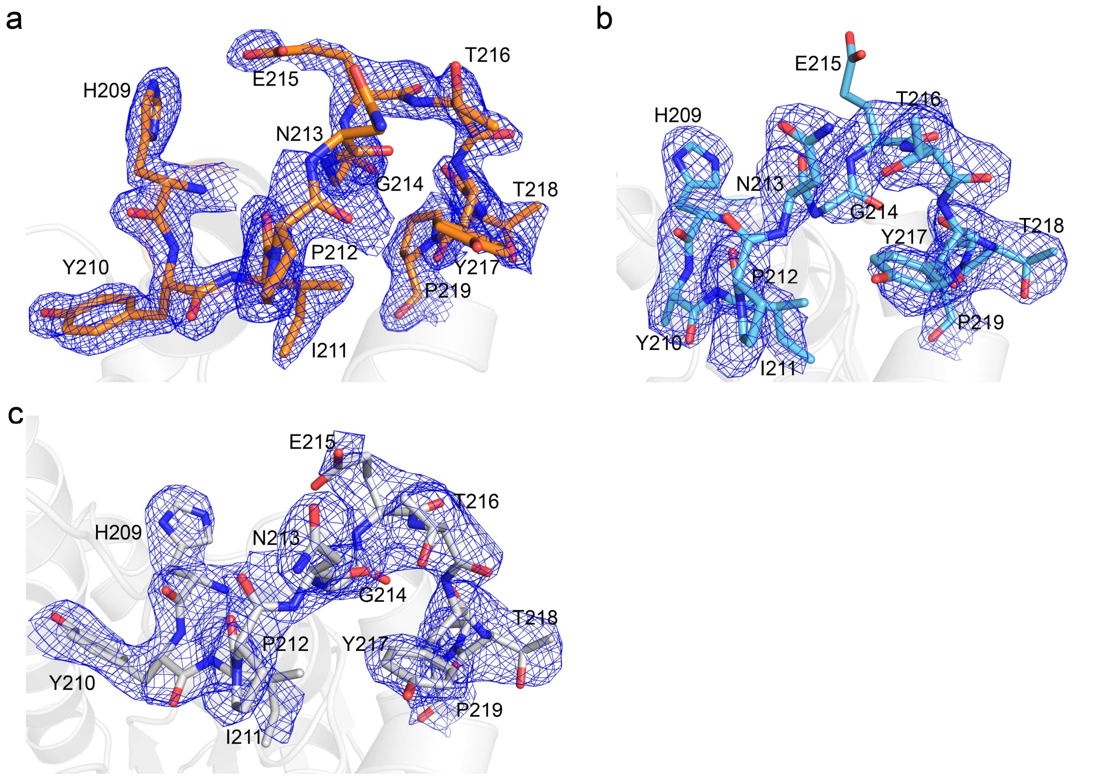

Supplementary Figure 4. The electron density map around the flexible region (between His209 and Pro219) in the different crystal forms. (a) The residues and 2Fo – Fc electron density map contoured at 1.0 σ around the flexible region in the structure of CbAR-NADP$^+$-Emodin complex are shown. (b) The residues and 2Fo – Fc electron density map contoured at 1.0 σ around the flexible region in the structure of CbAR-H162F-NADP$^+$-**1e** complex are shown. (c) The residues and 2Fo – Fc electron density map contoured at 1.0 σ around the flexible region in the structure of CbAR-H162F-NADP$^+$-**1o** complex are shown.

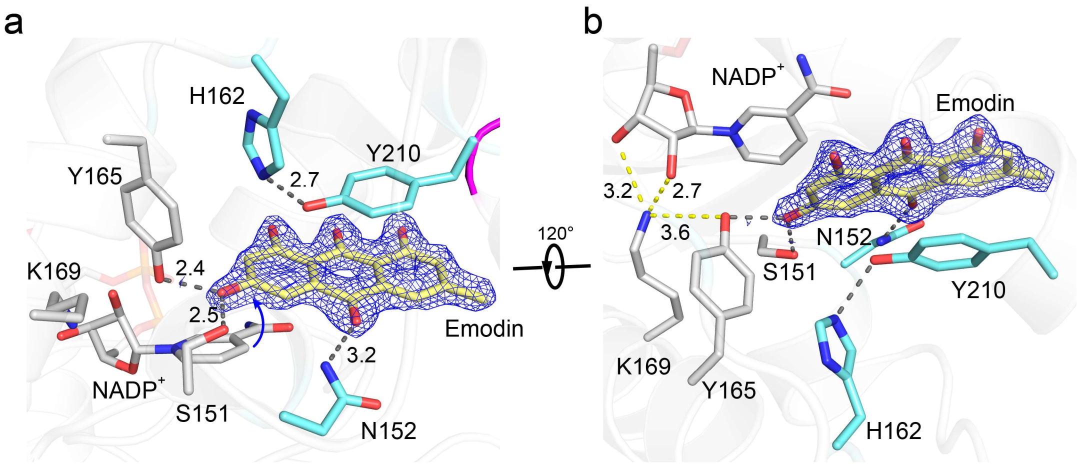

Supplementary Figure 5. Close-up views of substrate binding pocket of CbAR for emodin. (a) $NADP^+$, the catalytic triad (Ser151, Tyr165 and Lys169) and residues involved in binding of emodin (N152, H162 and Y210) are highlighted in different colors. The 2Fo – Fc electron density map of emodin was contoured at 1.0 σ in blue color. The proposed proton shuttling mechanism of CbAR towards the substrate is indicated by the blue arrow. (b) Close-up views of a 120° rotation along the X-axis of (a). The distance between Lys169 and $NADP^+$ or Tyr165 were indicated in yellow color.

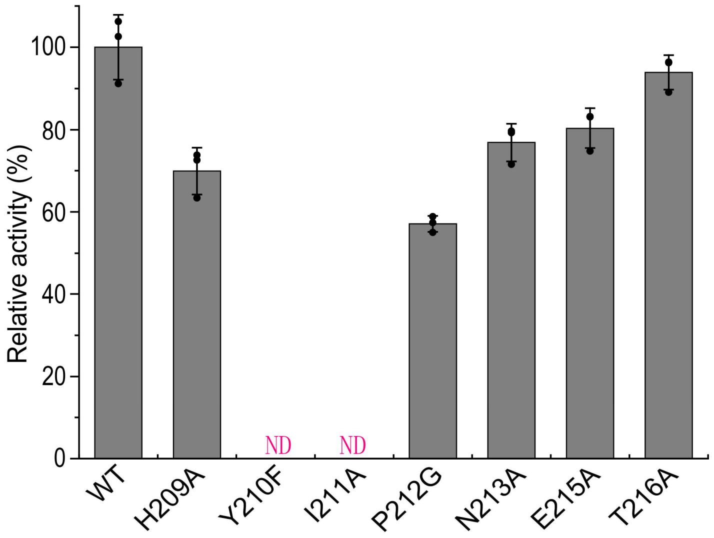

Supplementary Figure 6. The relative activity of wild-type (WT) CbAR and its variants towards emodin (expressed as the average of n = 3 independent experiments). ND, not detected. Error bars indicate ±sd. G214 was not mutanted in this study owing to the small side chain similar to alanine.

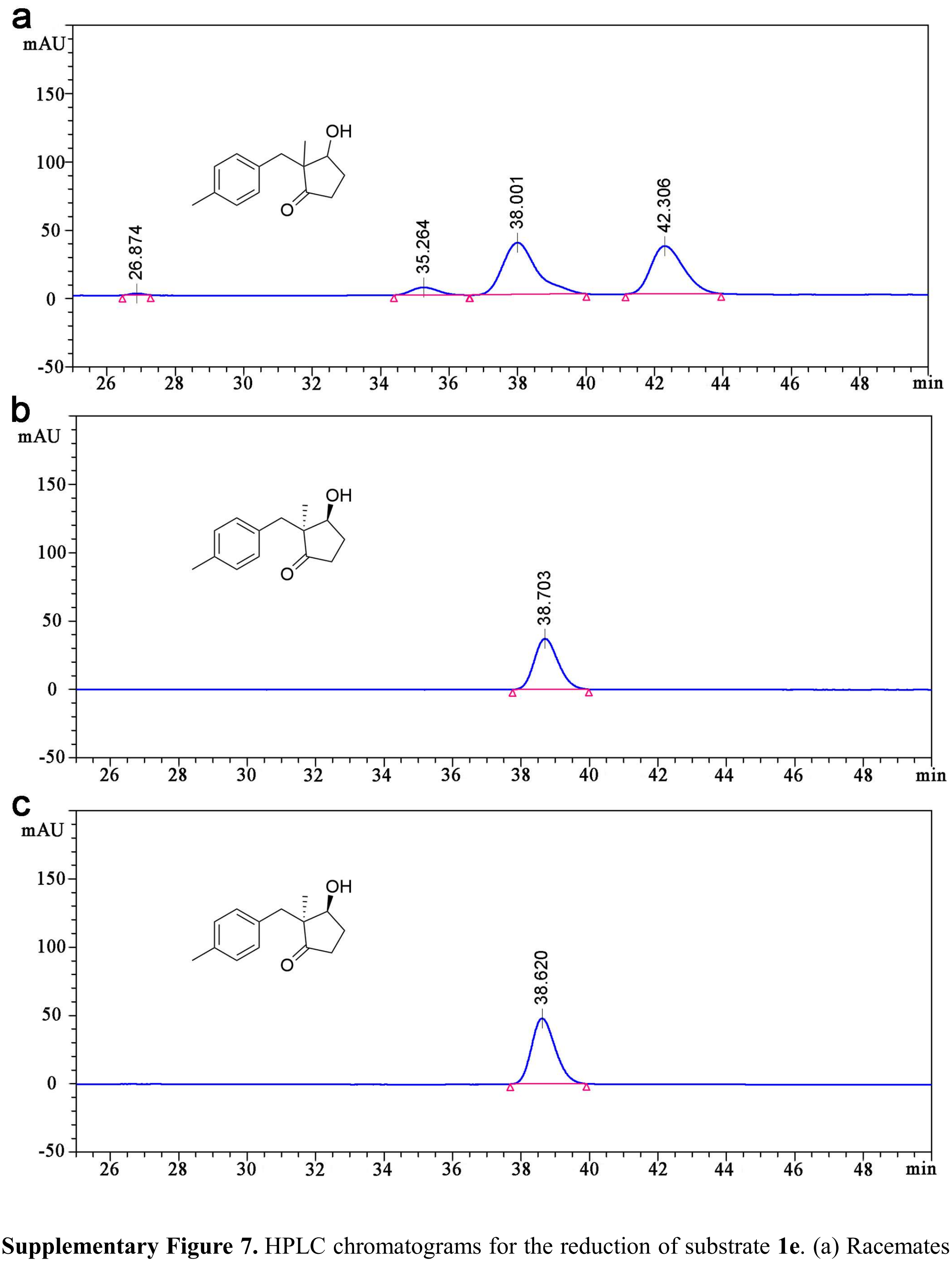

Supplementary Figure 7. HPLC chromatograms for the reduction of substrate **1e**. (a) Racemates made by reduction of **1e** with $NaBH_4$. (b) (2*S*, 3*S*)–**2e** prepared by WT-CbAR. (c) (2*S*, 3*S*)–**2e** prepared by CbAR-Y210A variant.

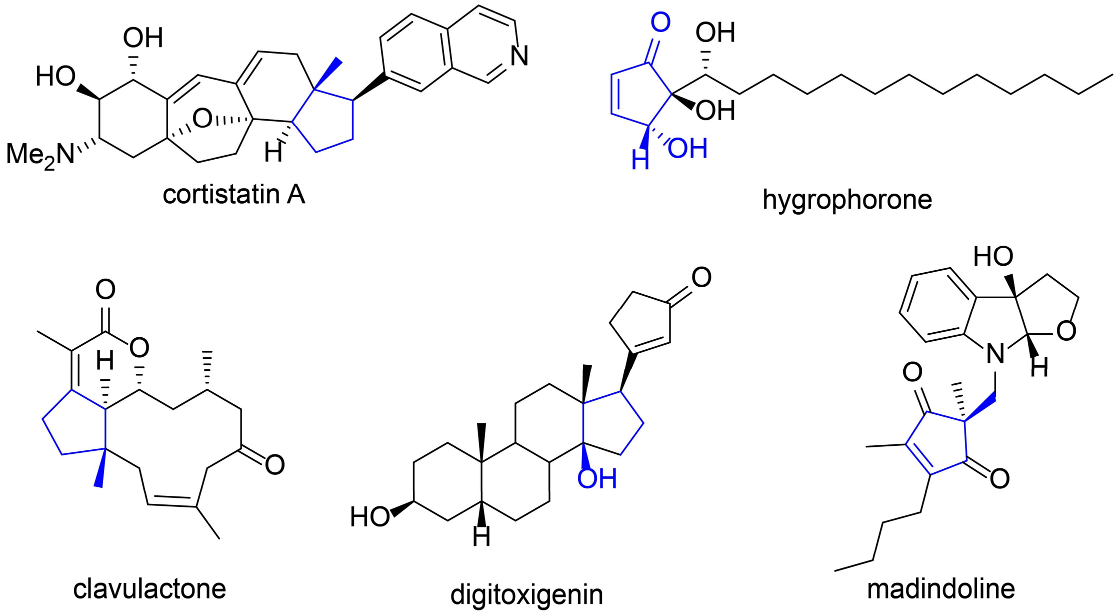

Supplementary Figure 8. Representative natural products containing 2,2-disubstituted-3-hydroxycycloketones moiety. 2,2-disubstituted-3-hydroxycycloketones moiety is highlighted in blue color.

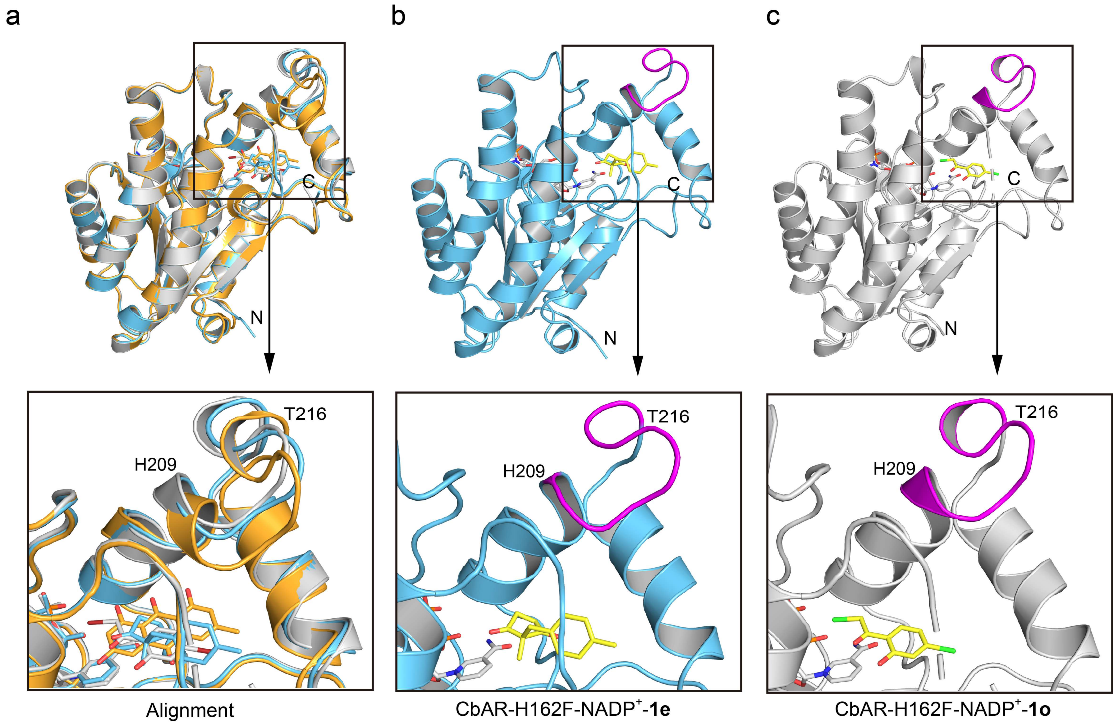

Supplementary Figure 9. Overall crystal structure of CbAR-H162F variant in complex with NADP$^+$-**1e** or NADP$^+$-**1o**. (a) The structural alignment of the CbAR-NADP$^+$-emodin (colored orange), CbAR-H162F-**1e** (colored cyan) and CbAR-H162F-**1o** (colored gray). (b) Crystal structure of CbAR-H162F-**1e** complex and close-up view of the region from residue 209 to 216. The region from residue 209 to 216 is shown in violet. (c) Crystal structure of CbAR-H162F-**1o** complex and close-up views of the region from residue 209 to 216. The region from residue 209 to 216 is shown in violet.

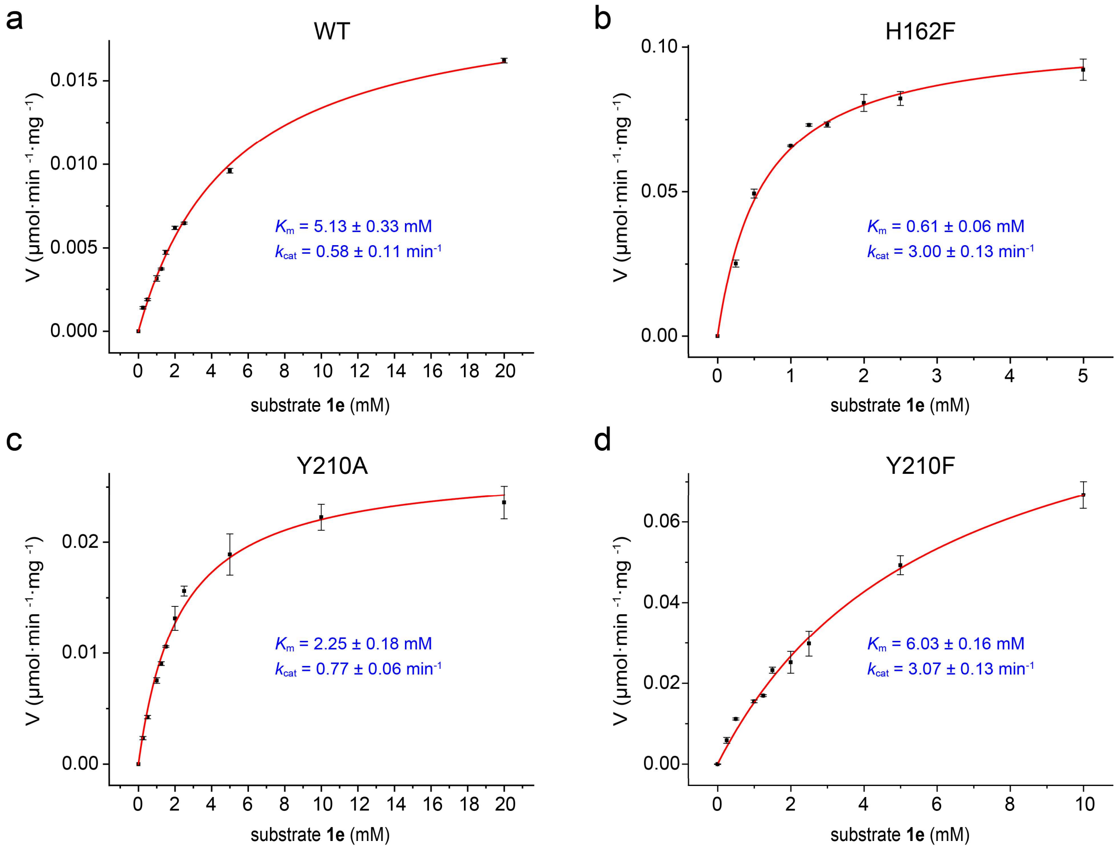

Supplementary Figure 10. The kinetic plots of CbAR and its variants towards substrate **1e.** (a) The kinetic plots of wild-type CbAR. (b) The kinetic plots of CbAR-H162F variant. (c) The kinetic plots of CbAR-Y210A variant. (d) The kinetic plots of CbAR-Y210F variant. All above results are expressed as the average of n = 3 independent experiments and error bars indicate ±sd.

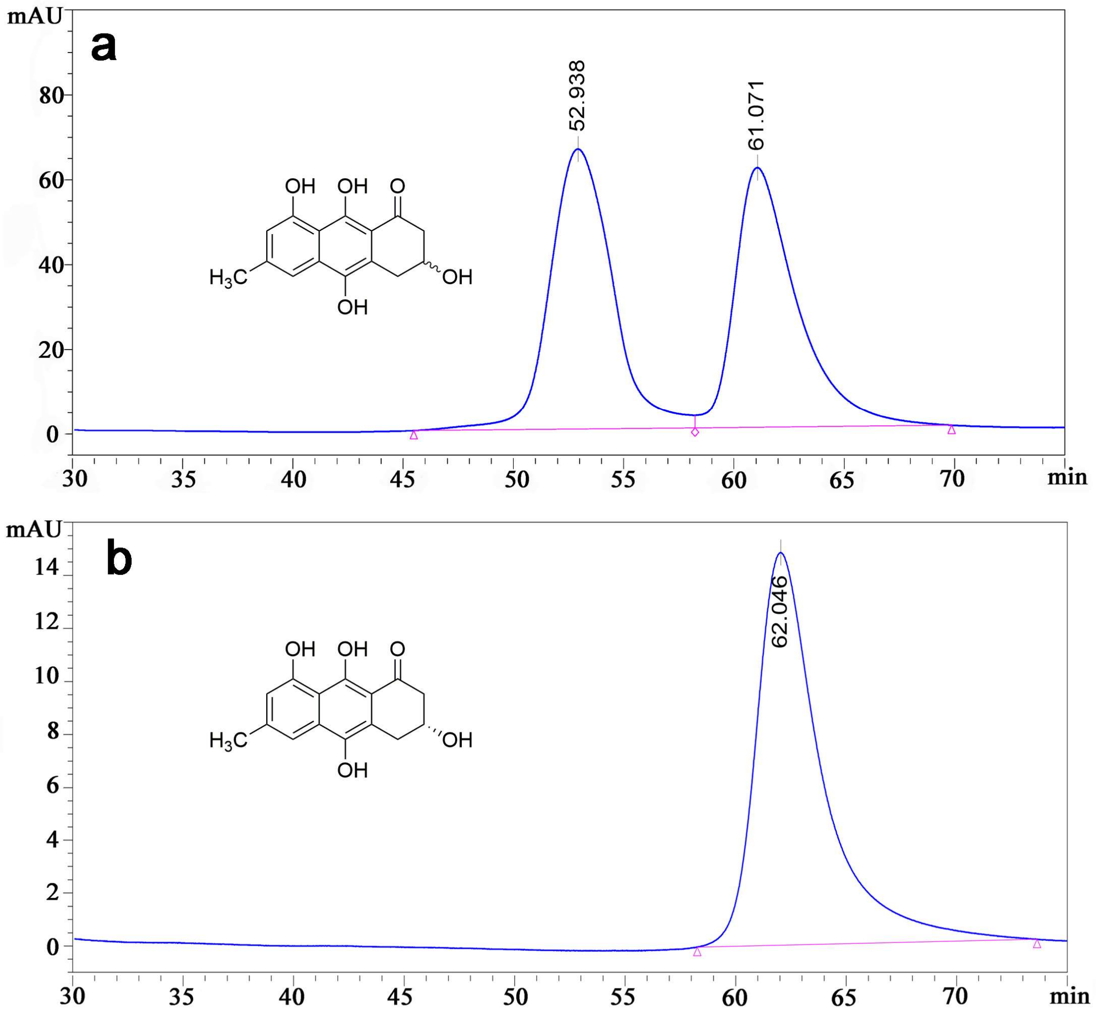

Supplementary Figure 11. HPLC chromatograms for the reduction of substrate **1a**. (a) Racemates made by reduction of **1a** with $NaBH_4$. (b) (*R*)-**2a** prepared by CbAR.

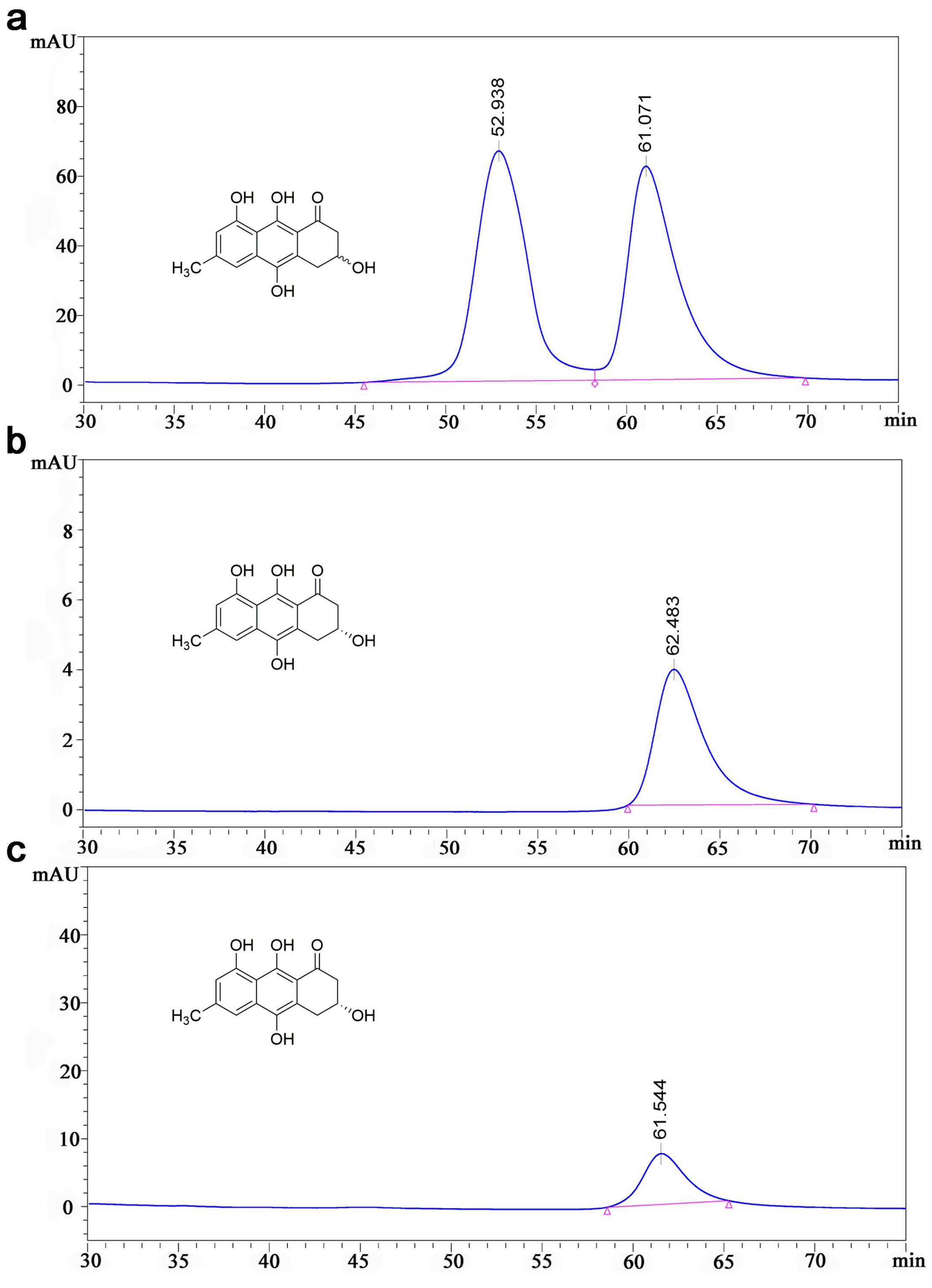

Supplementary Figure 12. HPLC chromatograms for the reduction of substrate **1a**. (a) Racemates made by reduction of **1a** with $NaBH_4$. (b) (*R*)-**2a** prepared by CbAR-P212G variant. (c) (*R*)-**2a** prepared by CbAR-P219G variant.

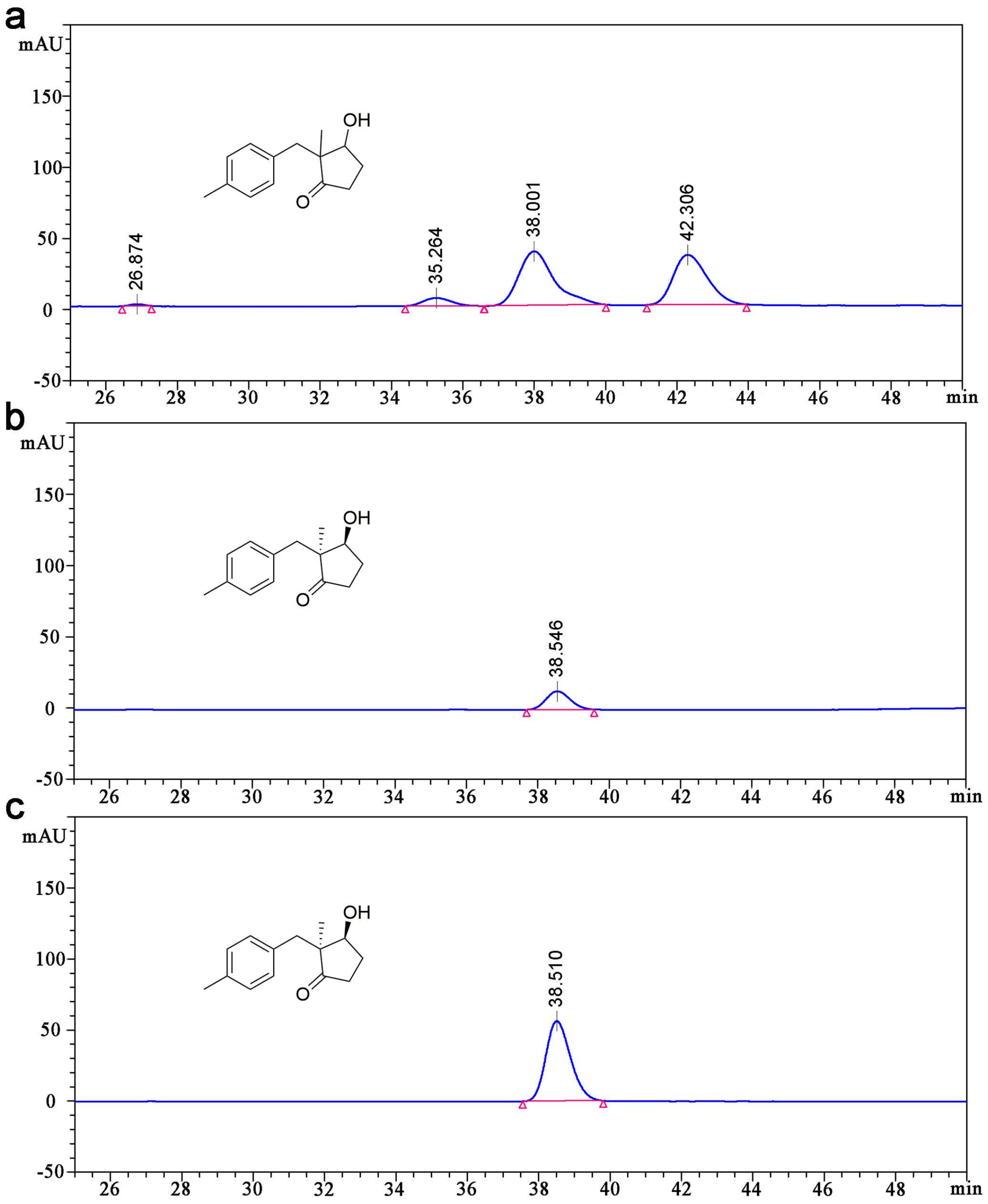

Supplementary Figure 13. HPLC chromatograms for the reduction of substrate **1e**. (a) Racemates made by reduction of **1e** with $NaBH_4$. (b) (2*S*, 3*S*)-**2e** prepared by CbAR-H162F variant. (c) (2*S*, 3*S*)-**2e** prepared by CbAR-Y210F variant.

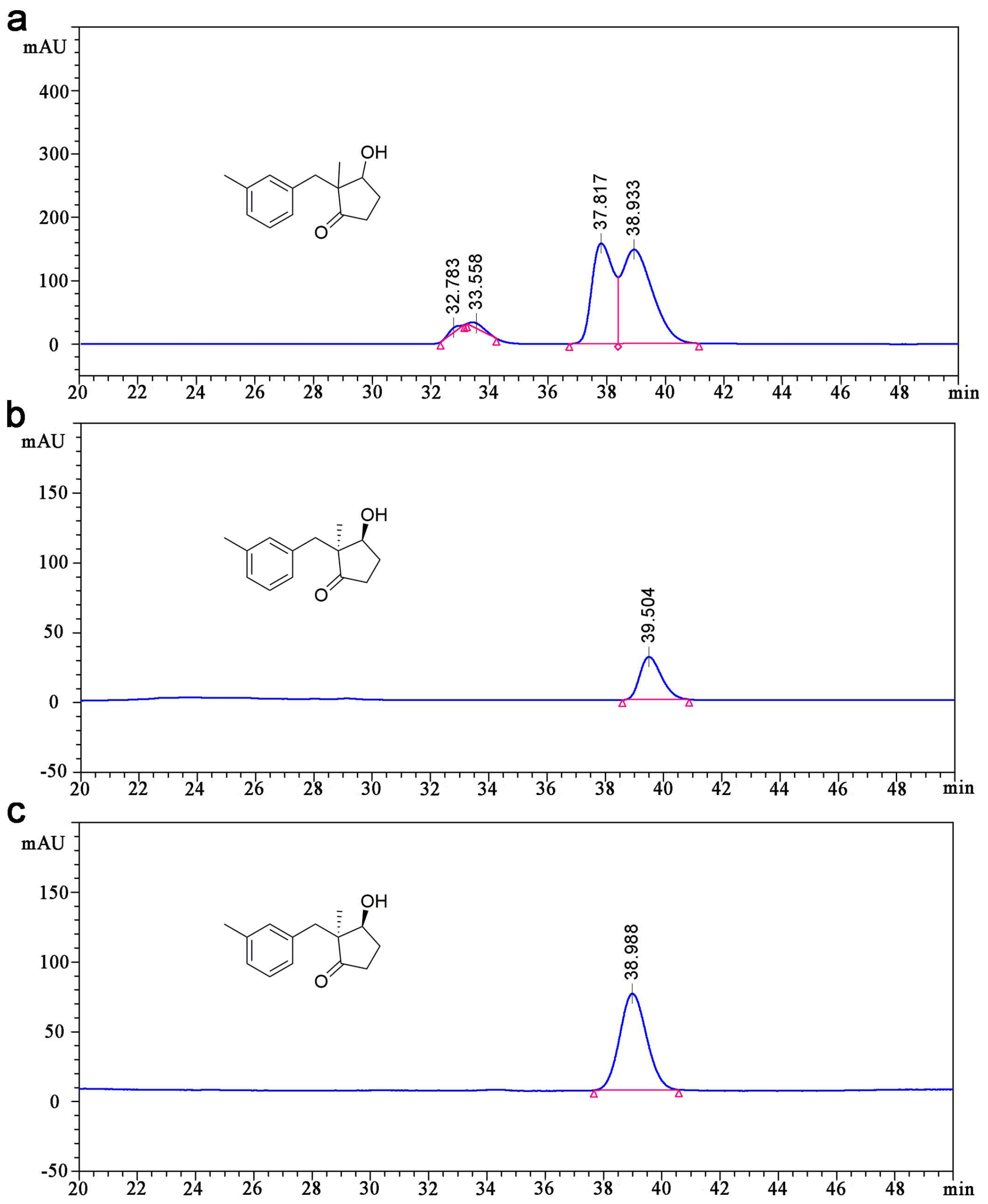

Supplementary Figure 14. HPLC chromatograms for the reduction of substrate **1f**. (a) Racemates made by reduction of **1f** with $NaBH_4$. (b) (2*S*, 3*S*)-**2f** prepared by CbAR. (c) (2*S*, 3*S*)-**2f** prepared by CbAR-H162F variant.

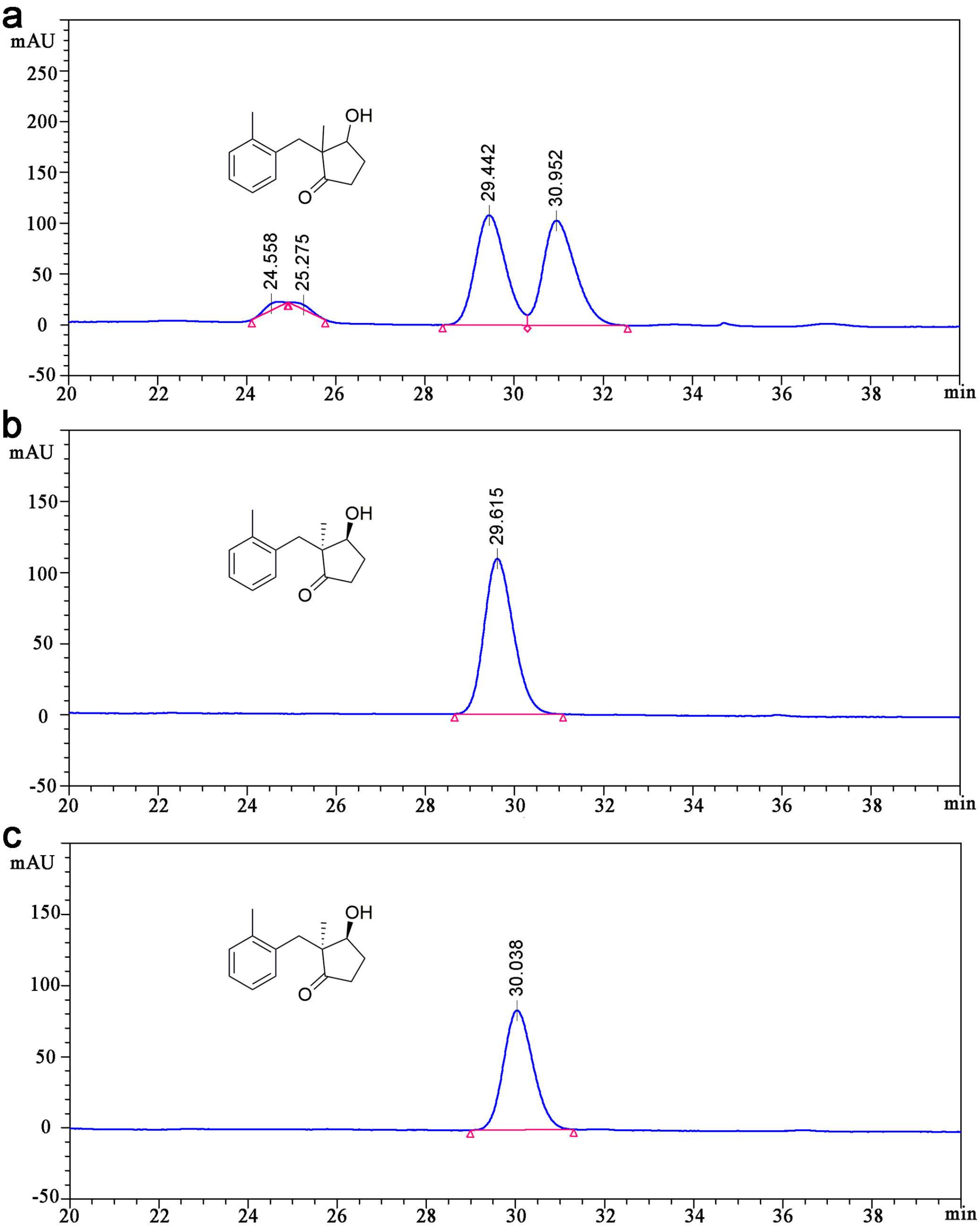

Supplementary Figure 15. HPLC chromatograms for the reduction of substrate **1g**. (a) Racemates made by reduction of **1g** with $NaBH_4$. (b) (2*S*, 3*S*)-**2g** prepared by CbAR-H162F variant. (c) (2*S*, 3*S*)-**2g** prepared by CbAR.

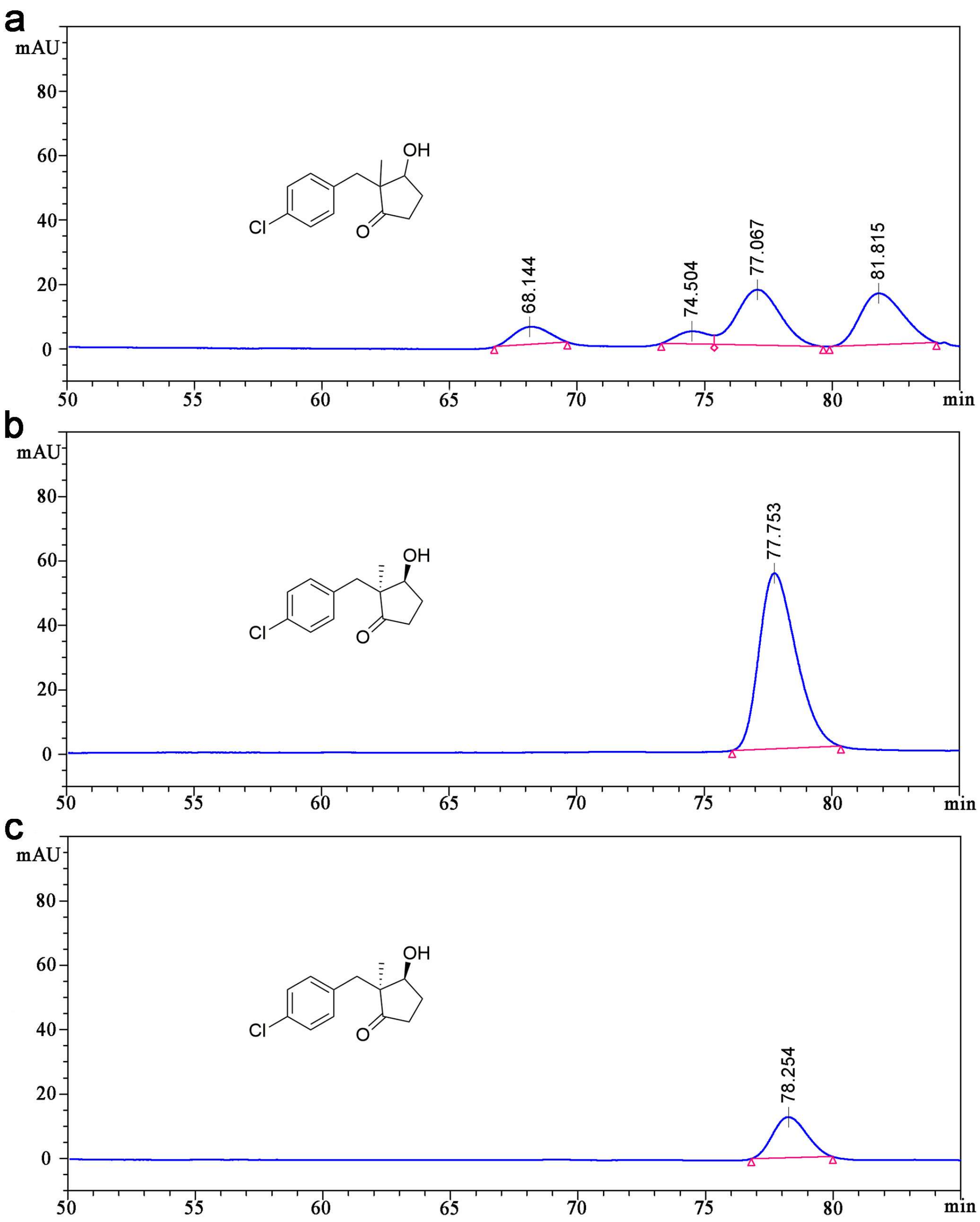

Supplementary Figure 16. HPLC chromatograms for the reduction of substrate **1h**. (a) Racemates made by reduction of **1h** with $NaBH_4$. (b) (2*S*, 3*S*)-**2h** prepared by CbAR-H162F variant. (c) (2*S*, 3*S*)-**2h** prepared by CbAR.

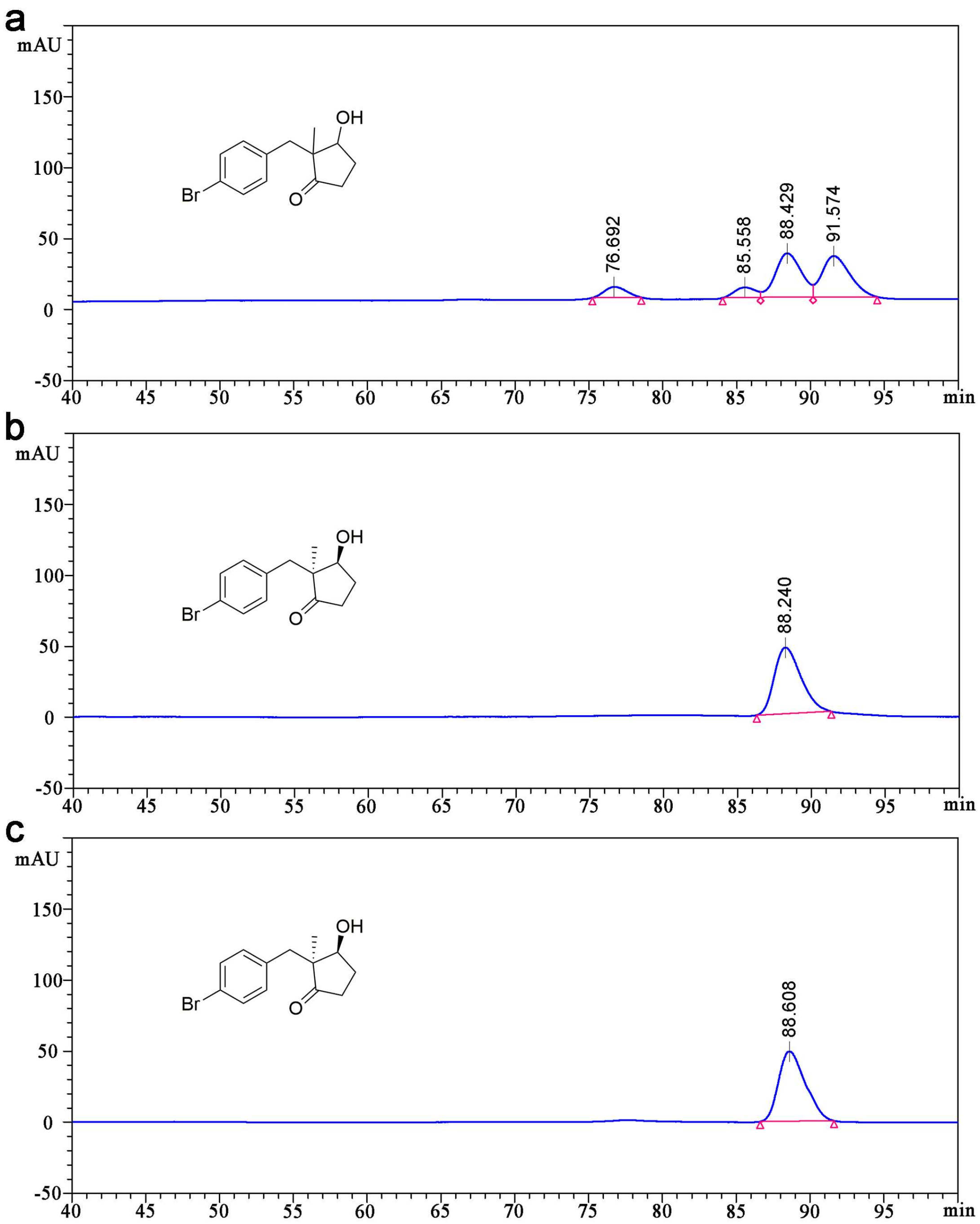

Supplementary Figure 17. HPLC chromatograms for the reduction of substrate **1i**. (a) Racemates made by reduction of **1i** with $NaBH_4$. (b) (2*S*, 3*S*)-**2i** prepared by CbAR-H162F variant. (c) (2*S*, 3*S*)-**2i** prepared by CbAR.

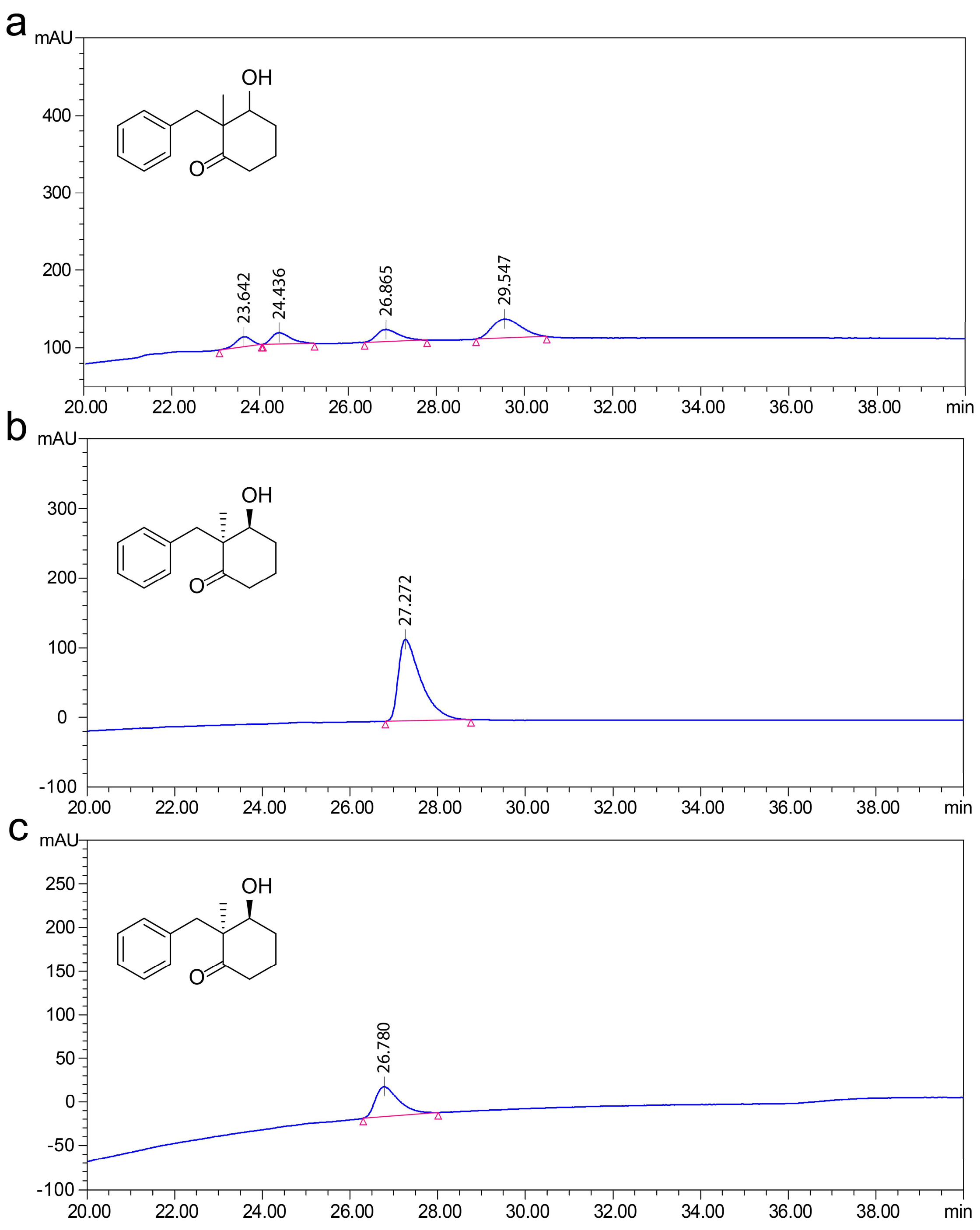

Supplementary Figure 18. HPLC chromatograms for the reduction of substrate **1j**. (a) Racemates made by reduction of **1j** with $NaBH_4$. (b) (2*S*, 3*S*)-**2j** prepared by CbAR-H162F variant. (c) (2*S*, 3*S*)-**2j** prepared by CbAR.

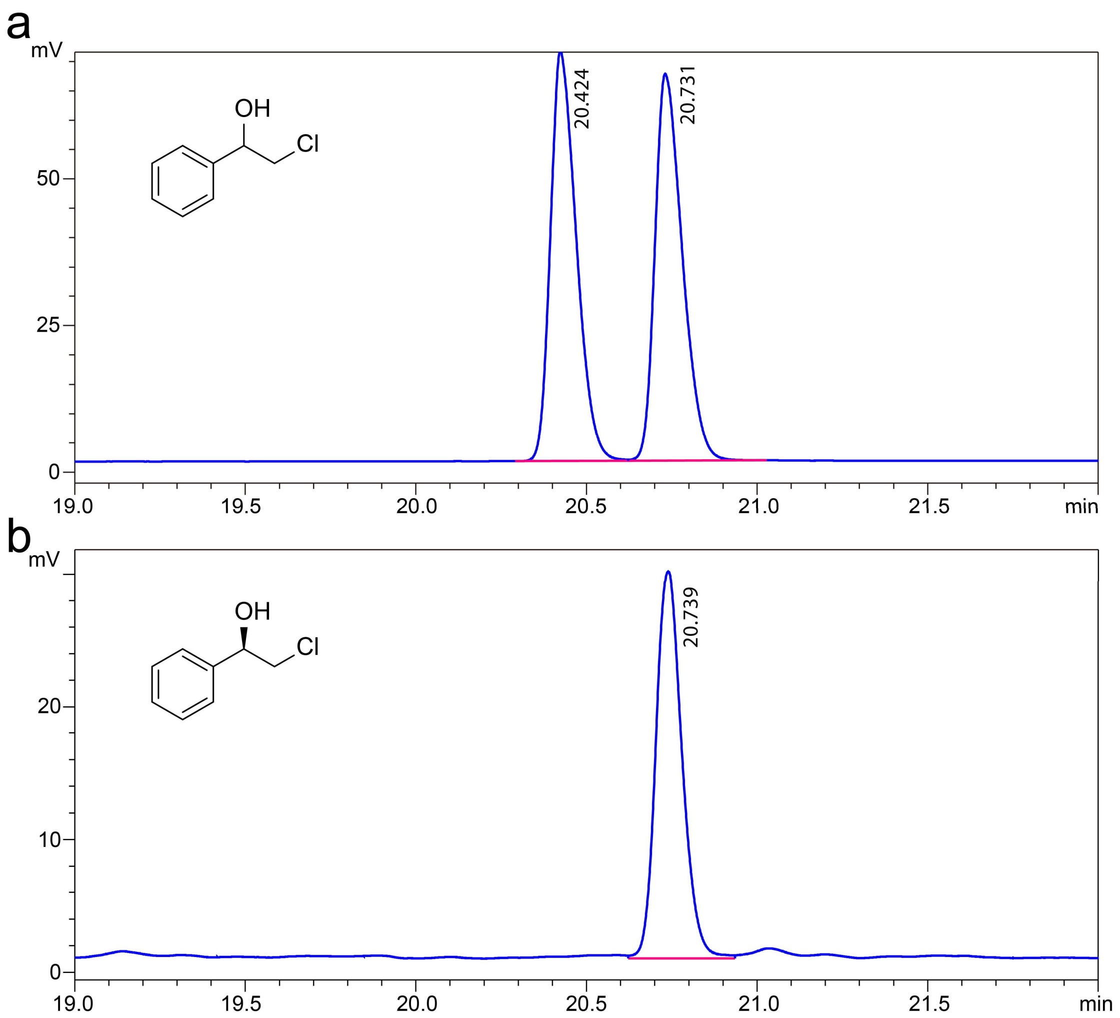

Supplementary Figure 19. GC chromatograms for the reduction of substrate **1l**. (a) Racemates made by reduction of **1l** with $NaBH_4$. (b) (*R*)-**2l** prepared by CbAR-H162F variant.

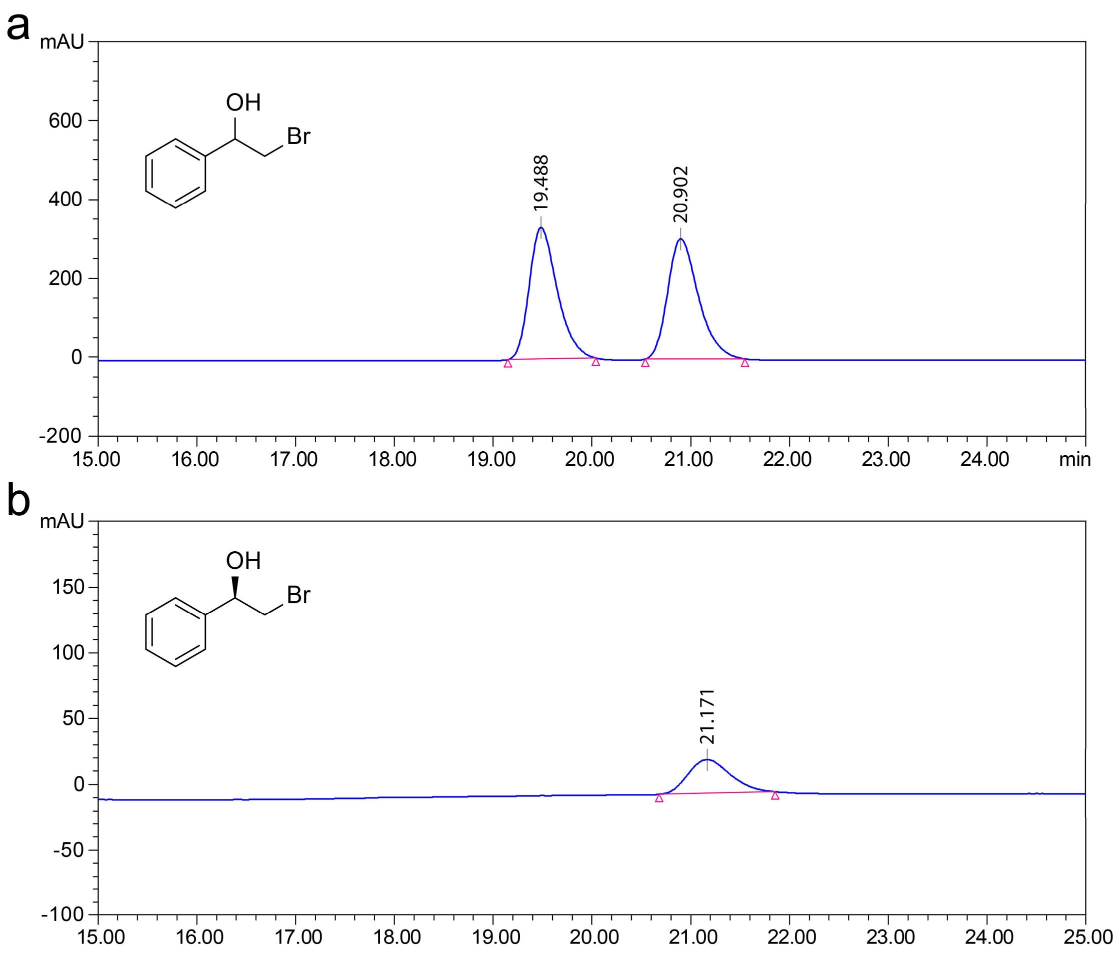

Supplementary Figure 20. HPLC chromatograms for the reduction of substrate **1m**. (a) Racemates made by reduction of **1m** with $NaBH_4$. (b) (*R*)-**2m** prepared by CbAR-H162F variant.

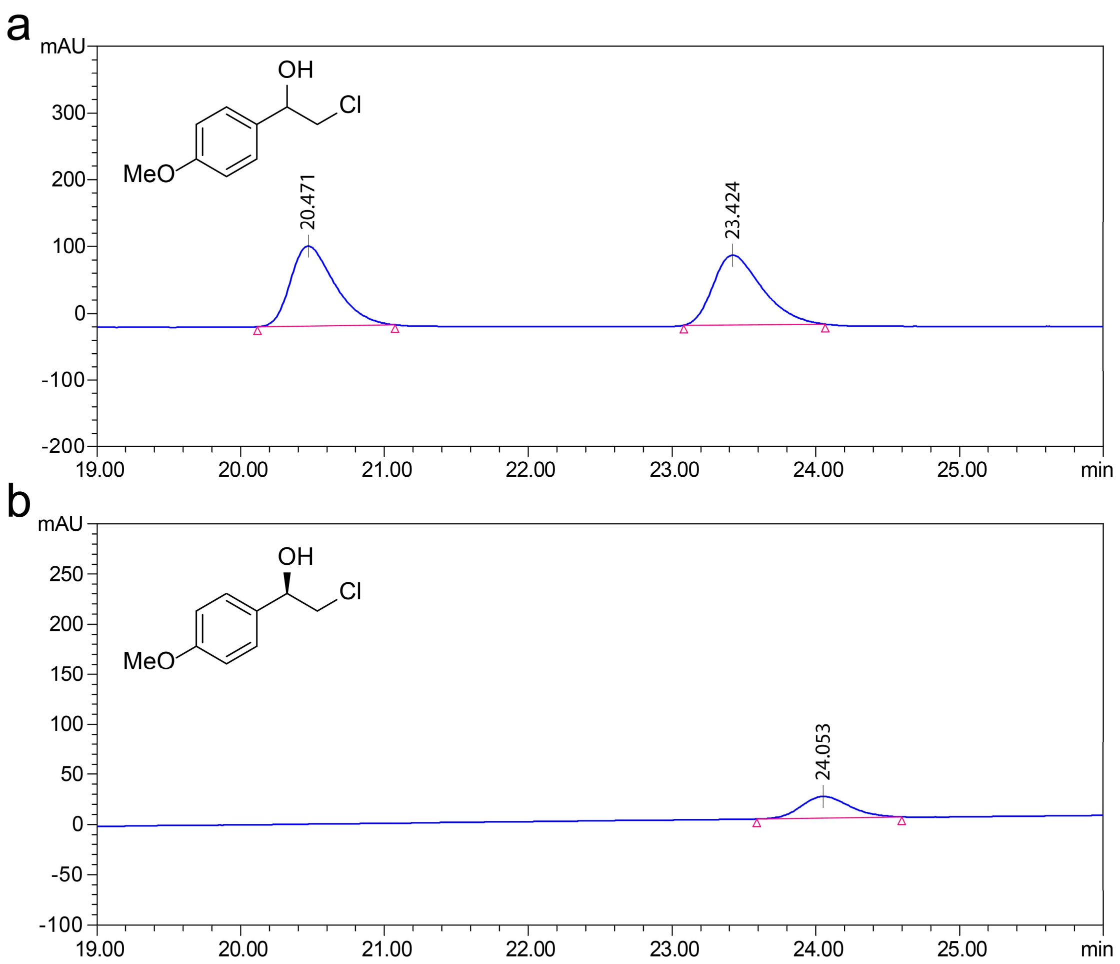

Supplementary Figure 21. HPLC chromatograms for the reduction of substrate **1n**. (a) Racemates made by reduction of **1n** with $NaBH_4$. (b) (*R*)-**2n** prepared by CbAR-H162F variant.

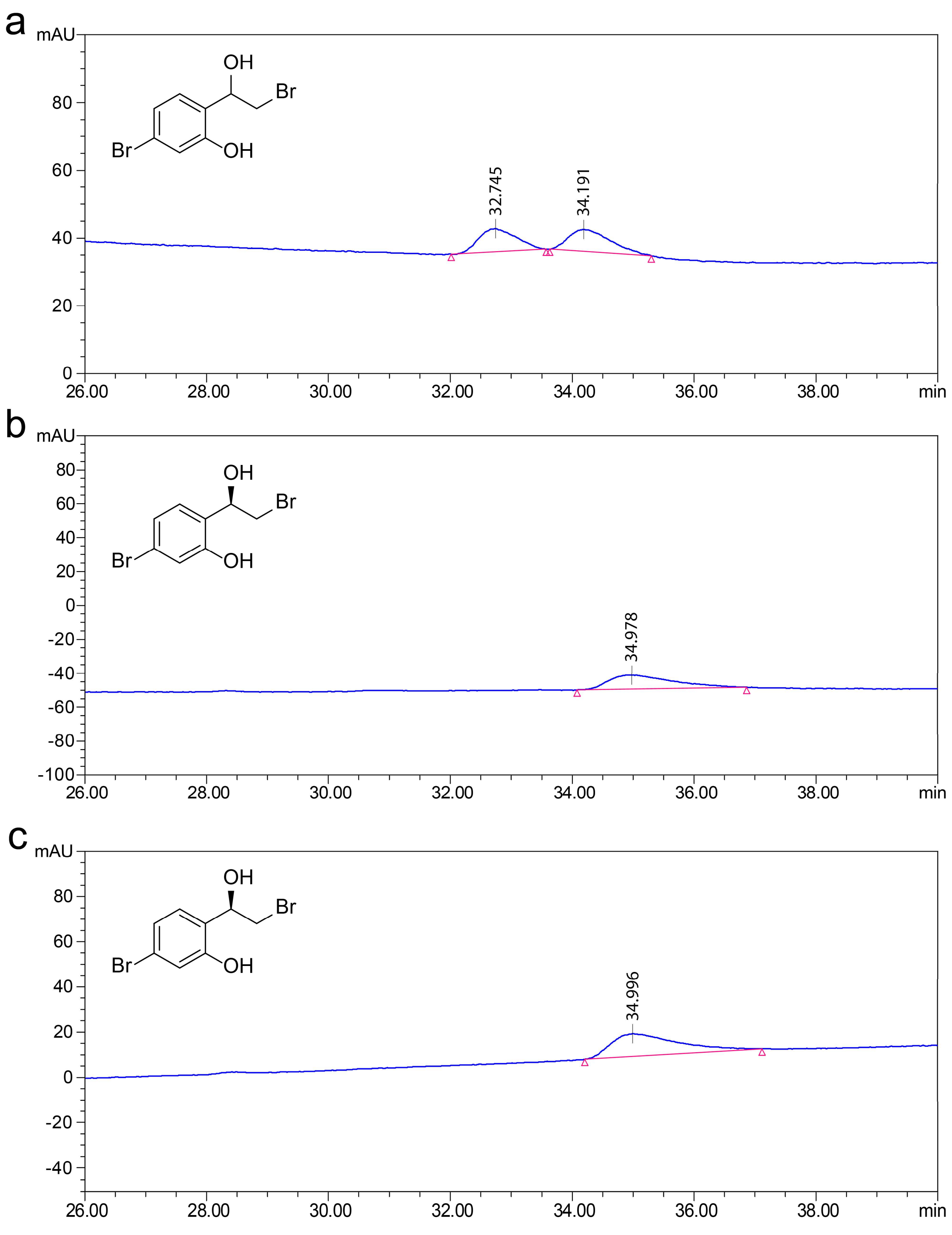

Supplementary Figure 22. HPLC chromatograms for the reduction of substrate **1o**. (a) Racemates made by reduction of **1o** with $NaBH_4$. (b) (*R*)-**2o** prepared by CbAR-H162F variant. (c) (*R*)-**2o** prepared by CbAR.

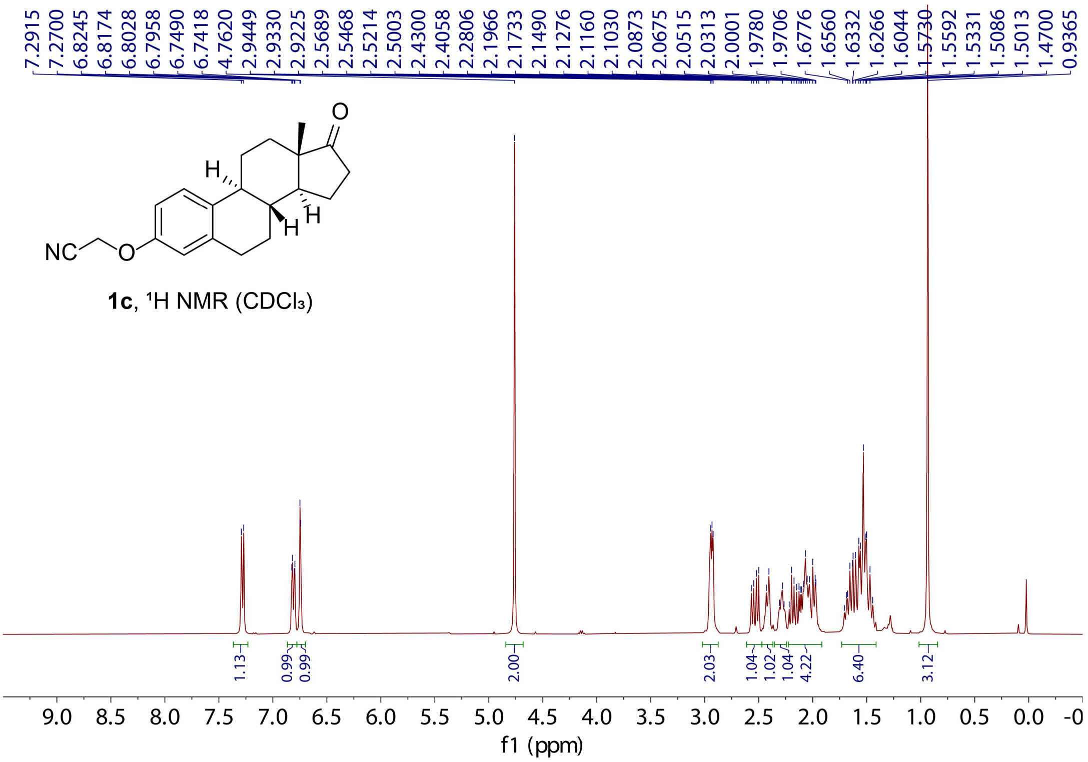

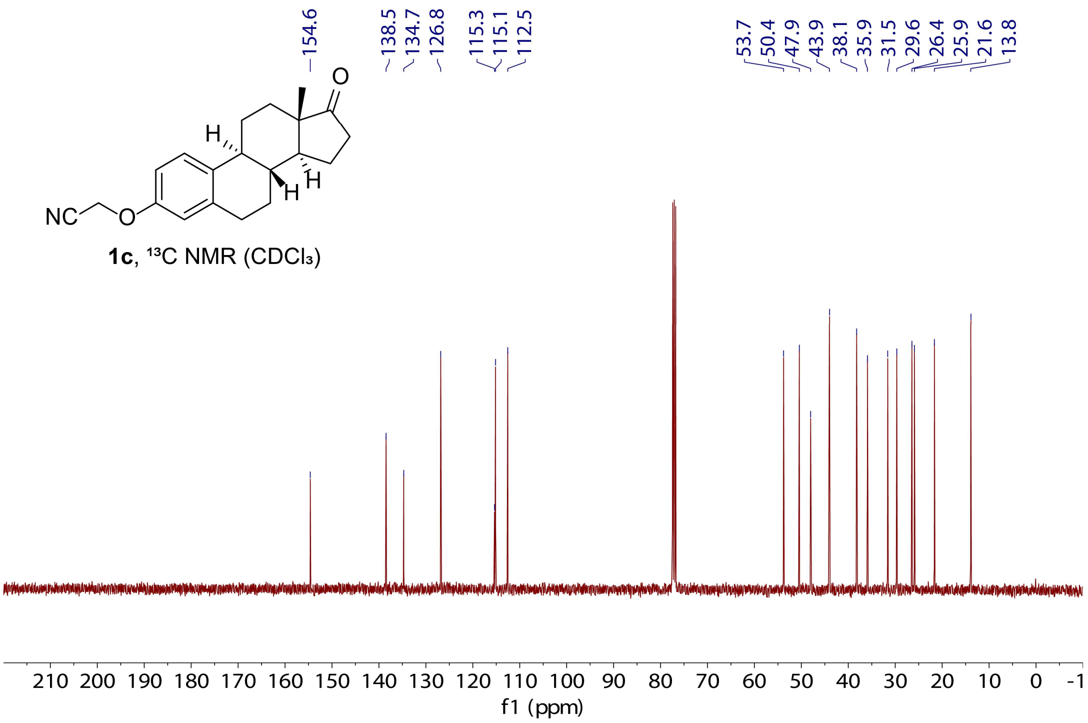

Supplementary Figure 23. ^{1}H (400 MHz) and ^{13}C (101 MHz) NMR spectra of **1c** in $CDCl_3$.

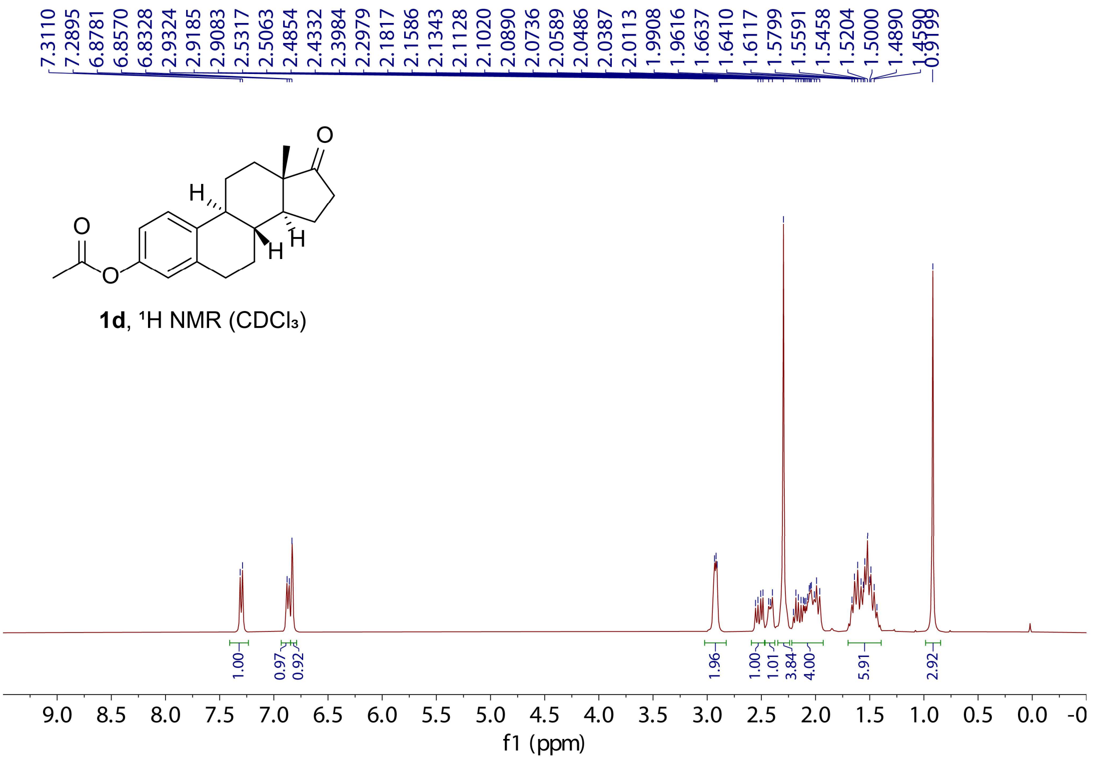

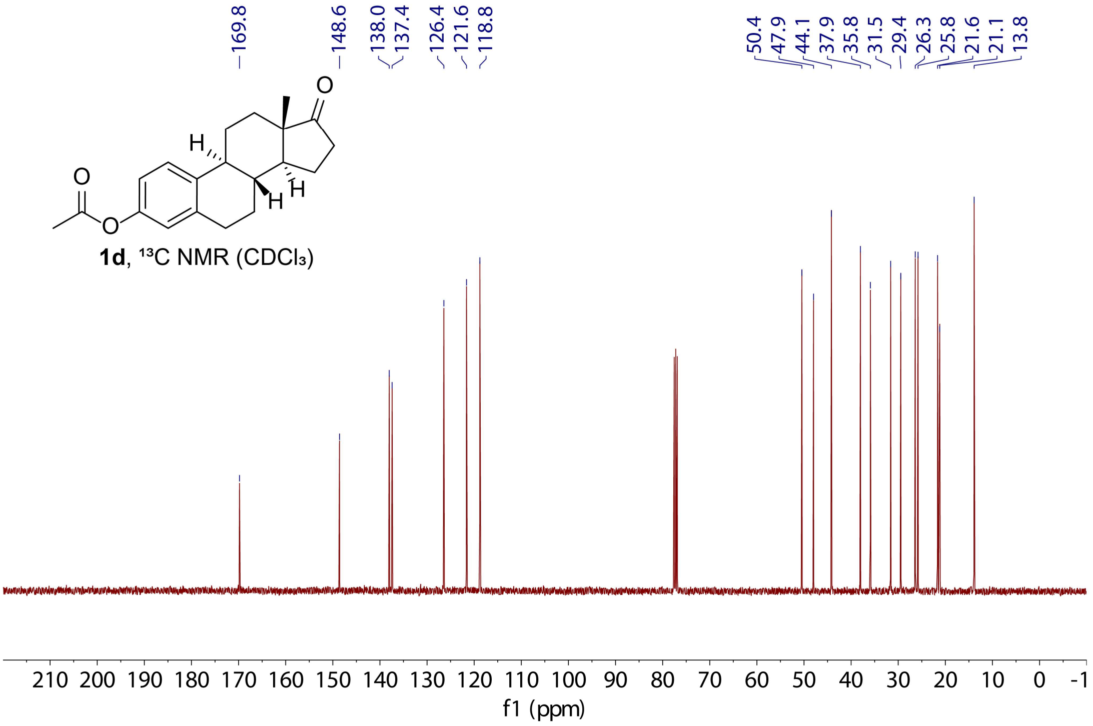

Supplementary Figure 24. ^{1}H (400 MHz) and ^{13}C (101 MHz) NMR spectra of **1d** in $CDCl_3$.

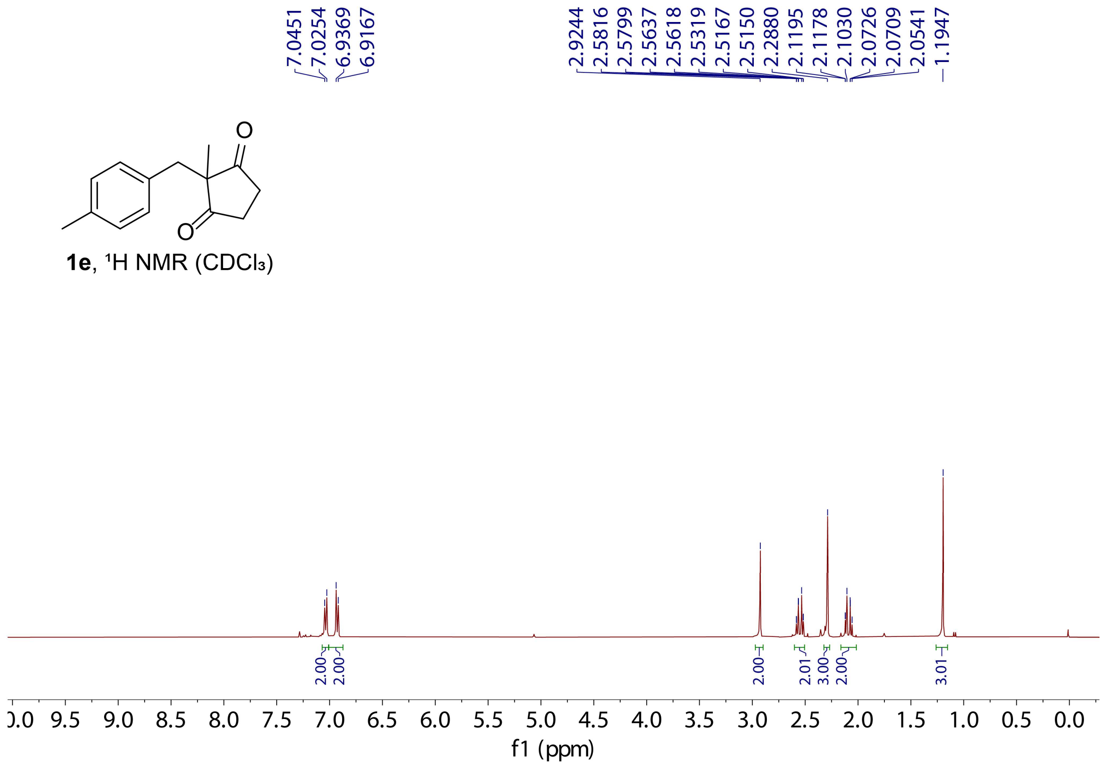

Supplementary Figure 25. ^{1}H (400 MHz) NMR spectrum of **1e** in $CDCl_3$.

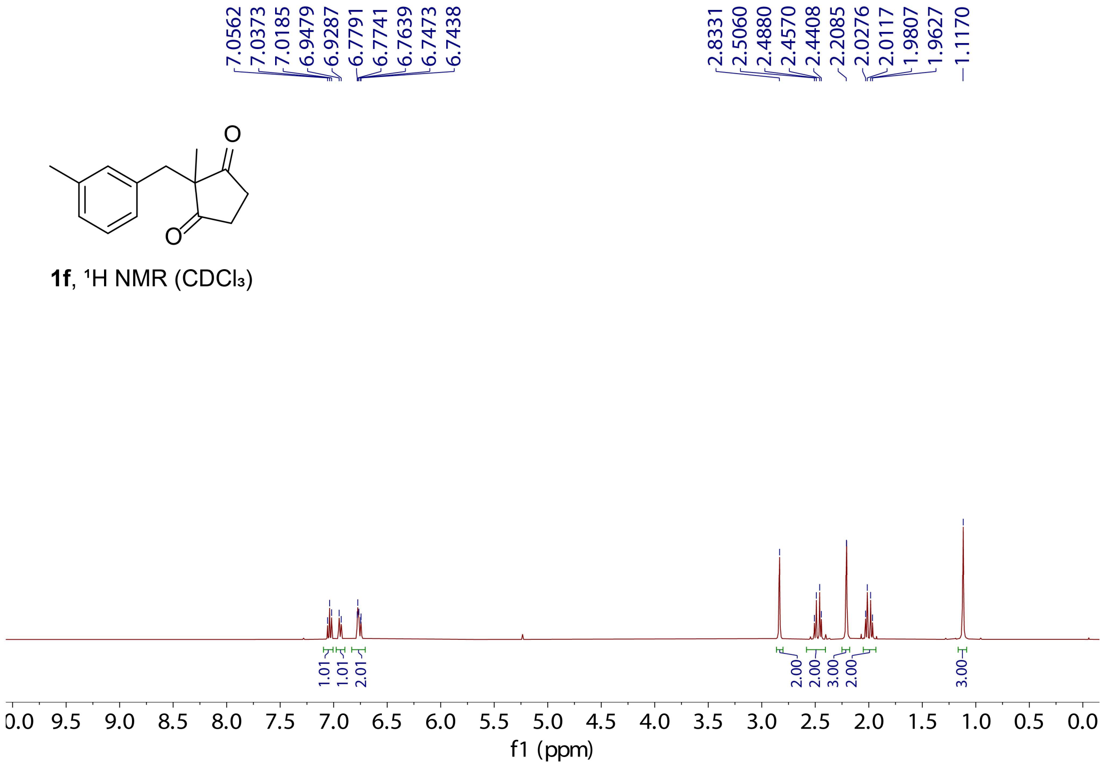

Supplementary Figure 26. ^{1}H (400 MHz) NMR spectrum of **1f** in $CDCl_3$.

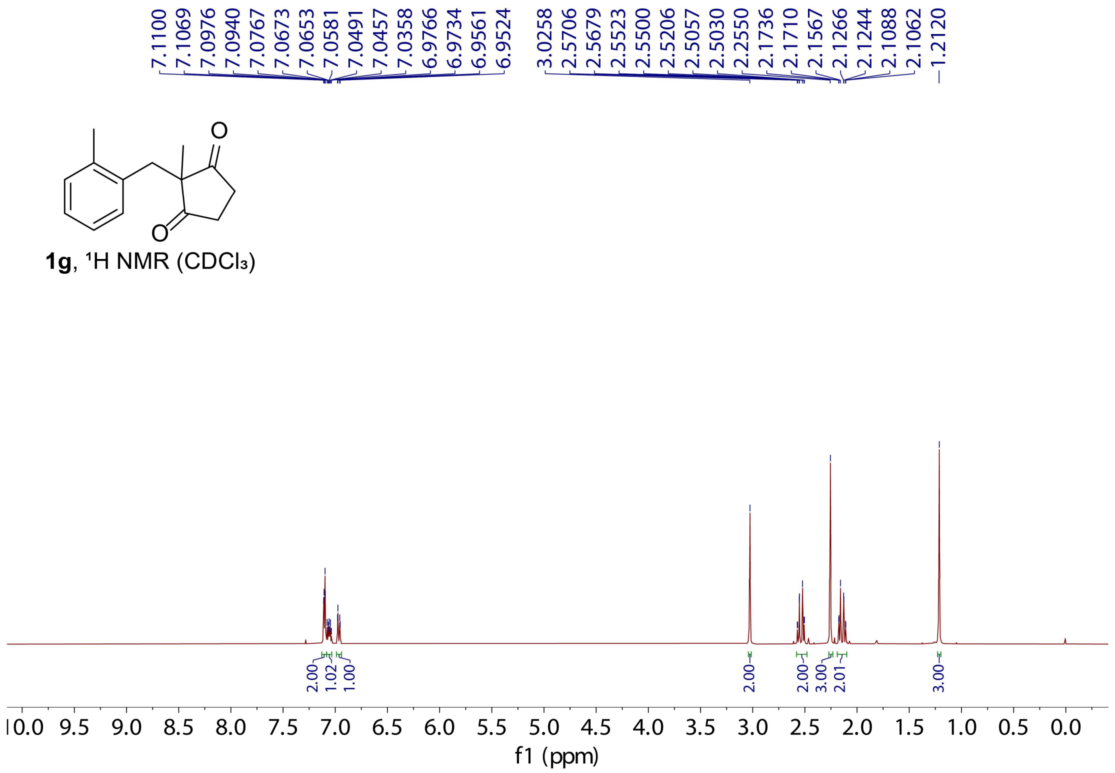

Supplementary Figure 27. ^{1}H (400 MHz) NMR spectrum of **1g** in $CDCl_3$.

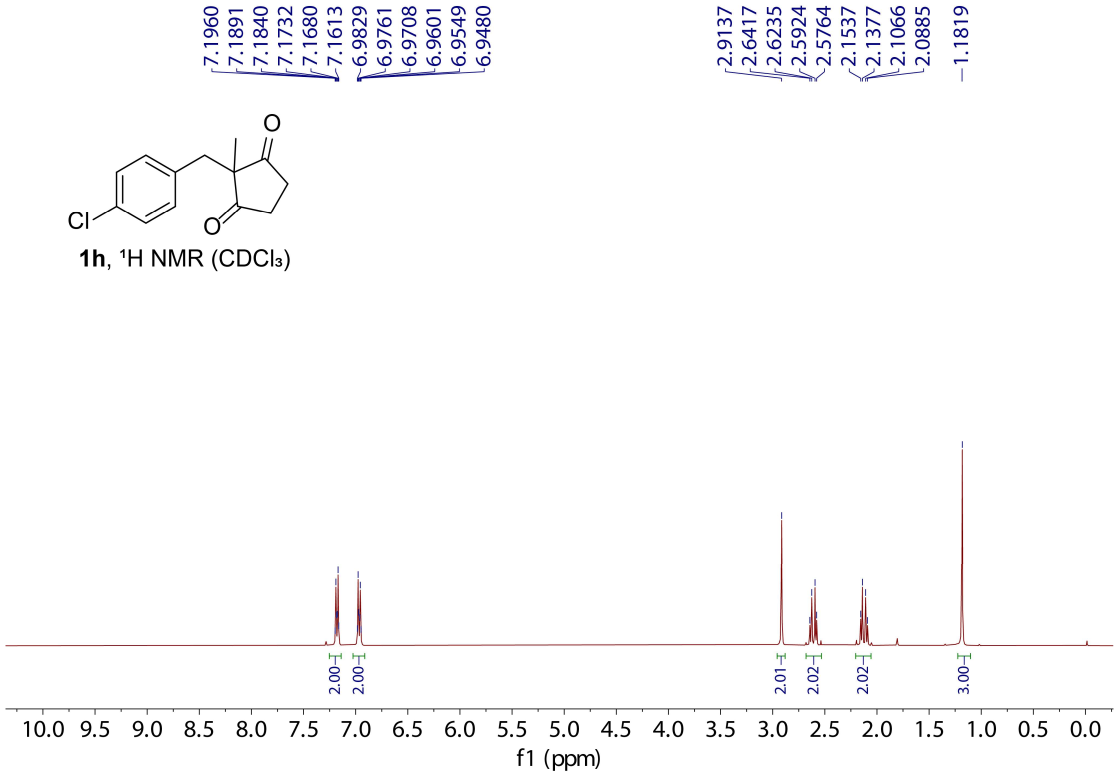

Supplementary Figure 28. ^{1}H (400 MHz) NMR spectrum of **1h** in $CDCl_3$.

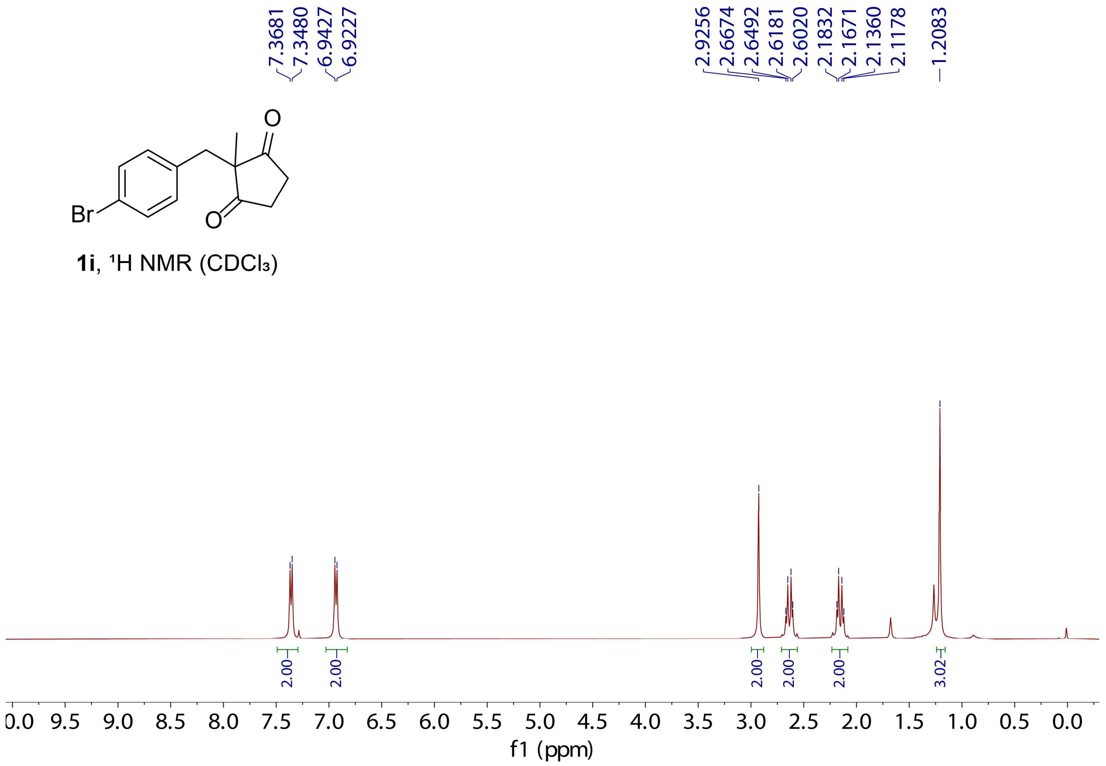

Supplementary Figure 29. ^{1}H (400 MHz) NMR spectrum of **1i** in $CDCl_3$.

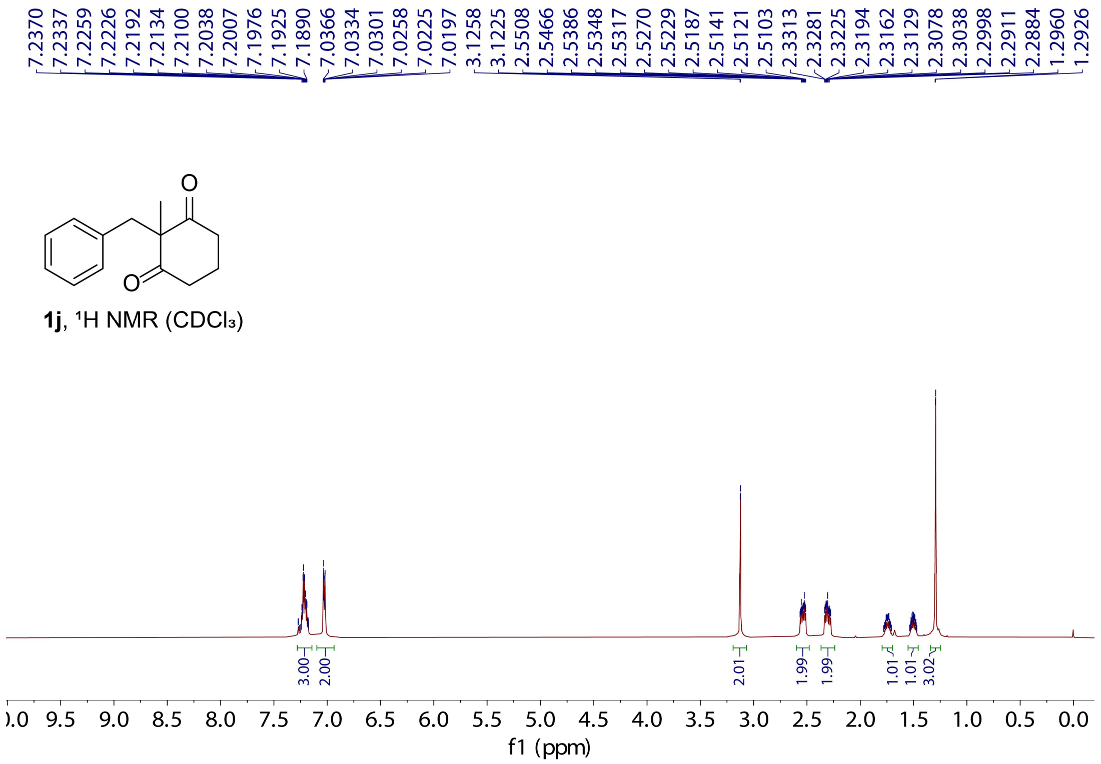

Supplementary Figure 30. ^{1}H (600 MHz) NMR spectrum of **1j** in $CDCl_3$.

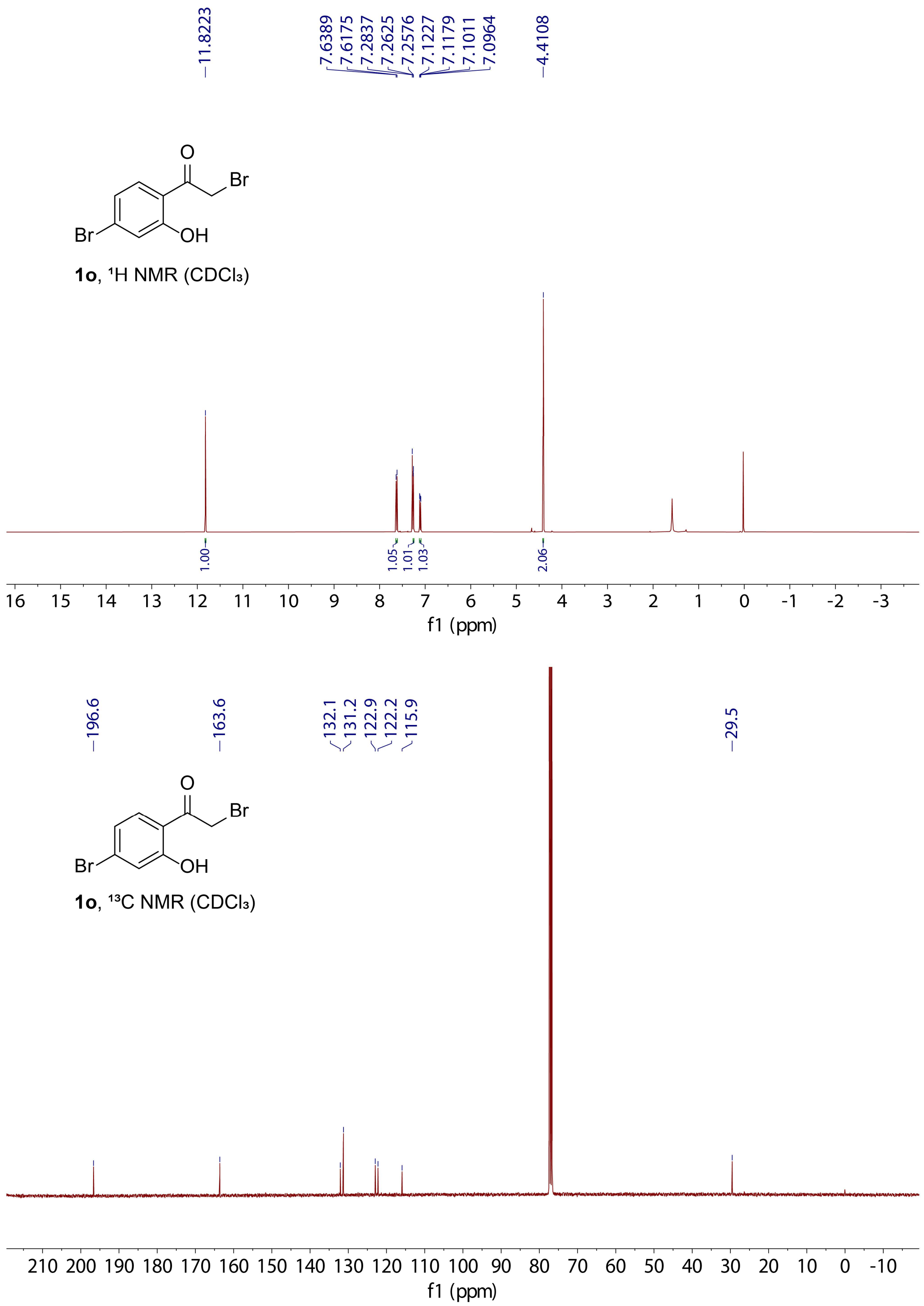

Supplementary Figure 31. ^{1}H (400 MHz) and ^{13}C (101 MHz) NMR spectra of **1o** in $CDCl_3$.

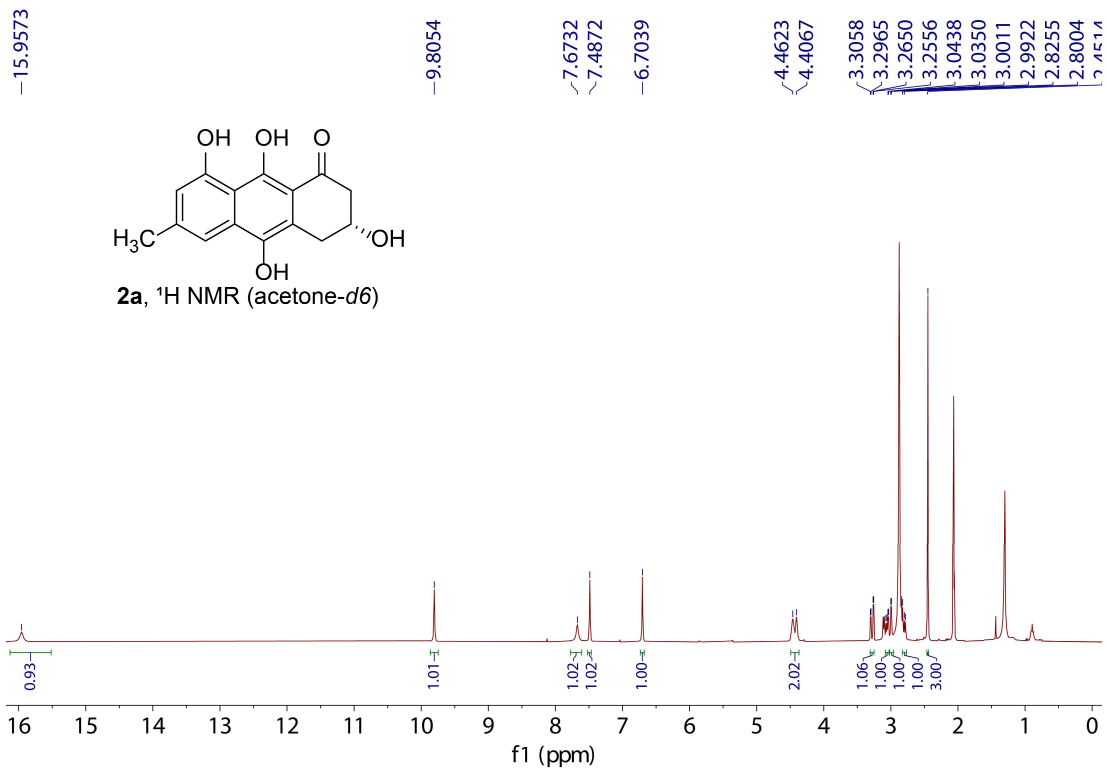

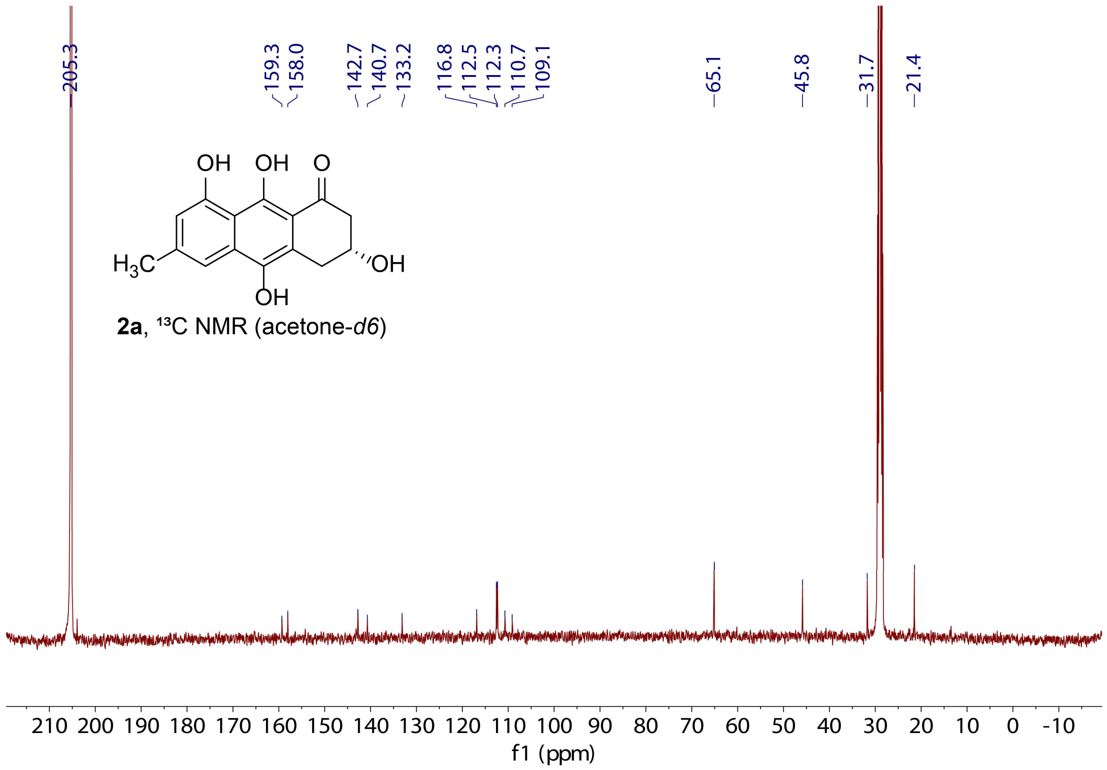

Supplementary Figure 32. ^{1}H (400 MHz) and ^{13}C (101 MHz) NMR spectra of (*R*)-**2a** in acetone-*d*6.

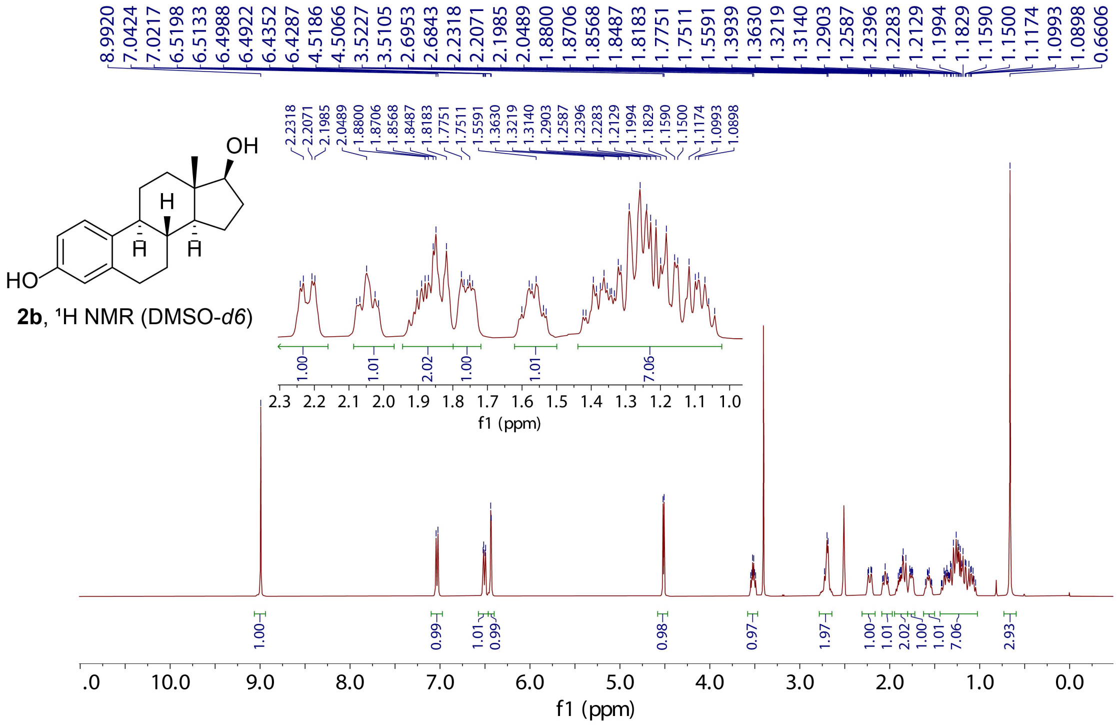

Supplementary Figure 33. ^{1}H (400 MHz) NMR spectrum of **2b** in DMSO-*d*6.

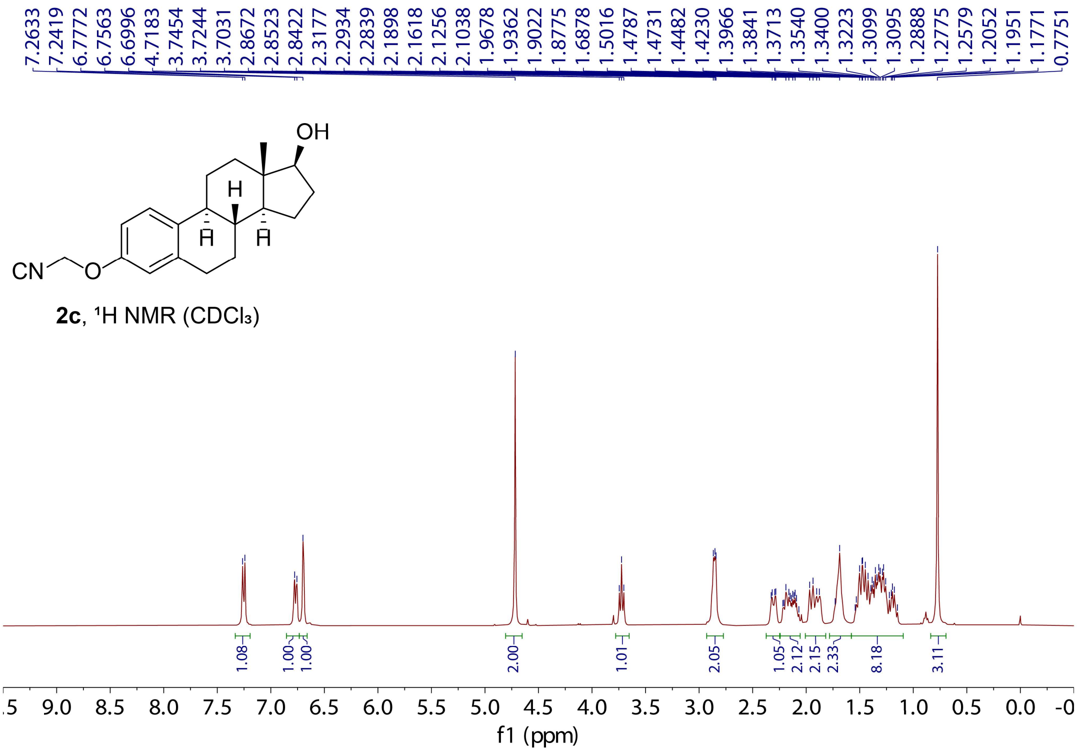

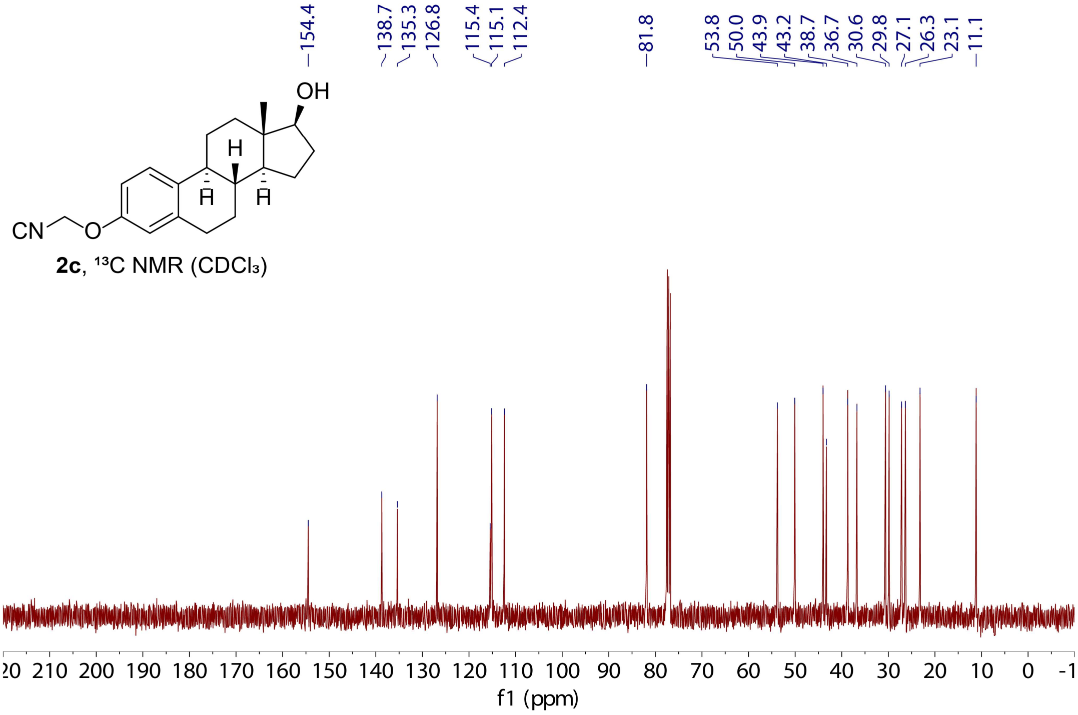

Supplementary Figure 34. ^{1}H (400 MHz) and ^{13}C (101 MHz) NMR spectra of **2c** in $CDCl_3$.

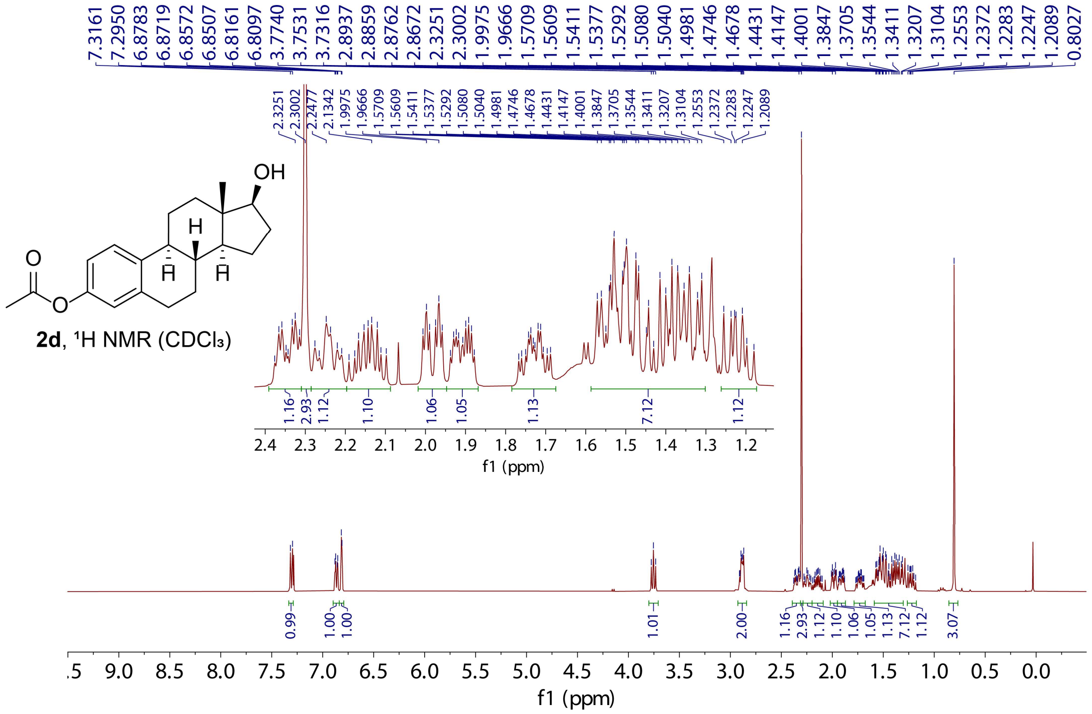

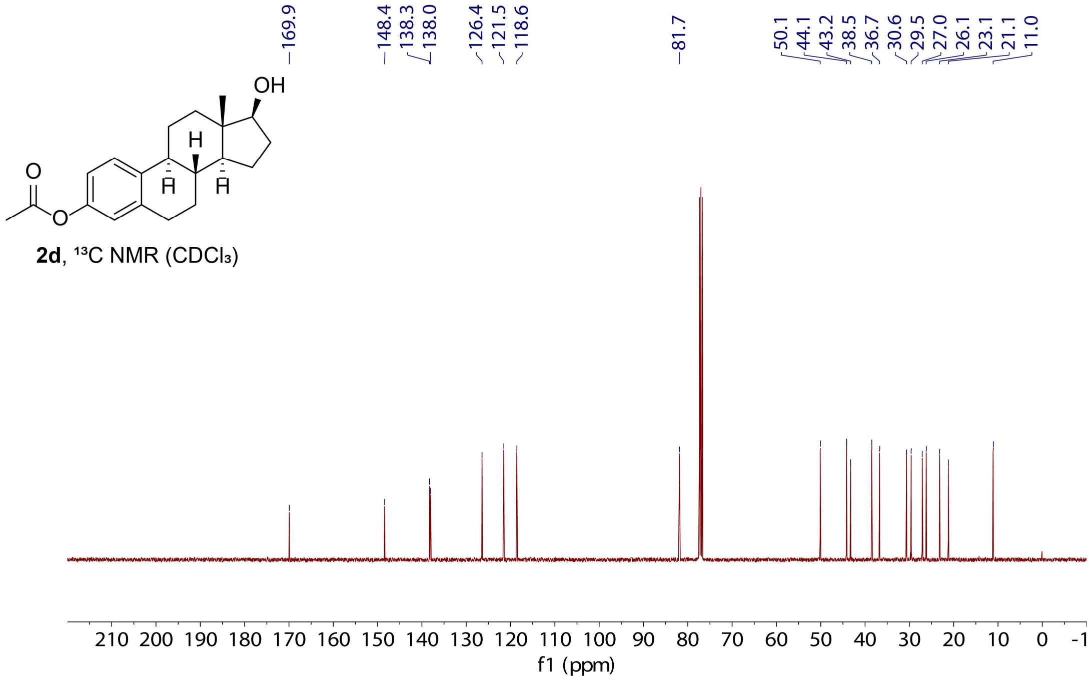

Supplementary Figure 35. ^{1}H (400 MHz) and ^{13}C (101 MHz) NMR spectra of **2d** in $CDCl_3$.

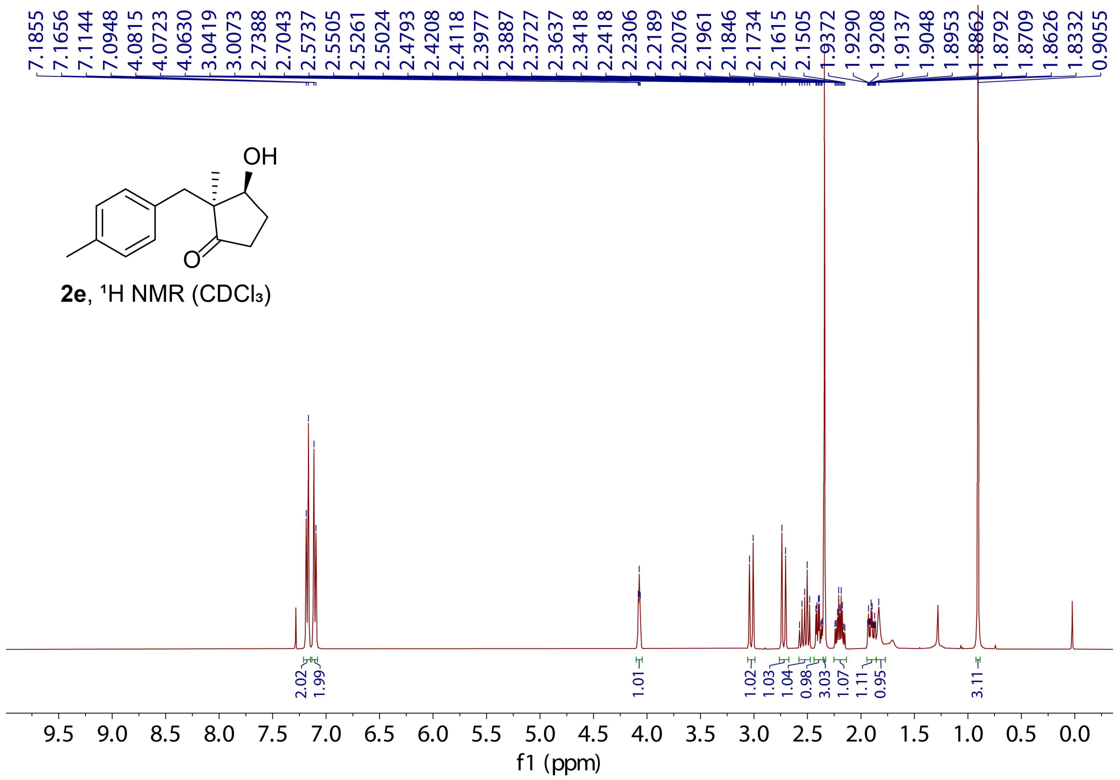

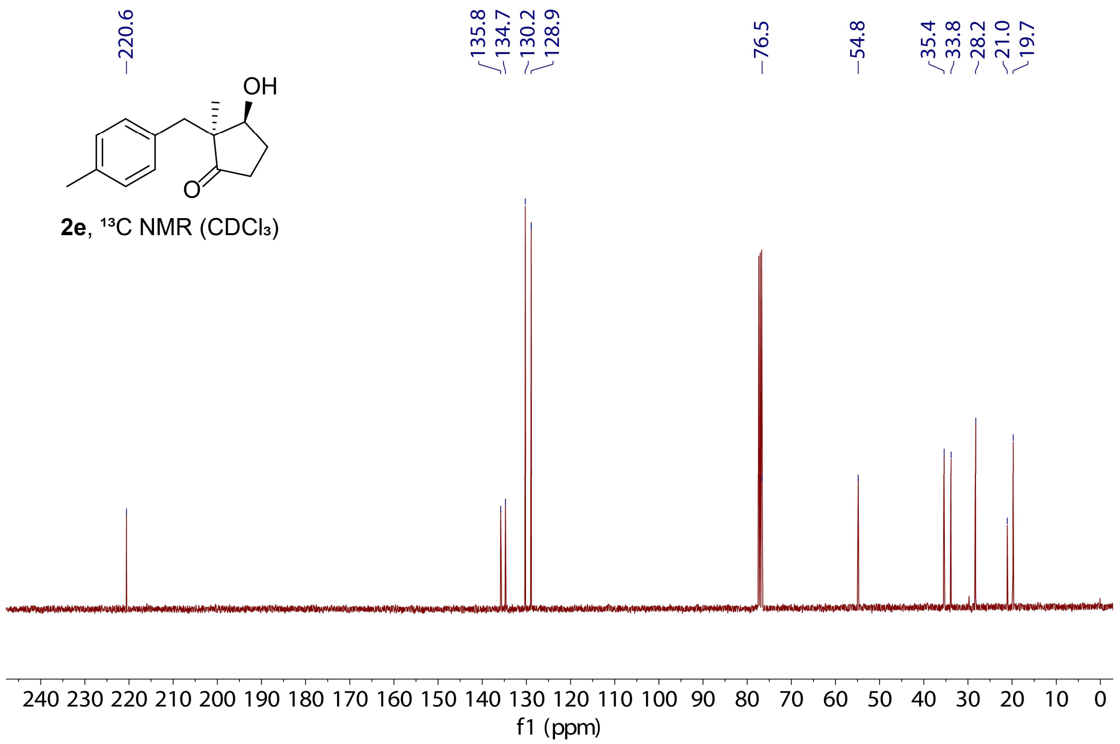

Supplementary Figure 36. ^{1}H (400 MHz) and ^{13}C (101 MHz) NMR spectra of **2e** in $CDCl_3$.

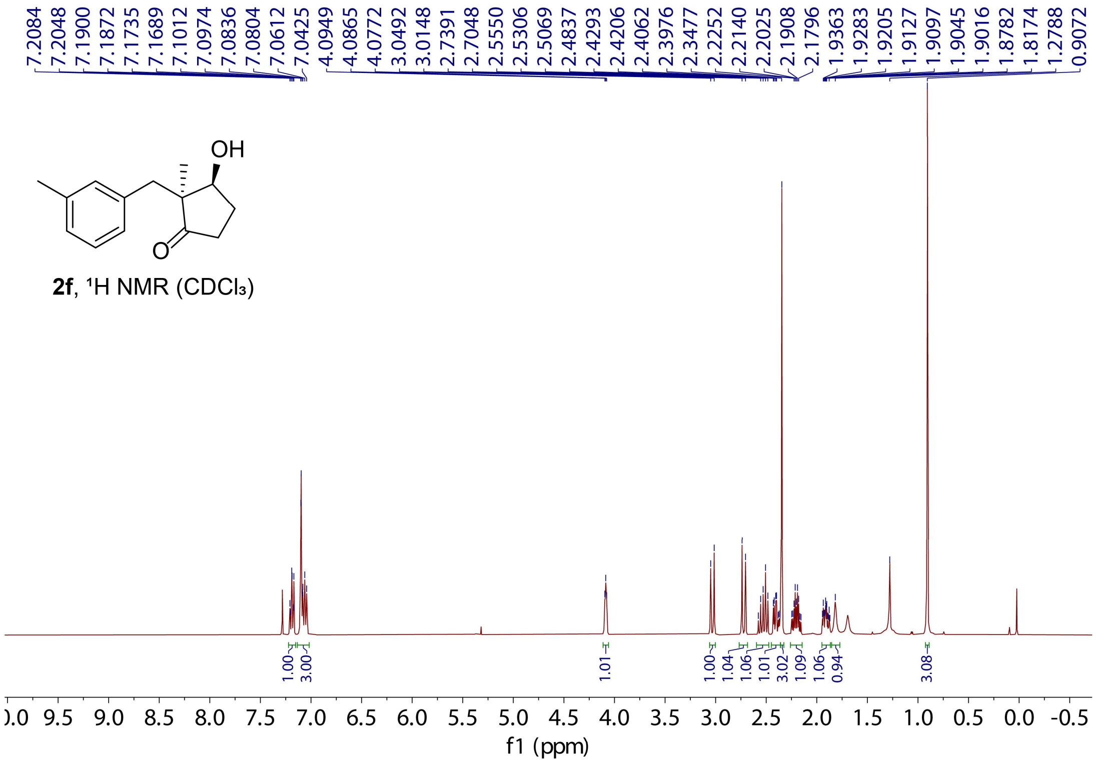

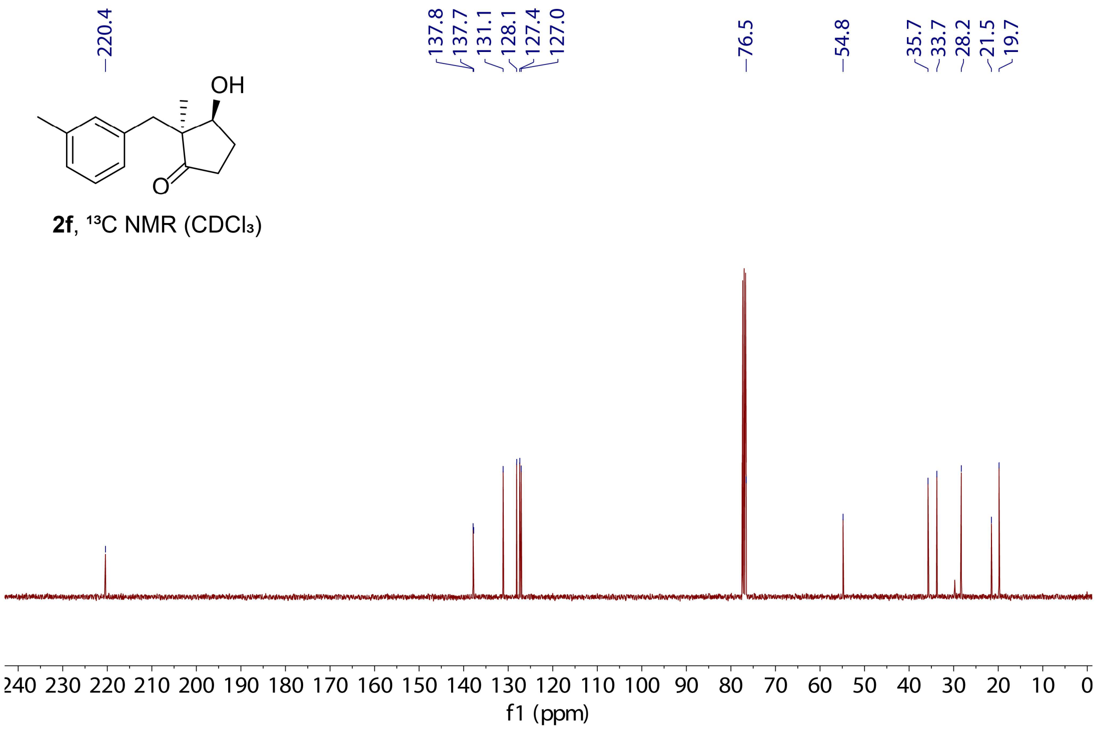

Supplementary Figure 37. ^{1}H (400 MHz) and ^{13}C (101 MHz) NMR spectra of **2f** in $CDCl_3$.

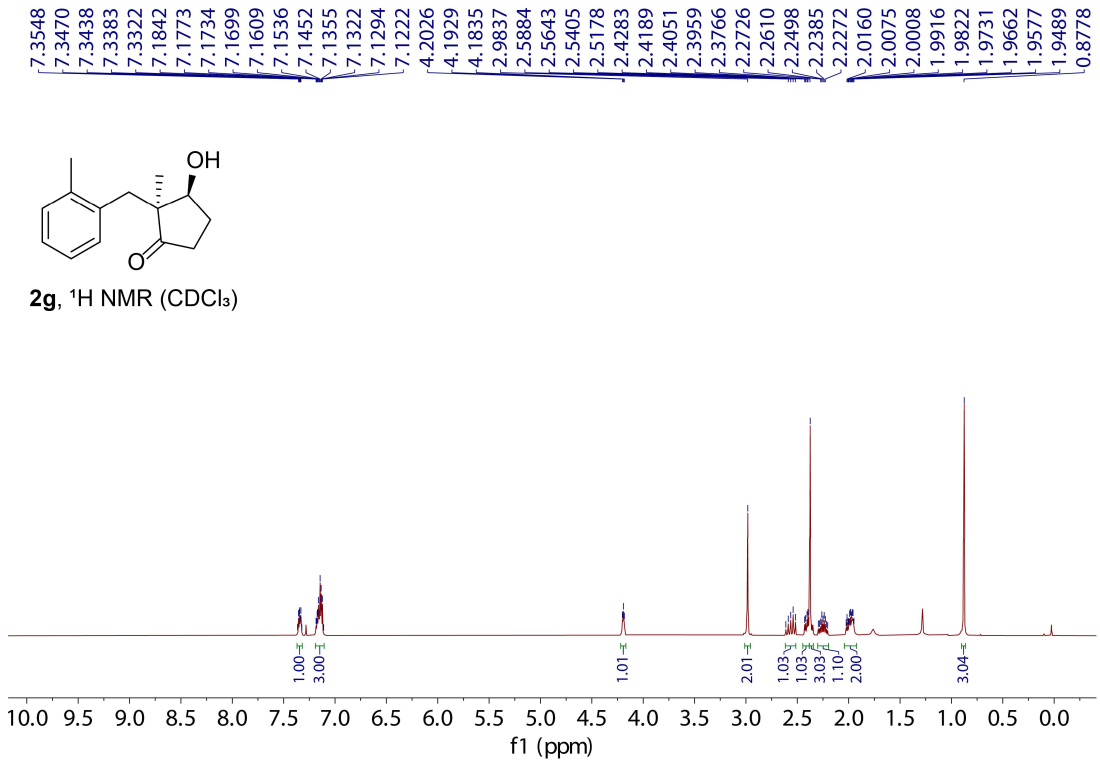

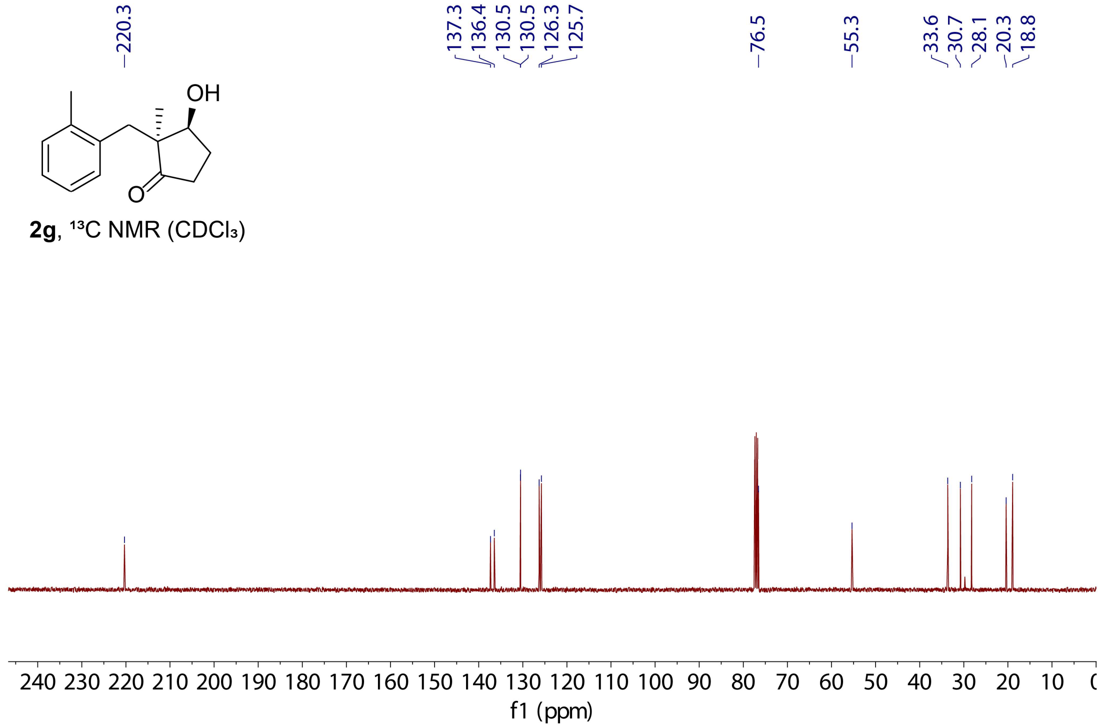

Supplementary Figure 38. ^{1}H (400 MHz) and ^{13}C (101 MHz) NMR spectra of **2g** in $CDCl_3$.

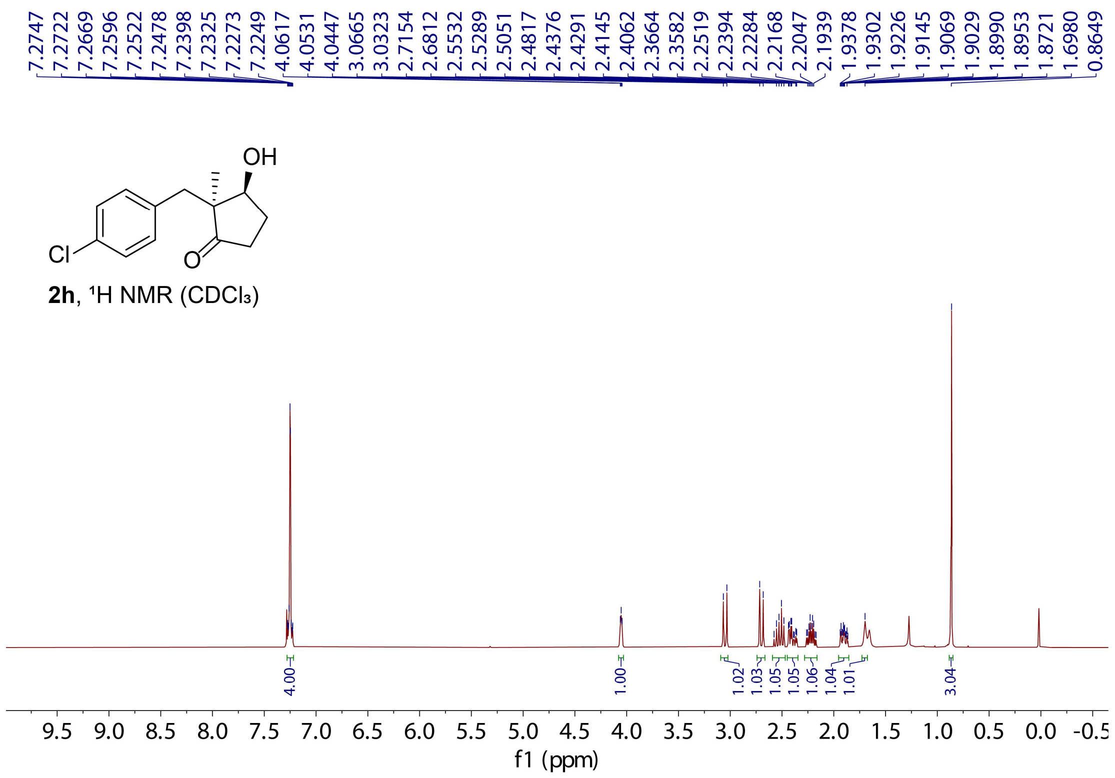

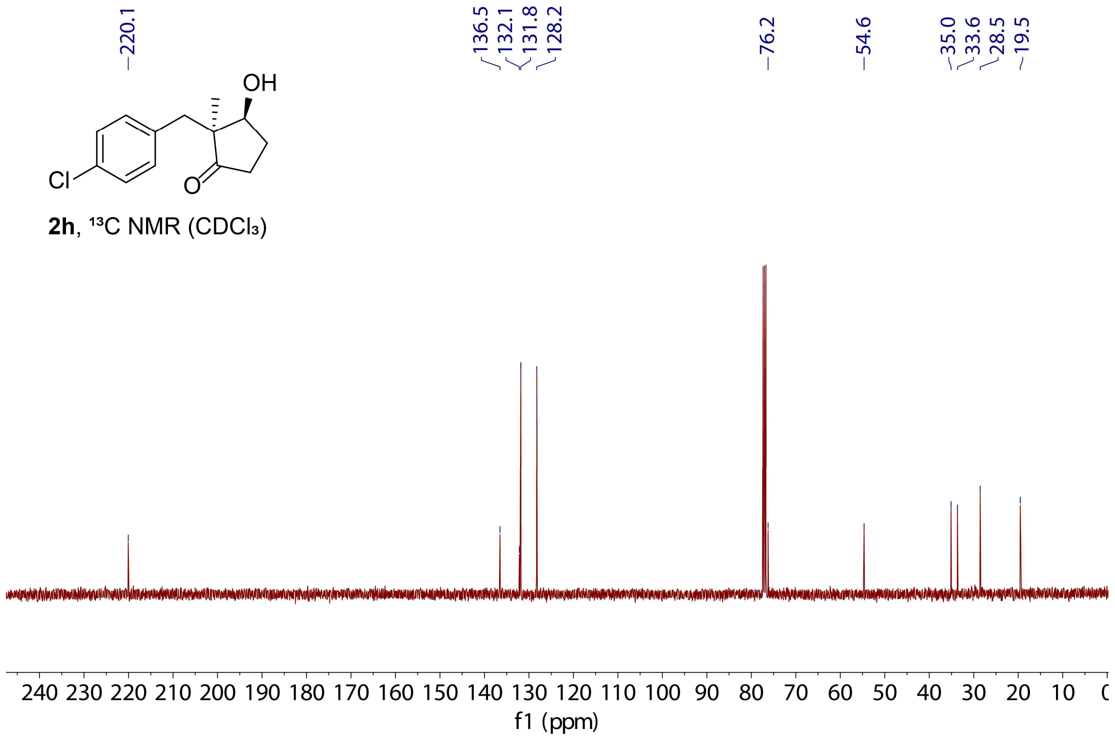

Supplementary Figure 39. ^{1}H (400 MHz) and ^{13}C (101 MHz) NMR spectra of **2h** in $CDCl_3$.

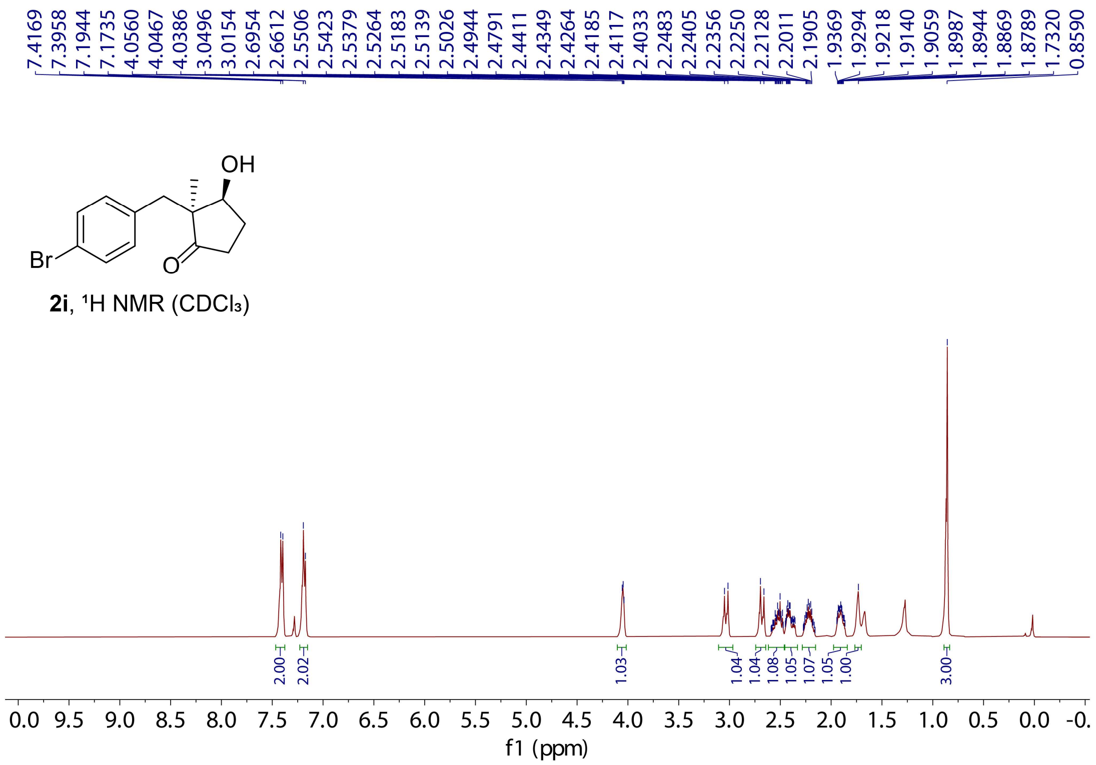

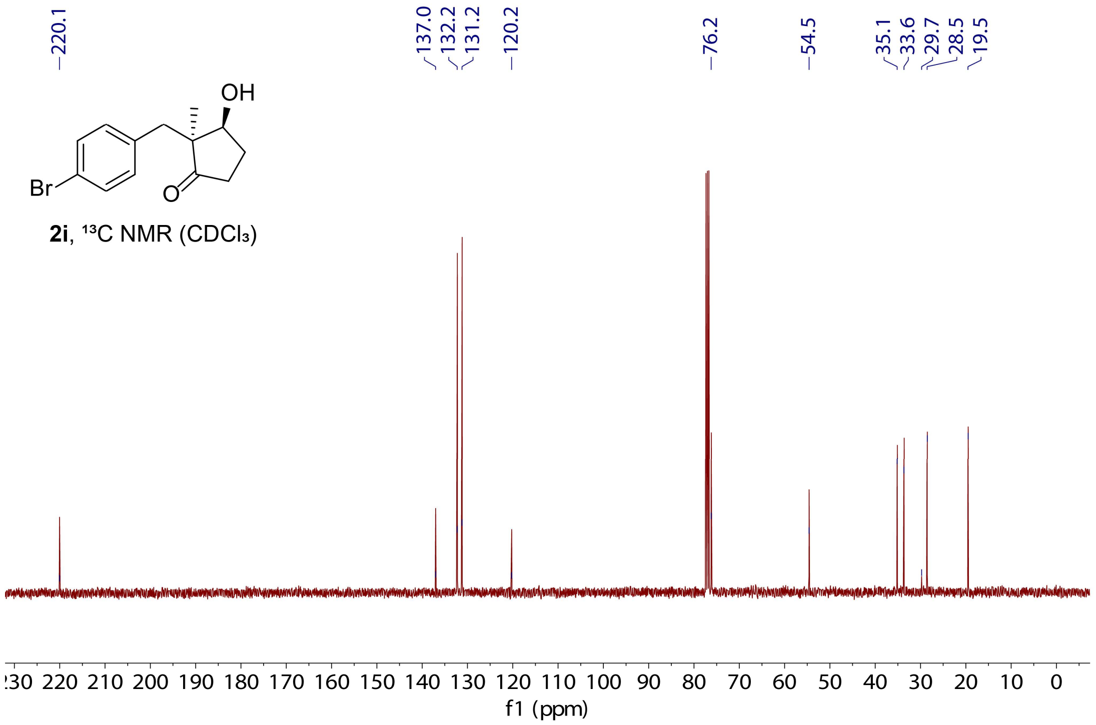

Supplementary Figure 40. ^{1}H (400 MHz) and ^{13}C (101 MHz) NMR spectra of **2i** in $CDCl_3$.

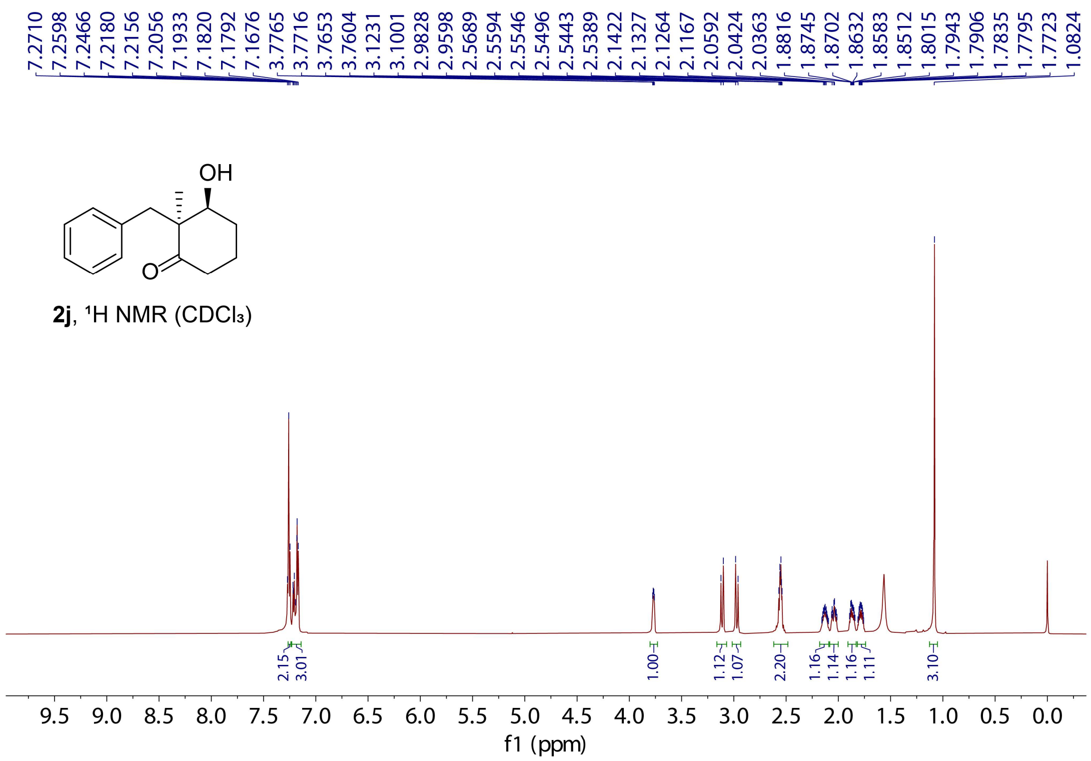

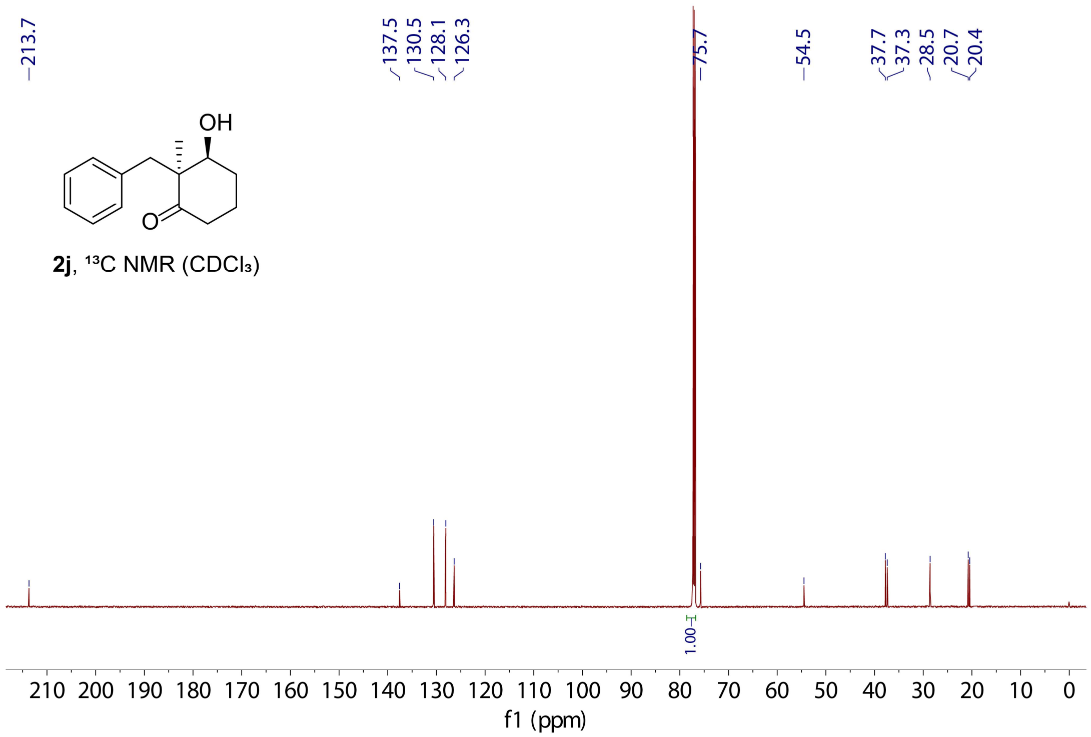

Supplementary Figure 41. ^{1}H (600 MHz) and ^{13}C (151 MHz) NMR spectra of **2j** in $CDCl_3$.

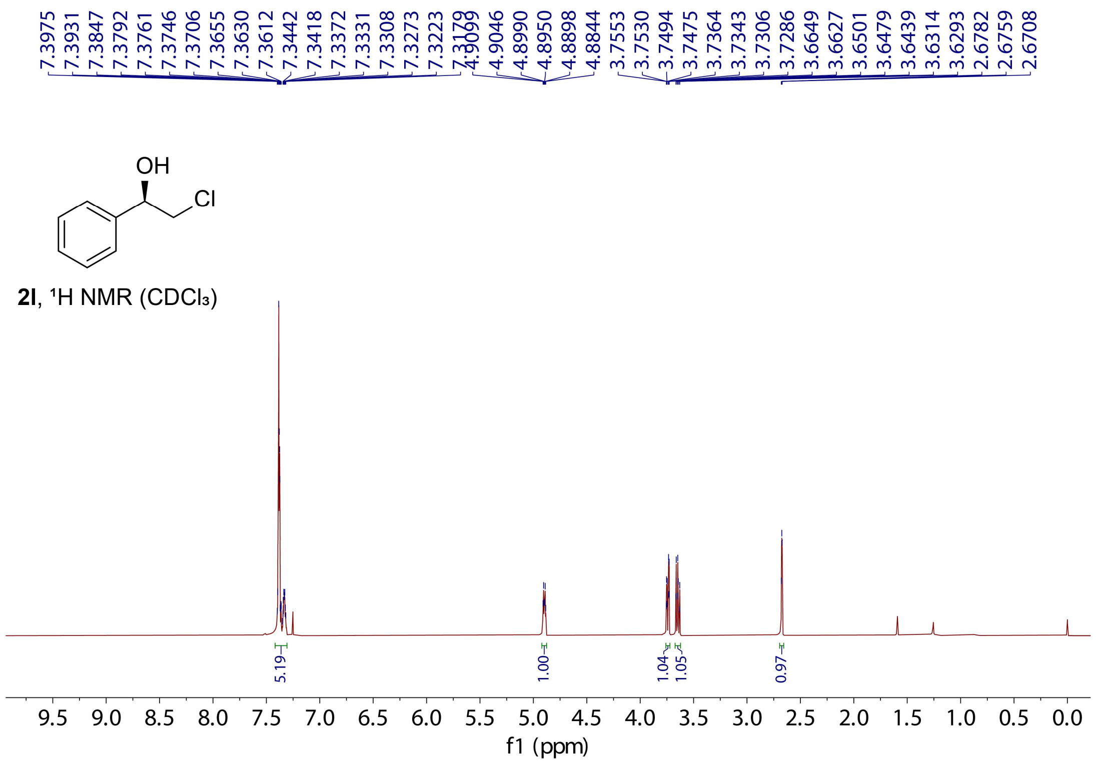

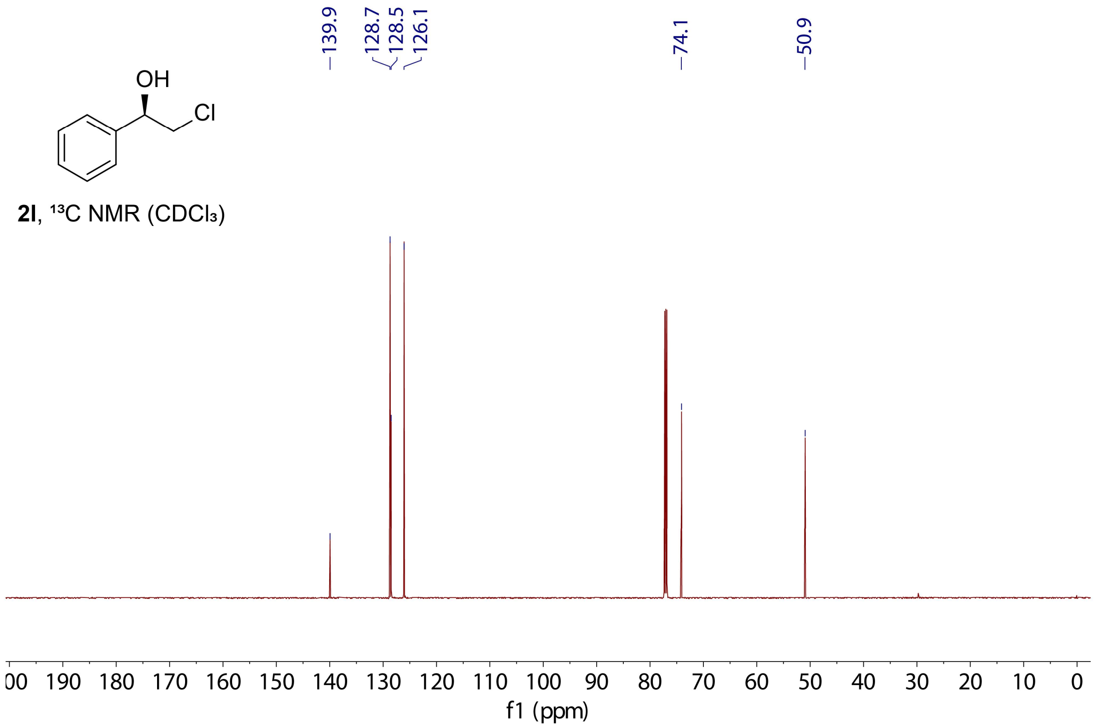

Supplementary Figure 42. ^{1}H (600 MHz) and ^{13}C (151 MHz) NMR spectra of **2l** in $CDCl_3$.

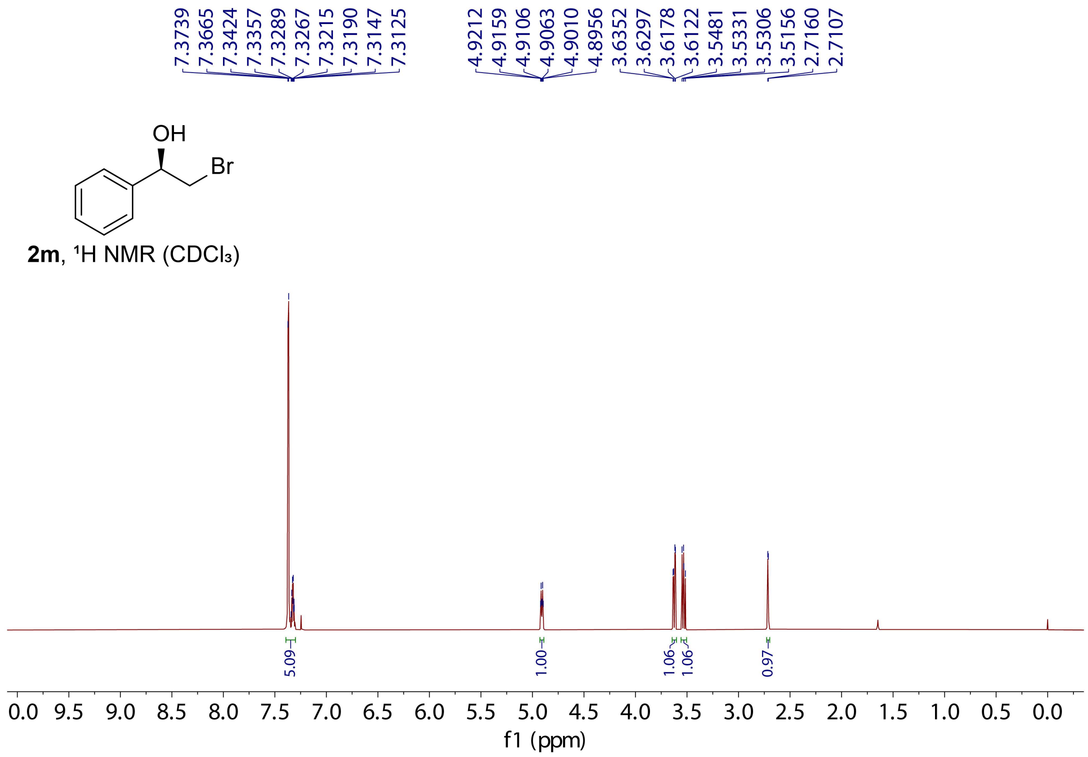

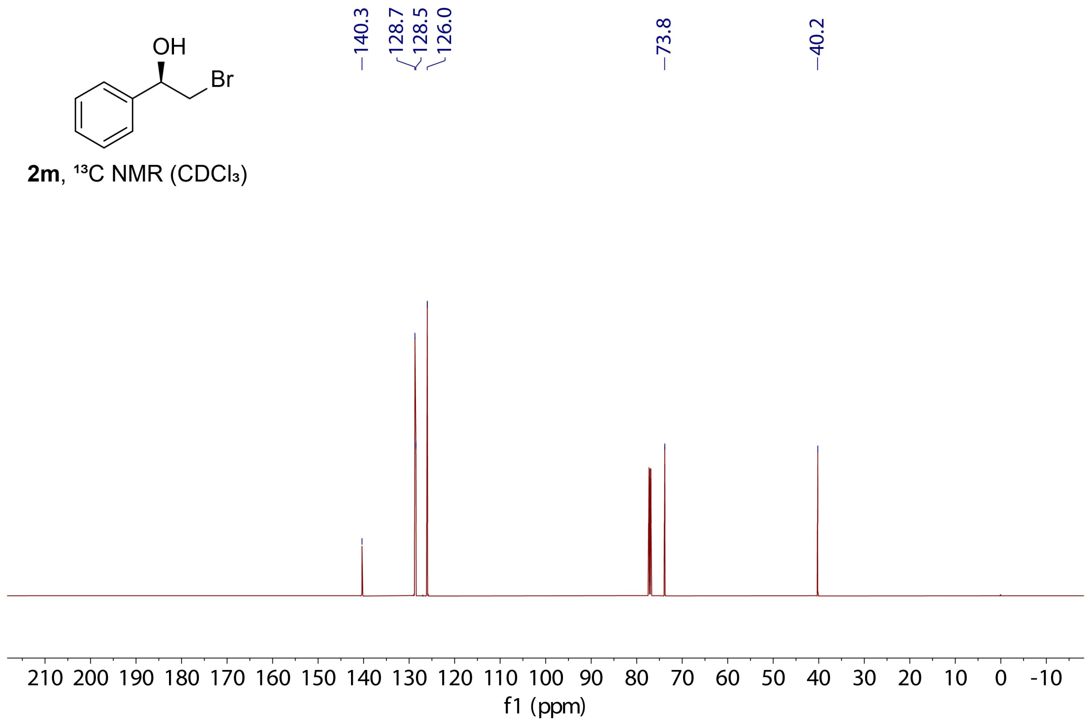

Supplementary Figure 43. ^{1}H (600 MHz) and ^{13}C (151 MHz) NMR spectra of **2m** in $CDCl_3$.

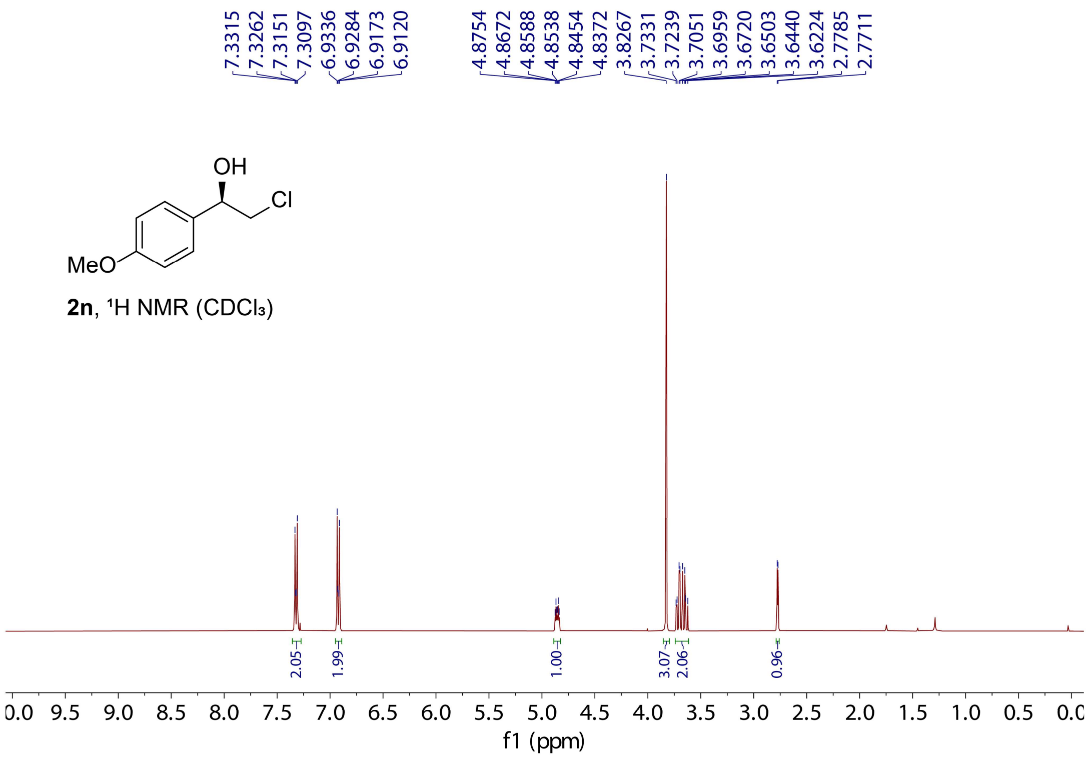

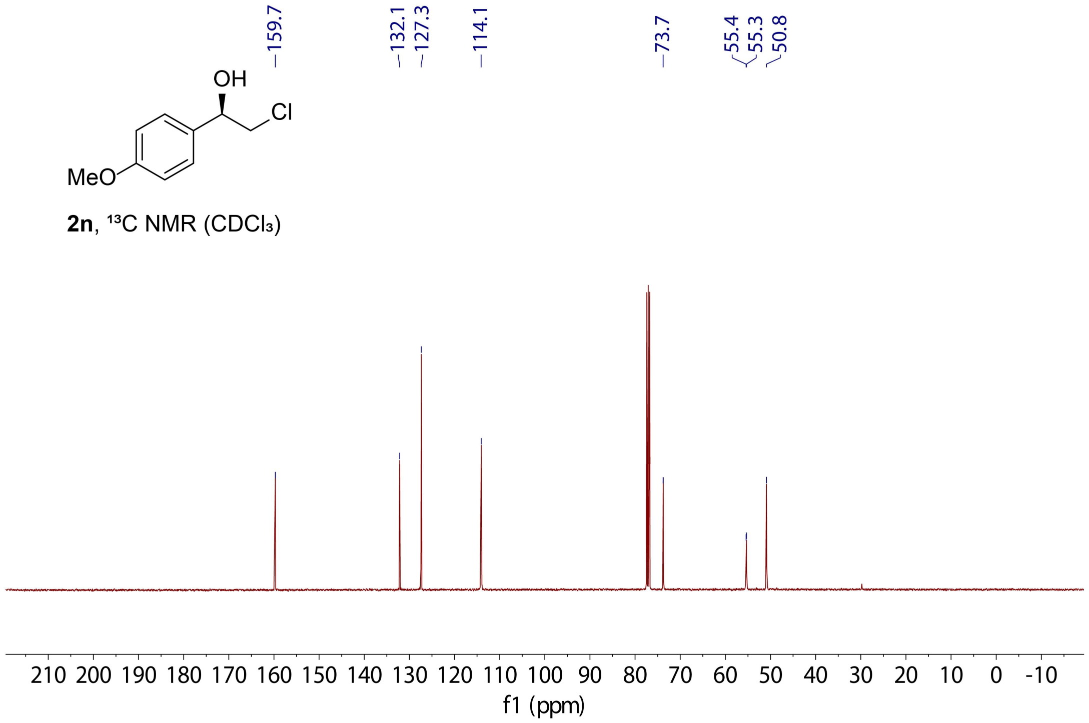

Supplementary Figure 44. ^{1}H (400 MHz) and ^{13}C (101 MHz) NMR spectra of **2n** in $CDCl_3$.

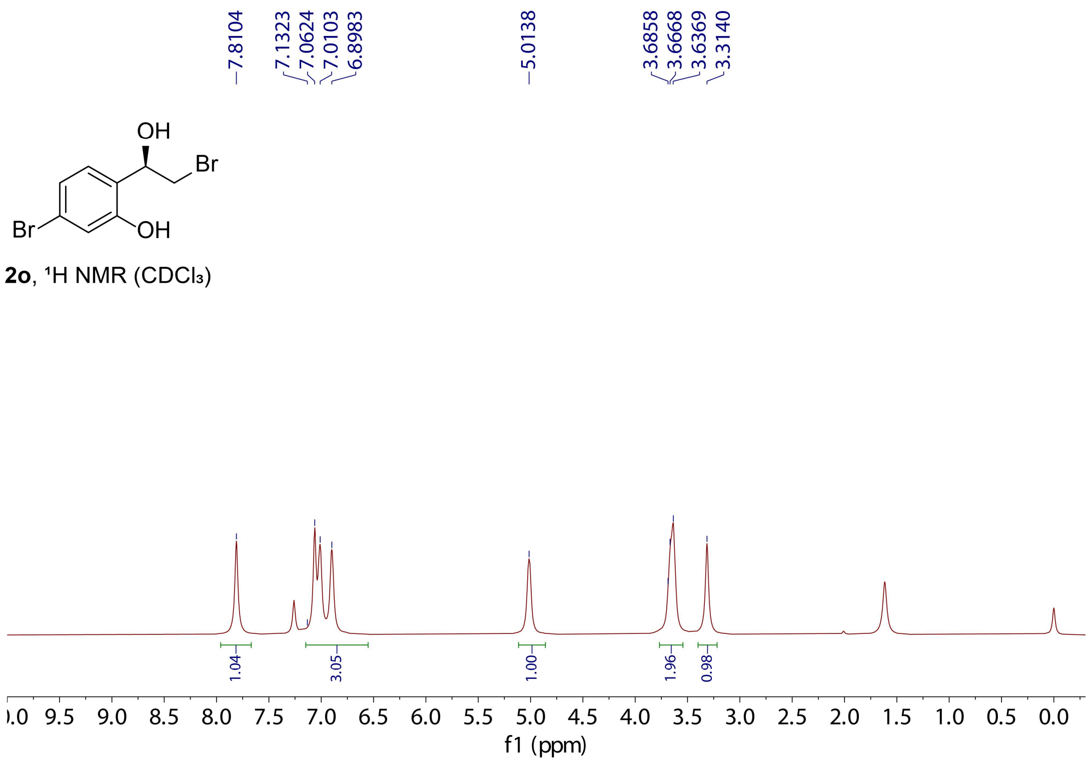

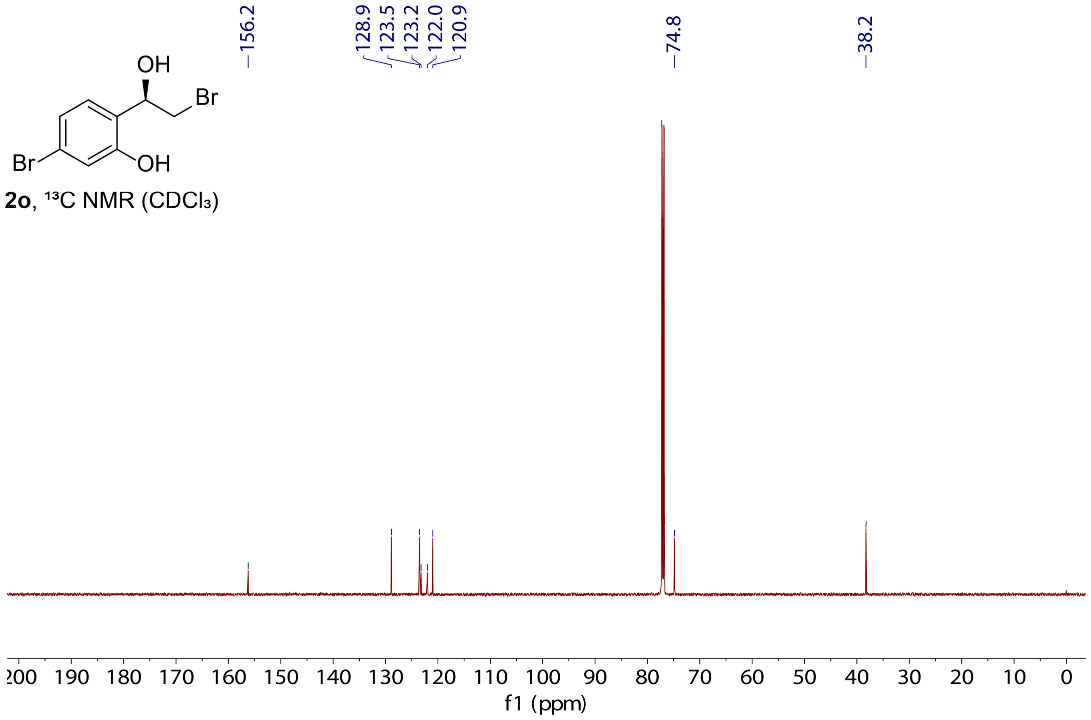

Supplementary Figure 45. ^{1}H (600 MHz) and ^{13}C (151 MHz) NMR spectra of **2o** in $CDCl_3$.
