# Supplementary material for: Structural analysis of an anthrol reductase inspires enantioselective synthesis of enantiopure hydroxycycloketones and β-halohydrins
Source: Nat Commun. 2023 Jan 21;14:353. doi: 10.1038/s41467-023-36064-4 (PMC9867772; doi:10.1038/s41467-023-36064-4)
Supplement: Supplementary file 3 — Description of Additional Supplementary Files [file 41467_2023_36064_MOESM3_ESM.pdf]

Description of Additional Supplementary Files

Supplementary Data 1: Cartesian coordinates
